# Evolutionary Signatures of Common Human *Cis*-Regulatory Haplotypes

**DOI:** 10.1371/journal.pone.0003362

**Published:** 2008-10-10

**Authors:** Ching Ouyang, David D. Smith, Theodore G. Krontiris

**Affiliations:** 1 Division of Molecular Medicine, Beckman Research Institute of the City of Hope, Duarte, California, United States of America; 2 Division of Information Sciences, Beckman Research Institute of the City of Hope, Duarte, California, United States of America; University of Cape Town, South Africa

## Abstract

Variation in gene expression may give rise to a significant fraction of inter-individual phenotypic variation. Studies searching for the underlying genetic controls for such variation have been conducted in model organisms and humans in recent years. In our previous effort of assessing conserved underlying haplotype patterns across ethnic populations, we constructed common haplotypes using SNPs having conserved linkage disequilibrium (LD) across ethnic populations. These common haplotypes cluster into a simple evolutionary structure based on their frequencies, defining only up to three conserved clusters termed ‘haplotype frameworks’. One intriguing preliminary finding was that a significant portion of reported variants strongly associated with *cis*-regulation tags these globally conserved haplotype frameworks. Here we expand the investigation by collecting genes showing stringently determined *cis*-association between genotypes and expression phenotypes from major studies. We conducted phylogenetic analysis of current major haplotypes along with the corresponding haplotypes derived from chimpanzee reference sequences. Our analysis reveals that, for the vast majority of such *cis*-regulatory genes, the tagging SNPs showing the strongest association also tag the haplotype lineages directly separated from ancestry, inferred from either chimpanzee reference sequences or the allele frequency-derived haplotype frameworks, suggesting that the differentially expressed phenotypes were evolved relatively early in human history. Such evolutionary signatures provide keys for a more effective identification of globally-conserved candidate regulatory haplotypes across human genes in future epidemiologic and pharmacogenetic studies.

## Introduction

Variation in allelic expression is very commonly observed in the human genome [1], [2] and, rather than alteration in protein products, may account for a significant fraction of inter-individual variation [3]. Therefore, identification of such variation is a major step toward understanding the differential predisposition to common diseases and variation in drug responses among individuals and ethnic populations. For example, slight changes in allelic expression of the tumor suppressor gene, *APC*, can affect predisposition to tumorigenesis [4]. Also, as recently illustrated, *VKORC1* gene expression influences the warfarin maintenance dose [5]. In recent years, studies searching for association between genetic markers and quantitative gene expression profiling, referred to as genetical genomics [6], have been conducted in model organisms and humans (reviewed in [7], [8]). Loci associated with the variation of gene expression, described as expression quantitative trait loci (eQTL), have been identified both *in cis* and *in trans* for many genes.

**Figure 1:**
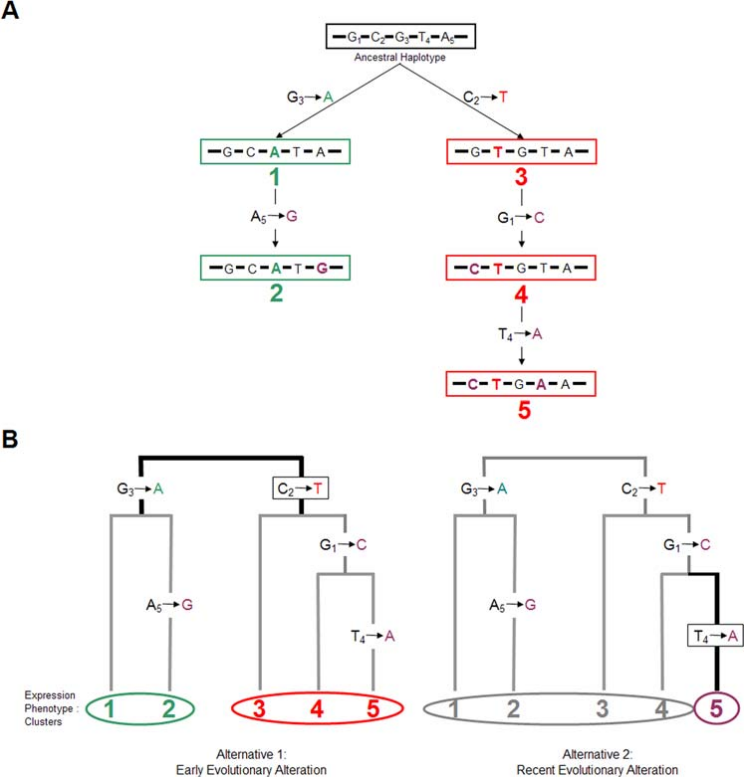
Ancestry-based haplotype clusters and the association with expression phenotypes. (A) In this hypothetical example, five extant haplotypes are observed (1–5) within a chromosome segment showing strong LD (low recombination rate). These haplotypes are derived through five mutation steps (resulting in five SNPs in current populations) from the inferred ancestral sequence (boxed in black) and can be grouped into two major haplotype clusters (boxed in green and red). Separating the ancestry-based haplotype clusters are earlier mutation steps (G_3_ → A; C_2_ → T). Alleles of these SNPs can be applied for “tagging” the clusters (typed in green and red). Currently, ancestry is commonly inferred by either the allele frequencies of SNPs or the corresponding nucleotides in non-human primate species. When the frequency of SNP alleles is applied (preferably using those of African populations), the haplotype clusters are referred to as “haplotype frameworks” [9]. The SNPs tagging the frameworks are termed “framework SNPs” or “fmSNPs”. (B) Tree structure of the five extant haplotypes and the expression phenotype clusters. Given a simple hypothesis that an historical mutation creates a variant altering the expression phenotype (resulting either enhancing or suppressing expression), two alternative schemes of resulting phenotype clusters associated with the variant are illustrated. The left panel exemplifies an evolutionarily earlier expression alteration caused by a mutation tagging the ancestry-based haplotype clusters, and the right panel demonstrates the alteration caused by a more recent mutation (with the mutations boxed and the resulting expression phenotype clusters circled).

Following an assessment of common underlying haplotype patterns across ethnic populations, we previously reported the observation that pairwise linkage disequilibrium (LD), based on the commonly used correlation coefficient, r^2^, between single nucleotide polymorphisms (SNPs) selected from populations having African ancestry shows strong conservation across other non-African populations, but not vice versa [9]. This observation is likely the consequence of a major population bottleneck out of Africa. Using these LD-selected SNPs, we demonstrated a defined SNP haplotype structure that is highly conserved across all ethnic populations. Hence, a set of globally-applicable tagging SNPs could be feasibly defined. Two recent studies investigating haplotype/LD variation and the transferability of tagging SNPs across global populations have provided strong support for our observation [10], [11]. The conserved common haplotypes we defined clustered into a simple evolutionary structure of up to three “haplotype frameworks”. SNPs tagging such haplotype frameworks (fmSNPs) could generally be identified within defined LD blocks as the ones having the highest allele frequencies in African-ancestry populations. These allele-frequency-derived, ethnically-conserved frameworks were likely the ancestral haplotype backgrounds upon which more recent mutations have been superimposed. Interestingly, our preliminary analysis suggested that a significant portion of reported variants strongly associated with *cis*-regulation tagged these globally-conserved haplotype frameworks [9]. A conceptual illustration of ancestry-based haplotype clusters and the association with expression phenotypes is presented in Figure 1.

In this report, we expanded the investigation of the relationship between *cis*-regulatory expression phenotypes and the SNPs tagging local haplotype frameworks (fmSNPs) by collecting and examining genes from major studies showing strong *cis*-regulatory association. We first delineated haplotype frameworks based on the high-density HapMap Phase II genotype data as described previously [9], followed by phylogenetic analysis among current major haplotypes and the corresponding haplotypes derived from chimpanzee reference sequences. We then measured the association between LD-derived tagging SNPs with expression phenotypes. As a consequence of this analysis, we observed significant correlation between SNPs showing the strongest association and SNPs tagging the major lineages directly separated from ancestry, inferred either from the frequency-derived haplotype frameworks (fmSNPs) or the chimpanzee reference sequences. We discuss the evolutionary implications of these findings for the origin and maintenance of expression variants in human populations, as well as for further genetic epidemiologic and pharmacogenetic studies.

## Results

To investigate the relationship between *cis*-regulatory expression phenotypes and the SNPs tagging local haplotype frameworks (fmSNPs), we analyzed a total of 26 genes (Table 1) showing stringently determined *cis*-association with expression phenotypes from five major studies (Morley et al. [12], Cheung et al. [13], Pastinen et al. [14], Deutsch et al. [15], Stranger et al. [16], reviewed by Pastinen et al. [17]). These studies were all conducted with lymphoblastoid cell lines of CEPH or HapMap CEU samples (Utah residents with ancestry from northern and western Europe), but using an earlier release (phase I) of HapMap genotype data having lower SNP density and employing different expression platforms. We first downloaded HapMap genotype data (Phase II; release 21) encompassing the gene pre-mRNA transcript and at least 10 kb upstream and downstream from initiation and termination sites, where predicted *cis*-regulatory modules (clusters of transcription factor binding sites) are most enriched [18]. We then constructed the local haplotype framework structure as previously described [9]. We used the originally-reported peak SNPs (SNPs showing strongest association) described in the above studies to serve as the lower-density screens (HapMap Phase I data) and then measured the association between gene expression and all tagging SNPs – this time taking advantage of the high-density HapMap Phase II data – within the block containing the reported peak SNPs. We applied public HapMap expression data across three major populations (GSE2552 [13] and GSE5859 [19], based on the Affymetrix platform, and GSE6536 [20], based on the Illumina platform).

**Table 1:** Correlation between SNPs showing strongest association with *cis*-regulation and SNPs tagging haplotype frameworks.

Gene		Genomic region investigated^1^	Tagging SNP showing strongest association with *cis*-regulation					
			Reference number	Allele frequency^2^ in CEU/ YRI/ CHB+JPT	Relative position to gene^3^	Tagging frequency-derived haplotype frameworks	Tagging lineages derived from chimpanzee reference	Nominal p-value significant across multiple populations^4^
***Class I: SNPs showing strongest association with cis-regulation tag frequency-derived haplotype frameworks***								
1	HSD17B12^5^^,^ 6	chr11:43648880..43844743; 195.9 kb	rs10838162	26%/41%/20%	intragenic	**Yes**	**Yes**	✓
2	IRF5^5^^,^ 6	chr7:128161944..128194036; 32.1 kb	rs2280714	42%/44%/47%	dn 4.6 kb^10^	**Yes**	**Yes**	✓
3	CD151^7^	chr11:812985..838833; 25.9 kb	rs4075289	26%/37%/5%	up 2.3 kb^10^	**Yes**	**Yes**	✓
4	CCT8^8^^,^ 9	chr21:29340517..29377880; 37.4 kb	rs965951	14%/29%/12%	intragenic	**Yes**	**Yes**	✓
5	PPAT^5^^,^ 6	chr4:57090458..57152772; 62.3 kb	rs9683679	29%/36%/29%	intragenic	**Yes**	**Yes**	✓
6	LOC388796^6^	chr20:36472655..36507865; 35.2 kb	rs3752278	9%/48%/13%	intragenic	**Yes**	**Yes**	✓
7	TMEM8^9^	chr16:351860..381907; 30.1 kb	rs3743888	39%/29%/62%	intragenic	**Yes**	**Yes**	✓
8	CTBP1^5^	chr4:1185057..1242737; 57.7 kb	rs3755920	46%/34%/70%	up 0.7 kb^10^	**Yes**	**Yes**	✓
9	ATF5^7^	chr19:55114271..55139002; 24.73 kb	rs3826777	36%/18%/44%	up 1.1 kb^10^	**Yes**	**Yes**	✓
10	ARTS-1^7^	chr5:96112276..96179397; 67.1 kb	rs30187	30%/39%/45%	intragenic	**Yes**	**Yes**	
11	IL16^5^	chr15:79252254..79402155; 149.9 kb	rs11638444	25%/43%/2%	intragenic	**Yes**	**Yes**	
12	CTSH^5^^,^ 6	chr15:76991161..77034474; 43.3 kb	rs1036938	29%/86%/87%	intragenic	**Yes**	No	✓
13	CHI3L2^5^^,^ 6	chr1:111472322..111508101; 35.8 kb	rs12048900	37%/27%/13%	up 4.2 kb^10^	**Yes**	No	✓
14	VAMP8^5^	chr2:85706374..85730810; 24.4 kb	rs3731828	38%/42%/33%	intragenic	**Yes**	No	✓
***Class II: SNPs showing strongest association with cis-regulation only tag lineages derived from chimp reference***								
1	BTN3A2^7^	chr6:26453120..26496524; 43.4 kb	rs9393713	13%/3%/11%	intragenic	No	**Yes**	✓
2	SERPINB10^9^	chr18:59723724..59763455; 39.7 kb	rs8085490	21%/81%/42%	intragenic	No	**Yes**	✓
3	LRAP^5^^,^ 6	chr5:96231023..96319053; 88.0 kb	rs2247650	49%/59%/56%	intragenic	No	**Yes**	✓
4	CAV2^9^	chr7:115669574..115742544; 73.0 kb	rs17138767	10%/1%/20%	up 1.8 kb^11^	No	**Yes**	✓
5	PAX8^7^	chr2:113679805..113762727; 82.9 kb	rs11123170	39%/28%/32%	intragenic	No	**Yes**	✓
6	CAT^7^	chr11:34407053..34460178; 53.1 kb	rs10836244	13%/13%/54%	intragenic	No	**Yes**	✓
7	OAS1^7^	chr12:111797458..111830430; 33 kb	rs1859336	40%/0%/27%	dn 9.6 kb^10^	No	**Yes**	
***Other:***								
1	RPS26^5^^,^ 6	chr12:54711952..54783960; 72.0 kb	rs11171739	38%/84%/27%	dn 32.6 kb^10^	No^13^	No	✓
2	CPNE1^5^^,^ 6 ^,^ 9	chr20:33667381..33726261; 58.9 kb	rs12480408	10%/8%/7%	intragenic	No^13^	No	✓
3	CSTB^5^^,^ 6 ^,^ 9	chr21:44008259..44068882; 60.6 kb	rs880987	18%/1%/48%	up 28.2 kb^12^	No^13^	No	✓
4	RAB7L1^7^	chr1:202397009..202475780; 78.8 kb	rs951366	42%/16%/40%	dn 52.3 kb^10^	No^13^	No	✓
5	SFRS6^9^	chr20:41509931..41558894; 49.0 kb	rs8124813	33%/1%/16%	dn 13.2 kb^10^	No^13^	No	

1 Including 10 kb upstream/downstream sequences of initiation/termination sites or an extended area to cover local LD block. Based on HapMap Phase II data release #21 in July 2006 and NCBI B35 assembly.

2 Frequency of rare allele derived in HapMap CEU Population versus the frequency of the same allele in YRI or CHB+JPT. CEU: CEPH (Utah residents with ancestry from northern and western Europe); YRI: Yoruba in Ibadan, Nigeria; CHB: Han Chinese in Beijing, China; JPT: Japanese in Tokyo, Japan.

3 Relative position (upstream; up / downstream; dn) to initiation/termination sites.

4 Based on public data from GSE 6536 (Illumina platform) or GSE 2552 / GSE 5859 (Affymetrix platform).

5 Reported in Morley et al., *Nature* 430, 743–7 (2004).

6 Reported in Cheung et al., *Nature* 437, 1365–9 (2005).

7 Reported in Pastinen et al., *Hum. Mol. Genet.* 14, 3963–71 (2005). Association data provided online from authors' website.

8 Reported in Deutsch et al., *Hum. Mol. Genet.* 14, 3741–9 (2005).

9 Reported in Stranger et al., *PLoS Genet.* 1, e78 (2005). Association data provided by the authors.

10 The LD block extends into intragenic region.

11 The LD block extends to upstream 0.8 kb.

12 The LD block extends to upstream 2.3 kb.

13 Haplotype framework-tagging SNP shows significant association in at least one population measured by either platform.

**Figure 2:**
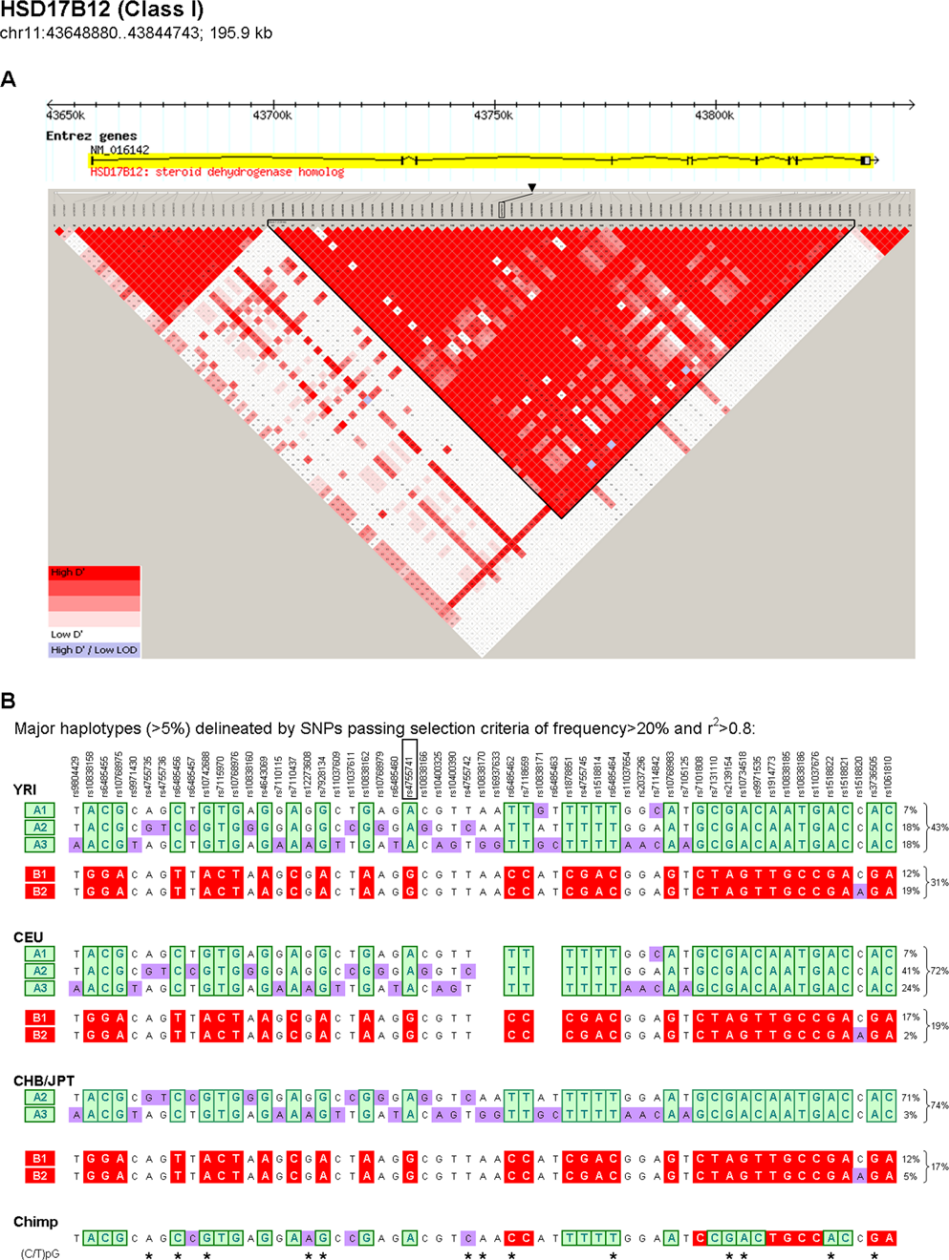
Delineation of underlying haplotype framework structure encompassing the *HSD17B12* gene (Class I *cis*-regulatory gene). (A) Diagram depicting the *HSD17B12* gene and its chromosomal position (reproduced from the HapMap graphical browser), aligned with the local LD structure determined in YRI (output from the Haploview program) using LD-selected SNPs. For simplifying this presentation, we focused on common SNPs (frequency >0.2) in either the HapMap YRI or CEU populations. Pairwise calculation of standardized LD, r^2^, was first determined using YRI data. SNPs in strong LD (r^2^>0.8) with at least one other SNP and also exhibiting conserved LD in CEU and CHB/JPT were selected for the LD plot and haplotype analyses. The original SNP reported to show the strongest association with expression (peak SNP) is marked with a solid black triangle at its physical position and mapped to its corresponding position in the LD plot. The LD block containing the peak SNP is surrounded with black lines. (B) Haplotype frameworks within the block containing the peak SNP. The major haplotypes (>5% in either population) and their population frequencies were inferred using the Haploview program. Five major haplotypes in the YRI population clustered into two haplotype frameworks (A and B) that can be tagged by a set of SNPs (fmSNPs) in strong LD and having the highest allele frequency within the block. The common alleles of fmSNPs are colored green, and the rare alleles red. The rare alleles of other lower-frequency SNPs are colored purple. Comparison of major haplotypes delineated in CEU and CHB/JPT using the same sets of SNPs showed an identical haplotype structure with a different frequency distribution as shown to the right. (Four SNPs having no genotype information in CEU were left blank.) All SNP reference (rs) numbers are shown above, with the original reported peak SNP, rs4755741, outlined in black. The chimpanzee nucleotides corresponding to each SNP are shown below. The colors of SNP alleles used in CEU, CHB/JPT, and chimpanzee follow the convention defined in YRI. The stars below chimpanzee nucleotides indicate polymorphisms located at (C/T)pG positions on either strand.

A typical example, *HSD17B1*, is depicted in Figure 2, in which the intragenic SNP, rs4755741, was reported as the peak SNP [13]. To delineate the local haplotype framework structure from the YRI population and compare it to that of other major populations, we downloaded genotypes of HapMap SNPs encompassing a total of 195.9 kb, including 10 kb upstream of the transcription initiation site and 10 kb downstream of the termination site. We then selected SNPs in strong LD (r^2^>0.8) against at least one other SNP conserved across populations and inferred major haplotypes (>5%; see Panel B) within the block containing the peak SNP (the labeled triangular area in the LD plot in Panel A). To simplify the presentation in Figure 2, we show only SNPs with rare allele frequencies greater than 20% in either population (additional SNPs do not alter the primary result we obtained). These major haplotypes clustered into two frameworks, A and B, tagged by a set of fmSNPs having the highest allele frequency within the block (common and rare alleles are colored in green and red, respectively). Major haplotypes within each framework can be further tagged by other SNPs having lower allele frequencies (rare alleles colored in purple). For this gene, as well as many others (genes 1 to 14 in Table 1; also see Supporting Information Figures S1 for detailed analyses), the fmSNPs showed the strongest association with the expression phenotype. We designated these genes (14 of 26) as class I in Table 1.

**Figure 3:**
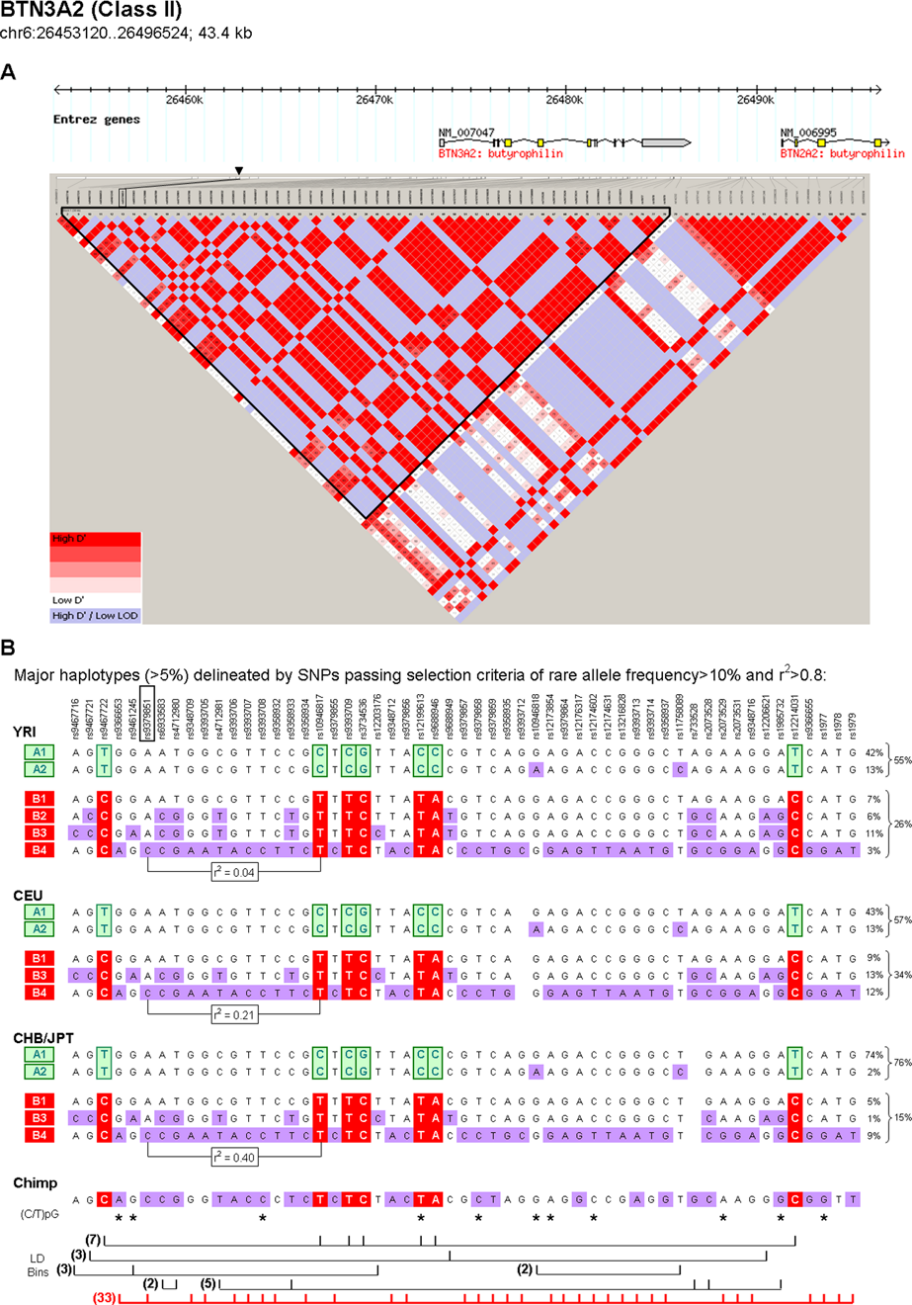
Delineation of underlying haplotype framework structure encompassing the *BTN3A2* gene (Class II *cis*-regulatory gene). (A) Diagram depicting the *BTN3A2* gene, its chromosomal position, and the local LD structure. This panel follows the convention in Figure 2 except that, for simplifying the presentation, we focused on common SNPs with frequency >0.1 in HapMap populations. (B) Haplotype frameworks within the block containing the peak SNP. This panel also follows the convention in Figure 2. The pairwise LD measure, r^2^, between the peak SNP and fmSNP is shown in all three populations. Sets of SNPs in strong LD, determined using YRI genotypes and based on the criterion of r^2^>0.8, are depicted at the bottom. The number of SNPs in each bin is shown to the left. The SNP set marked in red, containing an extraordinarily large number of SNPs relative to other bins (tagging haplotype B4 within the block), shows the strongest association with expression phenotypes.

In addition to the peak SNP-fmSNP correlation, we observed that a few SNPs showing the strongest association, despite having no correlation with fmSNPs, exhibited a unique characteristic: namely, that of being in strong LD against a relatively large number (the vast majority) of other SNPs within the LD block. As shown in Figure 3, at *BTN3A2*, using the same SNP selection criterion of pairwise LD (r^2^>0.8), the major haplotypes within the block were delineated (Panel B). The heritable, unidirectional allelic imbalance and the regulatory haplotype of this gene were also discussed in Pastinen et al. [21]. Although the reported peak SNP, rs9379851, was not in strong LD against frequency-derived fmSNPs (r^2^ = 0.04, 0.21, 0.40 in YRI, CEU, and CHB/JPT populations, respectively), it was highly correlated with many other SNPs tagging the haplotype B4 within the 24 kb LD block (3%, 12%, 9% frequency in YRI, CEU, and CHB/JPT, respectively).

To date, most genetical genomics studies are solely based on association tests between individual SNPs and expression phenotypes. One advantage of our haplotype-based approach is its capability of incorporating evolutionary analysis. Currently, there are two general approaches for inferring ancestry — one is based on the frequency of SNP alleles and the other on the comparison of corresponding nucleotides in species closely related to human beings, e.g., chimpanzees. Independent studies have reported that there was a general agreement between the two approaches [22], [23]. The more common human allele generally matches the corresponding nucleotide in the chimpanzee genome (76% concordance as reported in Hacia et al. [22]). Given the conservation of the haplotype frameworks defined by fmSNPs across other out-of-Africa populations [9], these frameworks are likely haplotype backgrounds upon which more recent mutations having lower allele frequencies have been superimposed. Since all genes selected for this study are reported *cis*-regulatory genes, we considered whether our observed correlation between fmSNPs and SNPs showing the strongest association with expression differences was a consequence of selection in earlier human history and whether genes behaving like *BTN3A2* were under more recent selection in the African population, resulting in population-specific frequency distortion.

**Figure 4:**
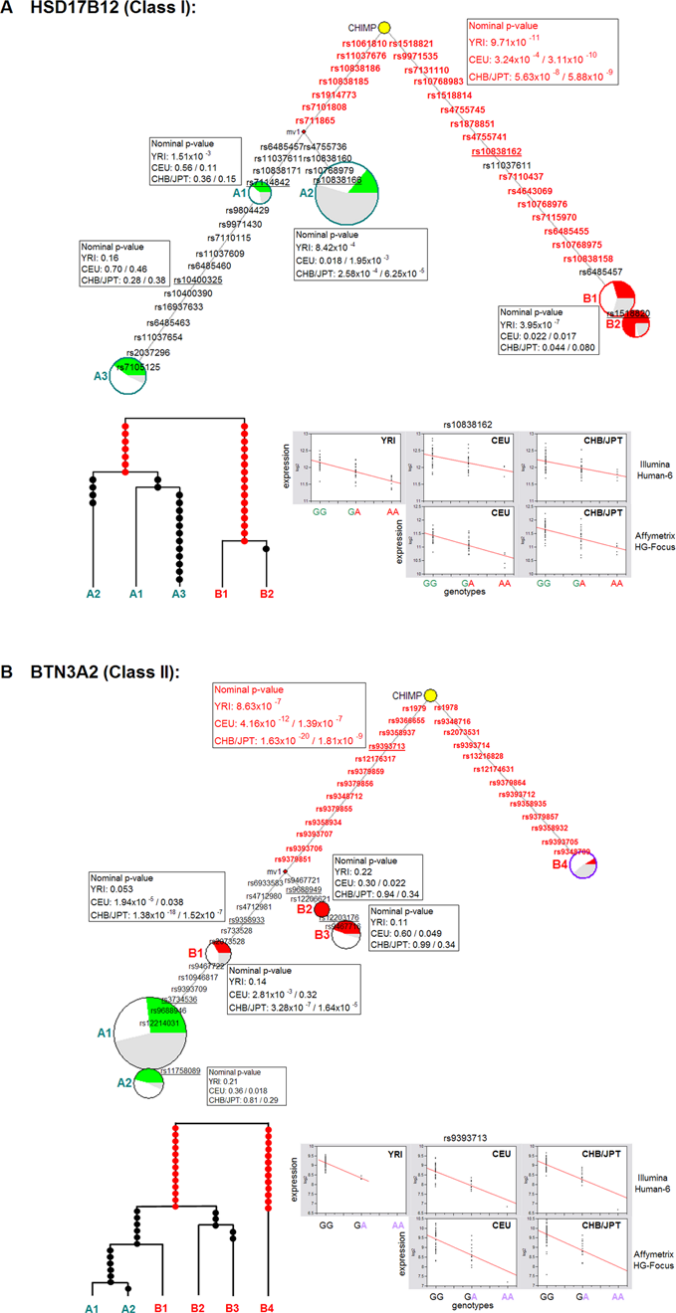
Phylogenetic relationships among current major haplotypes and the association to expression phenotypes. Median-joining (MJ) network analysis was conducted using the Network program for *HSD17B12* (Class I; shown in panel A) and *BTN3A2* (Class II; shown in panel B). The major haplotypes in HapMap populations shown in Figure 2 and 3 were entered, using their population haplotype frequencies, along with the corresponding chimpanzee haplotype. The SNPs located at (C/T)pG positions on either strand (marked with stars in panel B of the previous figures) were generally excluded from this analysis because of their potentially high mutation rates. Each haplotype is represented by a circle. The area of each circle, except for the chimpanzee reference (colored yellow), reflects the observed frequency of each haplotype in the total dataset (YRI, CEU, CHB/JPT). The portion of YRI, CEU, and CHB/JPT chromosomes in each circle is denoted with green/red, white, and grey colors, respectively. The colors of haplotype frameworks A and B follow the green and red convention in previous figures. The length of lines between any two haplotype nodes is proportional to the number of mutation steps. The rs numbers of SNPs are labeled along the lines. The median vector (mv) is shown as a small circle and can be interpreted as possibly extant unsampled haplotypes. For each set of SNPs in strong LD (generally marking the branches in the lineage), a SNP with the most complete genotypes (underlined) was chosen for testing association. The nominal p-values for these SNPs in each major population, based on expression data sets GSE6536 (Illumina platform) and GSE2552/GSE5859 (Affymetrix platform), are shown in order. The coalescent-based maximum likelihood tree structure and the regression of expression phenotypes are plotted at the bottom of each panel. The set of SNPs in LD showing the strongest association with expression phenotypes is typed in red in the phylogenetic network and shown as red dots in the genealogical tree.

We subsequently conducted evolutionary analysis of the common haplotypes across all 26 genes (Supporting Information Figures S1). For all the SNPs employed in our haplotype construction, we mapped the corresponding chimpanzee nucleotides using chimpanzee reference sequences, followed by median-joining (MJ) network analysis to derive phylogenetic relationships among all major haplotypes. As shown in Figure 4A, at *HSD17B12*, the two frequency-derived haplotype frameworks (A and B) were separated directly from the ancestral haplotype. In addition, we performed coalescent-based likelihood analysis to draw the maximum likelihood genealogical relationships among the common haplotypes. The result also supported the hypothesis that underlying these haplotype frameworks were older mutations closer to the root of the gene tree. Thus, the differential expression pattern of these haplotypes was likely to have appeared early in human evolutionary history.

For genes showing the features exemplified by *BTN3A2*, we observed a weaker correlation between the more common allele and the chimpanzee nucleotide, i.e., a more significant proportion of the rare alleles matched the ancestral nucleotides. Phylogenetic analysis, typified by the results of Figure 4B, suggested that the ancestral haplotype was between the haplotype, B4, carrying a relatively large number of tagging SNPs in strong LD, including the peak SNP, and the rest of the B haplotypes. Similarly, the coalescent-based maximum likelihood tree structure also suggested that the accumulation of such a long stretch of B4-tagging SNPs likely occurred early in the tree. Hence, for such genes, the differential expression pattern also evolved early, except that the frequency of the *cis*-regulatory haplotype showing differential expression was often lower in current populations having African ancestry, presumably a consequence of more recent population-specific selection. We designated these genes (following only the chimpanzee-inferred ancestry) as Class II (7 genes out of 26) in Table I. Five of them showed the characteristic of carrying a relatively large number of tagging SNPs in strong LD (*BTN3A2*, *SERPINB10*, *LRAP*, *CAV2*, *OAS1*).

Since the majority of our sampled genes exhibited the same evolutionarily conserved feature, we next asked whether their *cis*-regulatory phenotypes were, as expected, also conserved across populations. Based on one set of expression data in YRI (GSE6536, Illumina platform) and two sets data in CHB/JPT (GSE5859, Affymetrix platform; GSE6536, Illumina platform), we tested the association for all tagging SNPs (Figure 4 and Supporting Information Figures S1). Only three genes in Class I and II did not show significant association in at least one other population (Table 1). In addition, for all genes showing significant *cis*-association across multiple ethnic populations, the direction of allelic effect on expression was always consistent, strongly supporting the hypothesis that the *cis*-regulation was derived early and still being maintained in current populations.

Overall, among the 26 *cis*-regulatory loci we analyzed, frequency-derived fmSNPs showed strongest association with expression phenotypes for 14 genes (Class I), 11 of which also demonstrated the chimpanzee-inferred ancestry. Seven genes (Class II) followed only the chimpanzee-inferred ancestry, but not the frequency-derived haplotype frameworks. A total of 80% (21/26) of genes followed either frequency- or chimpanzee- inferred ancestry. To determine if this distribution of *cis*-regulatory loci could be the result of chance, we performed simulations using LD-selected common SNPs within the analyzed LD blocks of the same 26 genes. Assuming that every SNP had the same probability to be the *cis*-regulatory variant, our simulations, under a completely random-occurrence scenario, resulted in an average of only 13 genes showing an association of a *cis*-regulatory variant with the SNPs tagging the major branches separated from ancestry. When compared to the total of 21 genes actually observed, our simulation resulted in a significant deviation (p = 10^−6^). Therefore, we rejected the null hypothesis of randomness. We concluded that, in the 26 genes, there was a higher probability of SNPs tagging the major lineages separated from ancestry to be *cis*-regulatory variants. Also of note, for the five genes showing no correlation with either ancestry inference, the frequency-derived fmSNPs of these five genes all showed individually significant association in at least one population, measured by at least one platform.

## Discussion

Earlier linkage studies have shown that quantitative gene expression levels are significantly heritable [12], [24]. Although both *cis*- and *trans*-linkages have been detected, one interesting observation has been the enrichment of *cis*-linkages among the strongest signals, a phenomenon also observed in mice and rats [25]–[27]. Recent genetical genomics studies based on whole genome association tests have also revealed that a majority of signals for differential expression are *cis*-acting [13], [16]. Overall, current data suggest that *cis*-regulatory effects are more consistent and larger. In contrast, *trans*-acting signals are more modestly significant and often are not replicated (reviewed in [7], [17]).

Since our current knowledge of *trans*-acting regulation may still be insufficient for comprehensive association studies [17], an adequate approach at this stage would be to focus on the identification of *cis*-regulatory genes that are heritable as a monogenic trait. Currently, most genetical genomics studies searching for *cis*-regulatory genes are based on association tests between individual SNPs and expression phenotypes. However, while the SNP density employed in the commercially available, high-throughput platforms keeps growing, the major trade-off is true associations failing to pass the stringent statistical correction for multiple testing. Our analysis indicates that, since the vast majority of true *cis*-regulatory genes carry evolutionarily common signatures, the use of such signatures (fmSNPs for class I genes and subhaplotypes with many SNPs in high LD for class II genes) should provide more effective identification of true positives. Also, since recent major studies have only focused on a limited number of expressed genes in lymphoblastoid cell lines, learning the common genetic characteristics of identified *cis*-regulatory genes from these studies should help future identification of other globally-conserved *cis*-regulatory genes across different tissues.

Genetical genomics studies, often based on different platforms with different experimental designs, have in the past shown poor correlation between studies [17]. Examples are given at *LRAP* and *SFRS6* (Supporting Information Figures S1), where an apparent discrepancy across the two major commercial platforms, Illumina and Affymetrix, is shown. In the case of *SFRS6*, different 50mer probes used in the Illumina platform also produced a discrepancy, probably a consequence of different probes recognizing alternative transcripts. Other questions regarding statistical analysis and cell line variability have also arisen, leading to warnings to interpret results with caution [7], [28], [29]. We would like to note, however, that our observations were based on a collection of *cis*-regulatory genes from independent studies, conducted in different laboratories using different approaches, but confirmed using an independent dataset with a larger sample size. Although the number of genes we collected is limited in this study, we nonetheless observed common genetic features of these *cis*-regulatory genes that could be applied to a significant fraction of genes analyzed (21/26 in which the reported associations could be replicated). While it is possible that our observation was only a result of enrichment of a specific profile of *cis*-regulation using the top association hits from different studies, other examples fitting our observation have independently appeared in recent literature, for example, the clustering of *VKORC1* and *NPY* haplotypes based on their expression phenotypes and their correlation to drug and stress response [5], [30]. This suggests that these features may be a general and powerful means of discovering evolutionarily-conserved variants of gene expression. Since the variants were generally common in current populations, they will likely prove useful for validating expression differences across multiple tissues, populations and enhancing our understanding of the differential predisposition to common diseases and variation in drug responses in different ethnic groups.

Recent surveys have shown that many gene coding regions in the human genome do not show an excess of low-frequency alleles, suggesting that balancing selection might be more common than previously thought (reviewed by Bamshad et al. [31]). Our analyses also revealed that haplotypes in current populations carrying high- and low-expression phenotypes were nearly exclusively evolved early in human evolutionary history (Figure 4), likely as a consequence of balancing selection. Therefore, disease gene variation taking the form of *cis*-acting eQTLs may have a narrower allelic spectrum toward high population frequencies, as predicted by the common diseases/common variant (CDCV) model that genetic risk of common diseases is often conferred by alleles having relatively high frequencies [32].

## Methods

### *cis*-regulatory genes included in this study

As shown in Supporting Information Table 1, a total of 44 genes showing stringently determined *cis*-association with expression phenotypes were initially collected from five major studies [12]–[16]. These studies were all conducted with lymphoblastoid cell lines of CEPH or HapMap CEU samples (Utah residents with ancestry from northern and western Europe) using an earlier release (phase I) of HapMap genotype data, but employing different expression platforms. Although the majority of these genes demonstrated prior positive results for linkage or allelic imbalance (AI) assays, we added a further validation step by confirming the *cis*-association using an independent dataset having a relatively large sample size [33] (GSE8052; Affymetrix platform; 400 UK samples). Thirty of the 44 genes passed the genome-wide significance threshold (a LOD score of 6.076, corresponding to a false discovery rate of 0.05, as listed in supplementary table 1 in Dixon et al. [33]). Of the thirty genes, four genes were excluded from our analysis (*GSTM1* and *GSTM2*: known region of structure variation [deletion]; *PSPHL*: probe 205048_s_at mapped to a region having no annotated gene in the b35 assembly; *POMZP3*: HapMap SNP density too low for our haplotype analysis). Overall, we included 26 genes for our haplotype and *cis*-association analysis listed in Table 1.

### SNP selection for delineating haplotype framework structure

SNP data from HapMap release 21/Phase II in July, 2006, based on NCBI b35 assembly and dbSNP b125, were downloaded using the graphical browser provided by the International HapMap Project (http://www.hapmap.org/). For regions encompassing at least 10 kb upstream and downstream from initiation and termination sites of the pre-mRNA transcript, genotypes (forward strand) of 60 YRI (Yorubans of Ibadan, Nigeria), 60 CEU (Utah residents with ancestry from northern and western Europe) individuals (parents of family trios) and 90 CHB/JPT (Han Chinese in Beijing, China, and Japanese in Tokyo, Japan) were employed for LD-based SNP selection and local haplotype framework analyses. For delineating major haplotypes with frequencies greater than 5% in current populations, SNPs having rare allele frequencies greater than 5% in any population were screened first (unless otherwise noted in the figures), followed by selection using the pairwise LD measure [34], r^2^, for those in strong LD against at least one other SNP (based on the criterion of r^2^>0.8). SNPs showing conserved LD behavior across populations were employed in haplotype construction, as described in our previous publication [9].

### Delineation of LD and haplotype framework structure

For each analyzed region, the LD plotting, haplotype block partitioning, and the delineation and population frequency estimation of major haplotypes were performed by the HAPLOVIEW program, version 3.32 (http://www.broad.mit.edu/mpg/haploview) [35]. The haplotype block partitioning was generally determined with YRI data using one of the three methods (confidence intervals, four gamete rule, solid spine of LD) incorporated into the HAPLOVIEW program, depending on which covered the most extensive area containing the peak SNP. For some genes, we covered more extensive regions to increase informativeness. The haplotype frameworks were clustered based on YRI allele frequencies, as described in our previous publication [9].

### Genealogical analysis

Phylogenetic relationships among major haplotypes were analyzed by the Median Joining (MJ) network algorithm packaged in the NETWORK program, version 4.201 (http://www.fluxus-engineering.com/sharenet.htm) [36]. The major haplotypes in either population, along with chimpanzee haplotypes, were entered with their population haplotype frequencies. The chimpanzee haplotypes were derived using corresponding nucleotides in the chimpanzee reference sequences, retrieved using the UCSC genome browser (http://genome.ucsc.edu/) [37]. SNPs located at (C/T)pG positions on either strand, because of their higher mutation rate, were generally excluded from this analysis.

Coalescent-based genealogical analysis was performed by the GENETREE program version 9.0 (http://www.stats.ox.ac.uk/griff/software.html) [38]. It applies the Markov chain simulation to perform likelihood estimates of tree probabilities under the infinite site model. The major haplotypes in the three populations (denoted as subpopulations) at *HSD17B12* and *BTN3A2* (shown in Figure 4) were entered using their population haplotype frequencies. The chimpanzee corresponding alleles of the polymorphic sites were designated as ancestral alleles.

### Association analysis

LD-derived SNP bins were defined from SNPs within the block containing the originally reported peak SNPs using the TAGGER program (“tagger pairwise” option) incorporated into the HapMap graphical browser [39]. For each bin (generally marking the branches in the genealogical analysis), a tagging SNP was selected based on the completeness of genotypes across the three populations for testing association. Association analysis between each tagging SNP and two sets of HapMap expression data, based on two (Affymetrix and Illumina) platforms and across three HapMap populations, (GEO accession number GSE2552 [13], GSE5859 [19], and GSE6536 [20]), was conducted by following the regression methods described in Cheung et al. [13] (discussed in [40]). The nominal p-value of each tagging SNP was used for the determination of SNPs showing strongest association. The value of 0.05 was used as our cutoff for statistical significance.

### Monte-Carlo-based simulation

We tested the hypothesis whether *cis*-regulatory SNPs were randomly distributed along the genealogical tree versus an alternative that there was an enrichment or selection effect among the 26 genes in Table 1. We performed a Monte-Carlo-based simulation under the assumption that every common SNP has the same probability to be the *cis*-regulatory variant. For each gene, we randomly selected an LD-selected common SNP (as shown in all figures) under a binomial distribution. The number of trials was the count of common SNPs and the probability of a *cis*-regulatory variant was the number of SNPs tagging the major lineages separated from ancestry divided by the total number of LD-selected common SNPs across the investigated LD blocks of all genes. Our test statistic compared the average number of genes found in a series of 1,000,000 simulations, versus the observed 21 genes.

## Supporting Information

Supporting Information Figures S1 (7.15 MB PDF)

## References

[1] Lo HS, Wang Z, Hu Y, Yang HH, Gere S (2003). Allelic variation in gene expression is common in the human genome.. Genome Res.

[2] Pant PV, Tao H, Beilharz EJ, Ballinger DG, Cox DR (2006). Analysis of allelic differential expression in human white blood cells.. Genome Res.

[3] Pastinen T, Hudson TJ (2004). Cis-acting regulatory variation in the human genome.. Science.

[4] Yan H, Dobbie Z, Gruber SB, Markowitz S, Romans K (2002). Small changes in expression affect predisposition to tumorigenesis.. Nat Genet.

[5] Rieder MJ, Reiner AP, Gage BF, Nickerson DA, Eby CS (2005). Effect of VKORC1 haplotypes on transcriptional regulation and warfarin dose.. N Engl J Med.

[6] Jansen RC, Nap JP (2001). Genetical genomics: the added value from segregation.. Trends Genet.

[7] de Koning DJ, Haley CS (2005). Genetical genomics in humans and model organisms.. Trends Genet.

[8] Gibson G, Weir B (2005). The quantitative genetics of transcription.. Trends Genet.

[9] Ouyang C, Krontiris TG (2006). Identification and functional significance of SNPs underlying conserved haplotype frameworks across ethnic populations.. Pharmacogenet Genomics.

[10] Conrad DF, Jakobsson M, Coop G, Wen X, Wall JD (2006). A worldwide survey of haplotype variation and linkage disequilibrium in the human genome.. Nat Genet.

[11] de Bakker PI, Burtt NP, Graham RR, Guiducci C, Yelensky R (2006). Transferability of tag SNPs in genetic association studies in multiple populations.. Nat Genet.

[12] Morley M, Molony CM, Weber TM, Devlin JL, Ewens KG (2004). Genetic analysis of genome-wide variation in human gene expression.. Nature.

[13] Cheung VG, Spielman RS, Ewens KG, Weber TM, Morley M (2005). Mapping determinants of human gene expression by regional and genome-wide association.. Nature.

[14] Pastinen T, Ge B, Gurd S, Gaudin T, Dore C (2005). Mapping common regulatory variants to human haplotypes.. Hum Mol Genet.

[15] Deutsch S, Lyle R, Dermitzakis ET, Attar H, Subrahmanyan L (2005). Gene expression variation and expression quantitative trait mapping of human chromosome 21 genes.. Hum Mol Genet.

[16] Stranger BE, Forrest MS, Clark AG, Minichiello MJ, Deutsch S (2005). Genome-wide associations of gene expression variation in humans.. PLoS Genet.

[17] Pastinen T, Ge B, Hudson TJ (2006). Influence of human genome polymorphism on gene expression.. Hum Mol Genet.

[18] Blanchette M, Bataille AR, Chen X, Poitras C, Laganiere J (2006). Genome-wide computational prediction of transcriptional regulatory modules reveals new insights into human gene expression.. Genome Res.

[19] Spielman RS, Bastone LA, Burdick JT, Morley M, Ewens WJ (2007). Common genetic variants account for differences in gene expression among ethnic groups.. Nat Genet.

[20] Stranger BE, Nica AC, Forrest MS, Dimas A, Bird CP (2007). Population genomics of human gene expression.. Nat Genet.

[21] Pastinen T, Sladek R, Gurd S, Sammak A, Ge B (2004). A survey of genetic and epigenetic variation affecting human gene expression.. Physiol Genomics.

[22] Hacia JG, Fan JB, Ryder O, Jin L, Edgemon K (1999). Determination of ancestral alleles for human single-nucleotide polymorphisms using high-density oligonucleotide arrays.. Nat Genet.

[23] Stephens JC, Schneider JA, Tanguay DA, Choi J, Acharya T (2001). Haplotype variation and linkage disequilibrium in 313 human genes.. Science.

[24] Monks SA, Leonardson A, Zhu H, Cundiff P, Pietrusiak P (2004). Genetic inheritance of gene expression in human cell lines.. Am J Hum Genet.

[25] Schadt EE, Monks SA, Drake TA, Lusis AJ, Che N (2003). Genetics of gene expression surveyed in maize, mouse and man.. Nature.

[26] Hubner N, Wallace CA, Zimdahl H, Petretto E, Schulz H (2005). Integrated transcriptional profiling and linkage analysis for identification of genes underlying disease.. Nat Genet.

[27] Doss S, Schadt EE, Drake TA, Lusis AJ (2005). Cis-acting expression quantitative trait loci in mice.. Genome Res.

[28] Williams RB, Cotsapas CJ, Cowley MJ, Chan E, Nott DJ (2006). Normalization procedures and detection of linkage signal in genetical-genomics experiments.. Nat Genet.

[29] Akey JM, Biswas S, Leek JT, Storey JD (2007). On the design and analysis of gene expression studies in human populations.. Nat Genet.

[30] Zhou Z, Zhu G, Hariri AR, Enoch MA, Scott D (2008). Genetic variation in human NPY expression affects stress response and emotion.. Nature.

[31] Bamshad M, Wooding SP (2003). Signatures of natural selection in the human genome.. Nat Rev Genet.

[32] Lander ES (1996). The new genomics: global views of biology.. Science.

[33] Dixon AL, Liang L, Moffatt MF, Chen W, Heath S (2007). A genome-wide association study of global gene expression.. Nat Genet.

[34] Devlin B, Risch N (1995). A comparison of linkage disequilibrium measures for fine-scale mapping.. Genomics.

[35] Barrett JC, Fry B, Maller J, Daly MJ (2005). Haploview: analysis and visualization of LD and haplotype maps.. Bioinformatics.

[36] Bandelt HJ, Forster P, Rohl A (1999). Median-joining networks for inferring intraspecific phylogenies.. Mol Biol Evol.

[37] Kent WJ, Sugnet CW, Furey TS, Roskin KM, Pringle TH (2002). The human genome browser at UCSC.. Genome Res.

[38] Griffiths RC, Tavare S (1994). Ancestral Inference in Population Genetics.. Stat Sci.

[39] de Bakker PI, Yelensky R, Pe'er I, Gabriel SB, Daly MJ (2005). Efficiency and power in genetic association studies.. Nat Genet.

[40] Spielman RS, Cheung VG (2007). Reply to “On the design and analysis of gene expression studies in human populations”.. Nat Genet.

