# Supplementary material for: Evolutionary Signatures of Common Human Cis-Regulatory Haplotypes
Source: PLoS One. 2008 Oct 10;3(10):e3362. doi: 10.1371/journal.pone.0003362 (PMC2557141; doi:10.1371/journal.pone.0003362)
Supplement: Supporting Information Figures S1 — (7.15 MB PDF) [file pone.0003362.s001.pdf]

Supporting Information

Evolutionary signatures of common human *cis*-regulatory haplotypes

Ching Ouyang, David D. Smith and Theodore G. Krontiris

This file includes:

Supplementary Table S1

Supplementary Figures S1 to S27

Supplementary Table 1: Genes showing significant *cis*-association from literature and the inclusion to our analysis.

Gene	Inclusion to our analysis (Table 1)	Validation of strong *cis*-association in GSE8052 (LOD > 6) [1,2]			
		SNP	probe ID	p-value	LOD
Cheung et al.[3] and Morley et al.[4] (18 genes showing significant association in genome-wide linkage and association studies)					
1 RPS26	Yes	rs1506440	217753_s_at	3.6×10^{-61}	59.13
2 LRAP(LOC64167)	Yes	rs2548225	219759_at	8.4×10^{-52}	49.79
3 AA827892(LOC388796)	Yes	rs788350	65588_at	4.4×10^{-50}	48.08
4 IRF5	Yes	rs2280714	239412_at	7.4×10^{-22}	35.91
5 CPNE1	Yes	rs11698601	206918_s_at	1.2×10^{-32}	30.75
6 CHI3L2	Yes	rs961364	213060_s_at	1.9×10^{-30}	28.57
7 CTBP1	Yes	rs4045131	213979_s_at	1×10^{-28}	26.85
8 HSD17B12	Yes	rs1061810	217869_at	4.6×10^{-26}	24.21
9 CTSH	Yes	rs4779112	202295_s_at	4.2×10^{-23}	21.28
10 PPAT	Yes	rs11133439	209433_s_at	1.1×10^{-22}	20.85
11 CSTB	Yes	rs1041456	201201_at	3.2×10^{-17}	15.46
12 VAMP8	Yes	rs3731828	202546_at	2.7×10^{-12}	10.62
13 IL16	Yes	rs4616256	209828_s_at	1.5×10^{-10}	8.91
14 PSPHL	No[8]	rs1113765	205048_s_at	1.4×10^{-24}	22.74
15 POMZP3	No[9]	rs804025	204148_s_at	3×10^{-13}	11.55
16 GSTM1	No[10]	rs11101992	215333_x_at	3.9×10^{-10}	8.51
17 GSTM2	No[10]	rs11101992	204418_x_at	3.4×10^{-10}	8.57
18 HLA-DRB2	No[11]	N/A	N/A	N/A	N/A
Stranger et al.[5] (10 genes showing significant association after Bonferroni and/or permutation-based correction in *cis*-regulatory regions (within 1 Mb))					
4 CPNE1	Yes	same as CPNE1 above			
5 CSTB	Yes	same as CSTB above			
19 SFRS6-002	Yes	rs8124813	206108_s_at	1.2×10^{-29}	27.75
20 CCT8	Yes	rs2245431	200873_s_at	9.8×10^{-23}	20.91
21 SERPINEB10	Yes	rs9967382	214539_at	1.9×10^{-22}	20.63
22 CAV2	Yes	rs2191498	203323_at	1.1×10^{-15}	13.97
23 TMEM8	Yes	rs540	222718_at	3.7×10^{-14}	12.45
24 HRMT1L1	No[11]	N/A	N/A	N/A	N/A
25 TTC3	No[11]	N/A	N/A	N/A	N/A
26 C21orf56	No[11]	N/A	N/A	N/A	N/A
Deutsch et al.[6] (1 gene validated for significant *cis*-association (100 kb surrounding the gene) after correction for multiple testing)					
20 CCT8	Yes	same as CCT8 above			
Pastinen et al.[7] (18 genes showing $P_{corrected}$ < 0.05 in AI association tests and reaching nominal significance in hypothesis driven total expression association tests)					
27 OAS1	Yes	rs10774671	202869_at	7×10^{-48}	45.89
28 ARTS-1	Yes	rs27434	209788_s_at	2.7×10^{-32}	30.39
29 PAX8	Yes	rs7589901	227474_at	3.6×10^{-31}	29.28
30 BTN3A2	Yes	rs9379859	209846_s_at	2.3×10^{-28}	26.49
31 CD151	Yes	rs1130678	204306_s_at	9.1×10^{-18}	16.00
33 CAT	Yes	rs2284365	201432_at	3.8×10^{-13}	11.45
34 RAB7L1	Yes	rs3761919	218700_s_at	1.8×10^{-9}	7.87
35 ATF5	Yes	rs3826777	204998_s_at	6.1×10^{-8}	6.37
32 KL	No[11]	N/A	N/A	N/A	N/A
36 EPHX2	No[11]	N/A	N/A	N/A	N/A
37 WRB	No[11]	N/A	N/A	N/A	N/A
38 GUCY1A3	No[11]	N/A	N/A	N/A	N/A
39 MTHFD2	No[11]	N/A	N/A	N/A	N/A
40 PISD	No[11]	N/A	N/A	N/A	N/A
41 FVT1	No[11]	N/A	N/A	N/A	N/A
42 DUSP4	No[11]	N/A	N/A	N/A	N/A
43 VDR	No[11]	N/A	N/A	N/A	N/A
44 CORO1C	No[11]	N/A	N/A	N/A	N/A

[1] Data from Supplementary Table 1 in Dixon et al., a list of associations between SNPs and probes having LOD scores > 6.
[2] Affymetrix expression platform (U133 Plus 2.0) and Illumina beadchip genotyping platform (Sentrix Human-1/HumanHap300) across 400 individuals.
[3] Cheung et al., *Nature* 437, 1365-9 (2005).
[4] Morley et al., *Nature* 430, 743-7 (2004).
[5] Stranger et al., *PLoS Genet.* 1, e78 (2005).
[6] Deutsch et al., *Hum. Mol. Genet.* 14, 3741-9 (2005).
[7] Pastinen et al., *Hum. Mol. Genet.* 14, 3963-71 (2005).
[8] Position of Affy probe (205048_s_at) mapped to a region having no annotated gene in B35 assembly. (There are two Affy probes for PSPH, 205048_s_at and 205194_at. The reported peak SNP rs6593279 shows significant association with 205048_s_at only.)
[9] Low HapMap genotyped SNP density for haplotype analysis.
[10] Peak SNP mapped to known region of structure variation (deletion), identified by fosmids mapping (UCSC genome browser custom track hg17/hg18).
[11] Either did not pass validation or no data for validation.

Supplementary Figures S1 to S26: Correlation between LD-selected tagging SNPs showing strongest association with *cis*-regulation and SNPs tagging lineages derived from ancestry. Ancestry was inferred either by frequency-based haplotype frameworks or the corresponding chimpanzee haplotype for all *cis*-regulatory genes listed in Table 1.

(A) Delineation of the LD/haplotype block structure surrounding the reported *cis*-regulatory genes. The diagrammatic presentation follows the convention shown in panel A of Figures 1 and 2.

(B) Delineation of conserved haplotype frameworks within the block containing the peak SNP. The diagrammatic presentation follows the convention shown in panel B of Figures 1 and 2. SNPs applied to phylogenetic analysis are numbered at the bottom for simplifying the presentation in panel C.

(C) Phylogenetic relationships among current major haplotypes and the association to expression phenotypes. The diagrammatic presentation follows the convention shown in Figure 3.

Supplementary Figure S27: Discrepancy of expression data resulting from different probe designs or platforms at *LRAP* (Panel A) and *SFRS6* (Panel B). The physical map of genes and alternative forms of gene structure were reproduced from the UCSC genome browser. The relative positions of probes applied by Illumina (50mers) and Affymetrix platforms are shown below the display of alternative transcripts. Regression plots based on different probes/platforms were performed using expression data in the CEU population.

HSD17B12

A.

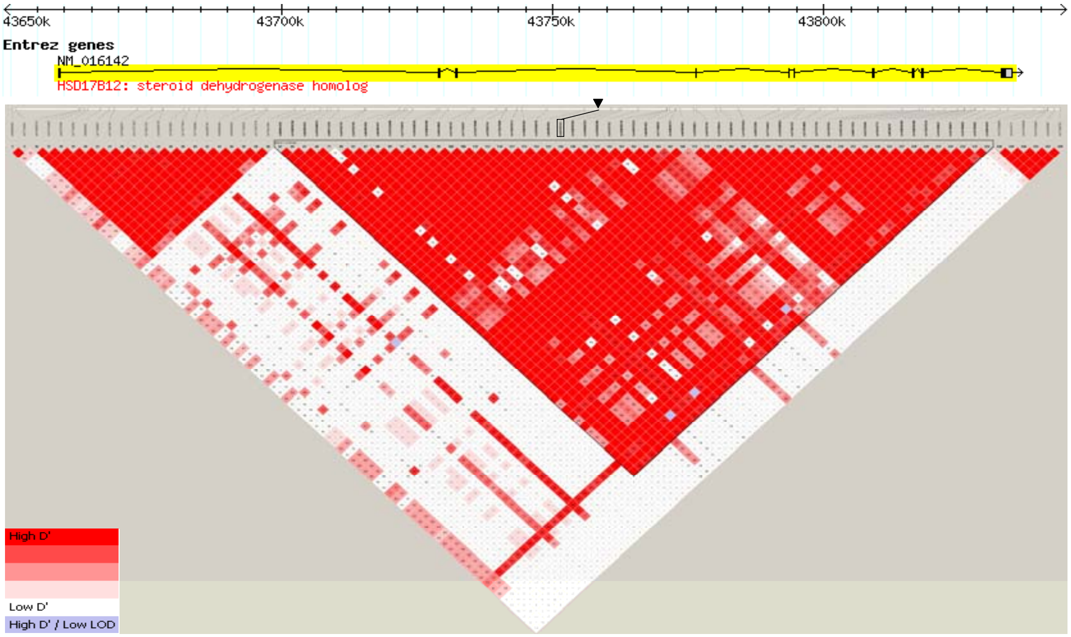

B.

Major haplotypes (>5%) delineated by SNPs passing selection criteria of frequency>20% and r^2>0.8:

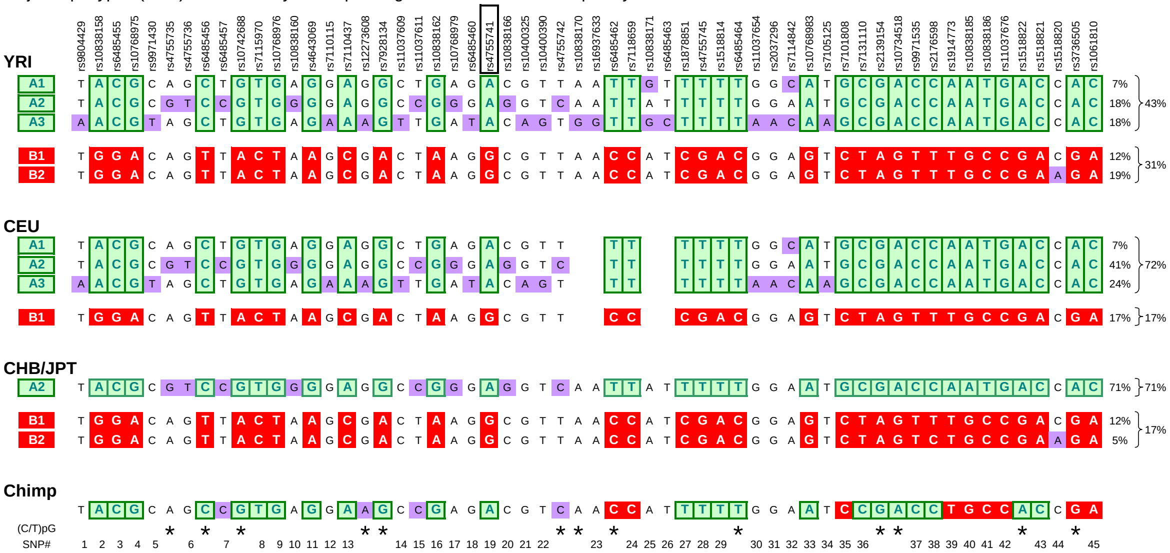

Figure S1: HSD17B12

C.

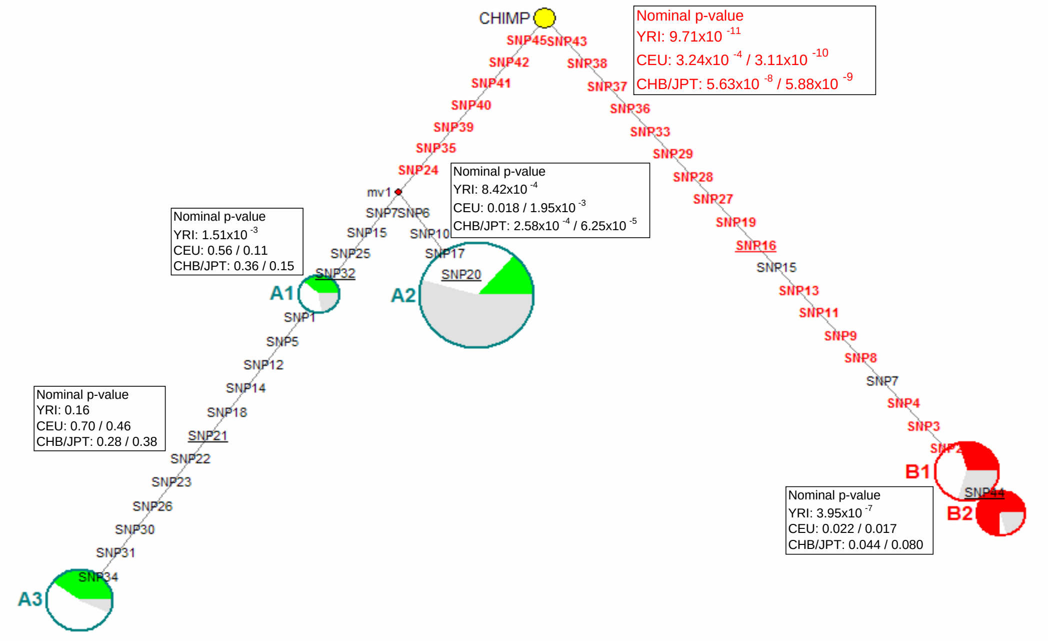

Figure S1: HSD17B12

IRF5

A.

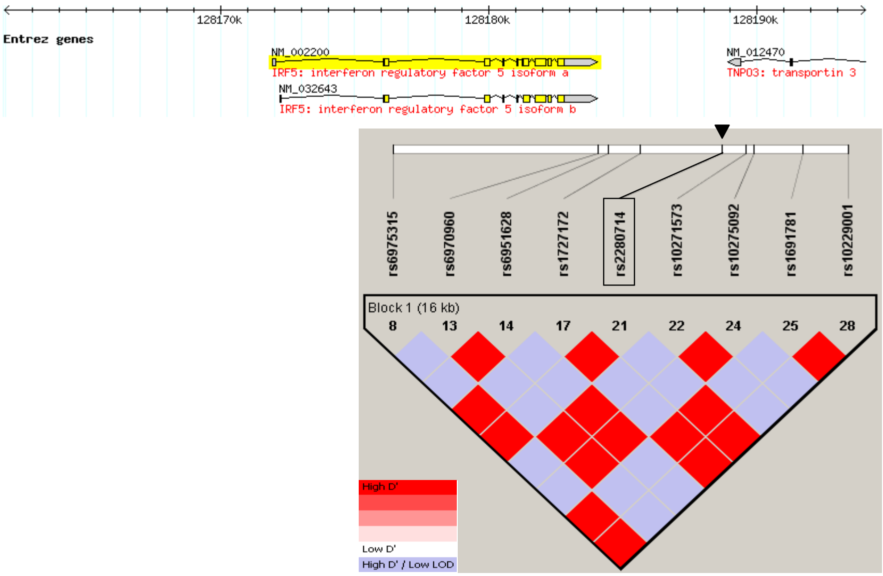

B.

Major haplotypes (>5%) delineated by SNPs passing selection criteria of rare allele frequency>5% and r^2>0.8:

Population	Haplotype	rs6975315	rs6970960	rs6951628	rs1727172	rs2280714	rs10271573	rs10275092	rs1691781	rs10229001	Frequency	Combined
YRI	A1	G	T	C	T	T	C	G	T	T	48%	55%
	A2	G	G	T	T	T	T	A	T	T	8%	
	B1	G	T	C	T	C	C	G	T	C	29%	43%
	B2	A	T	C	C	C	C	G	C	C	14%	
CEU	A1	G	T	C	T	T	C	G	T	T	54%	54%
	B1	G	T	C	T	C	C	G	T	C	40%	40%
CHB/JPT	A1	G	T	C	T	T	C	G	T	T	43%	43%
	B1	G	T	C	T	C	C	G	T	C	47%	47%
	A/B	G	T	C	T	T	C	G	T	C	10%	
Chimp		G	T	C	T	T	C	G	T	T		
(C/T)pG		*		*	*	*	*	*				
SNP#			1			2			3	4		

Figure S2: IRF5

C.

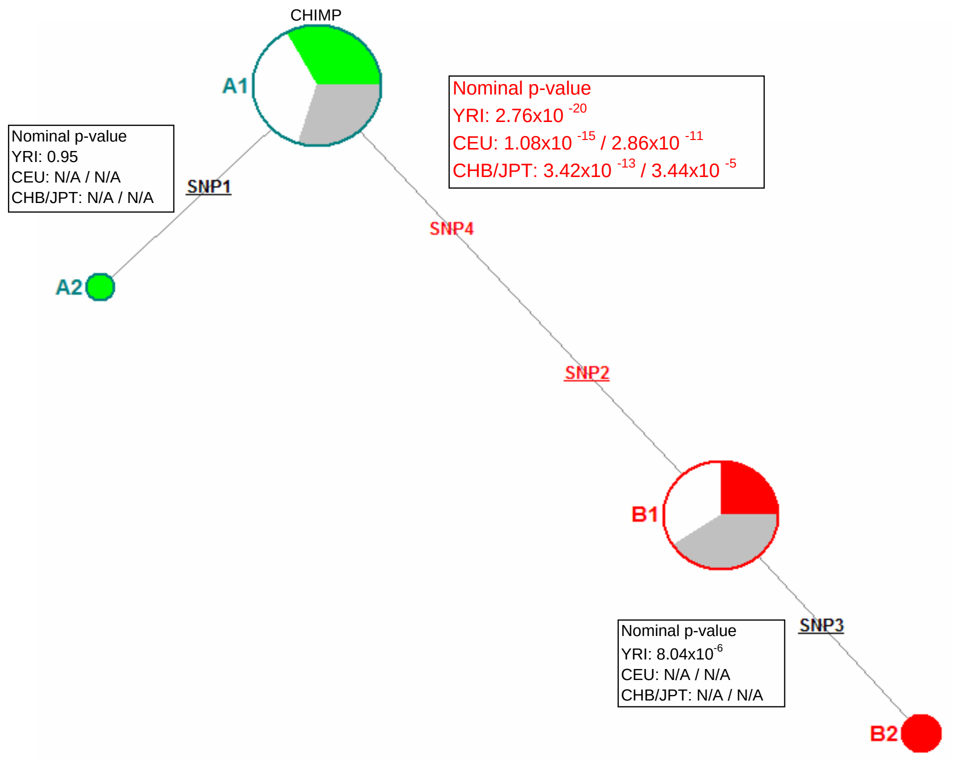

Figure S2: IRF5

CD151

A.

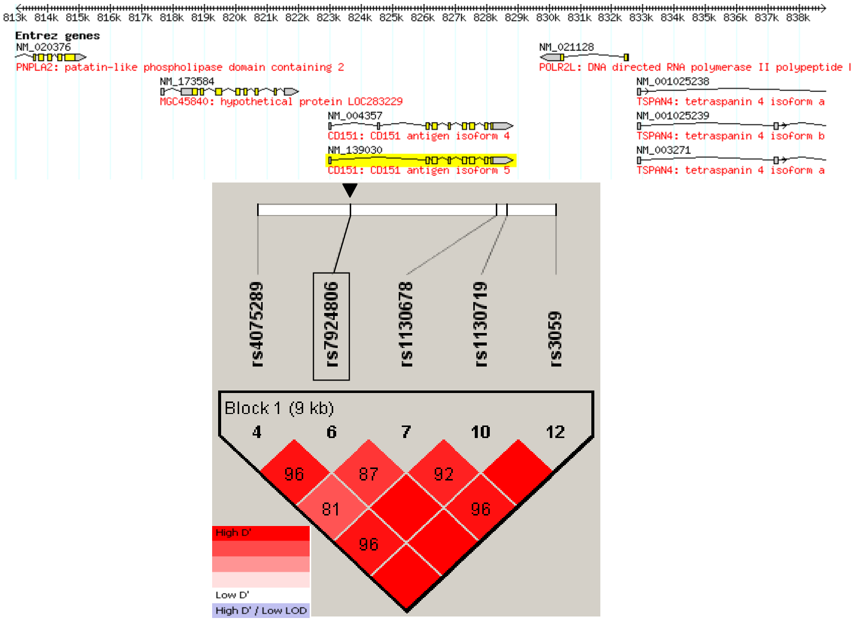

B.

Major haplotypes (>5%) delineated by SNPs passing selection criteria of rare allele frequency>5% and r^2>0.8:

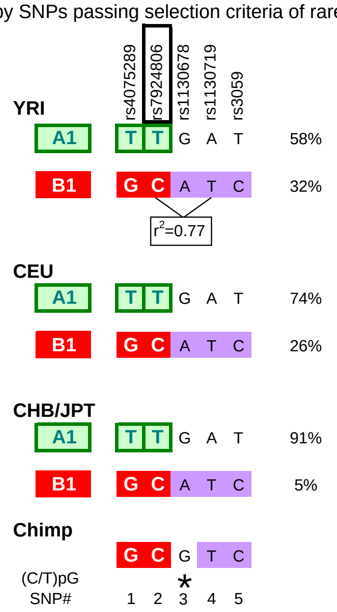

Figure S3: CD151

C.

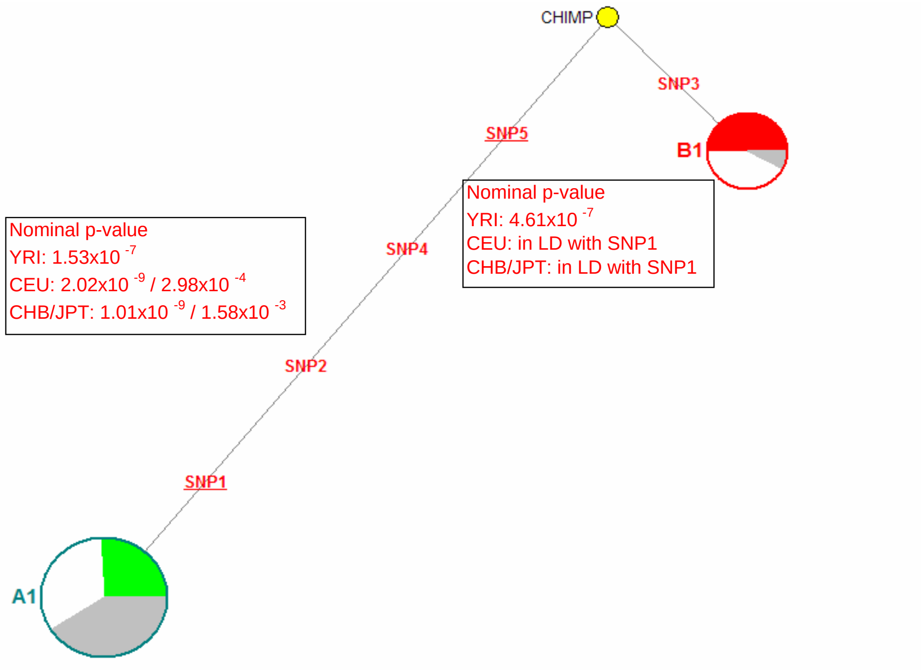

Figure S3: CD151

CCT8

A.

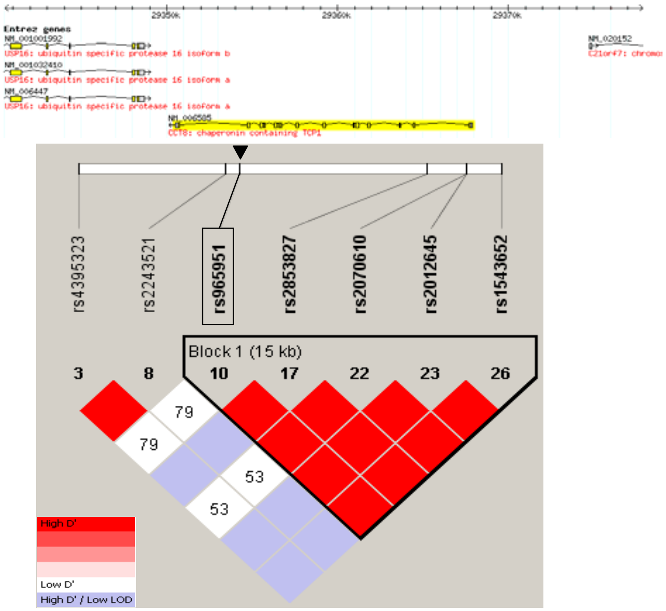

B.

Major haplotypes (>5%) delineated by SNPs passing selection criteria of rare allele frequency>5% and r^2>0.8:

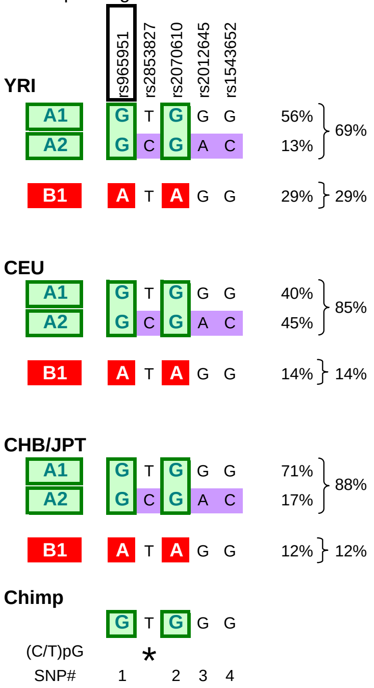

Figure S4: CCT8

C.

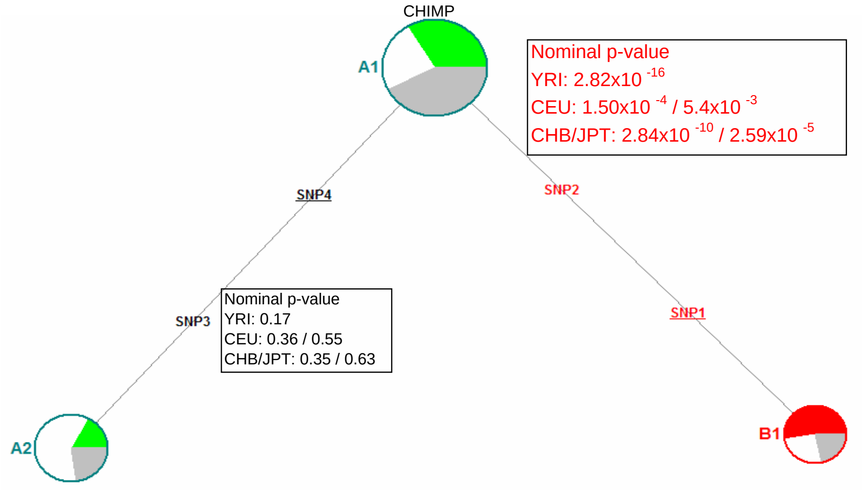

Figure S4: CCT8

PPAT

A.

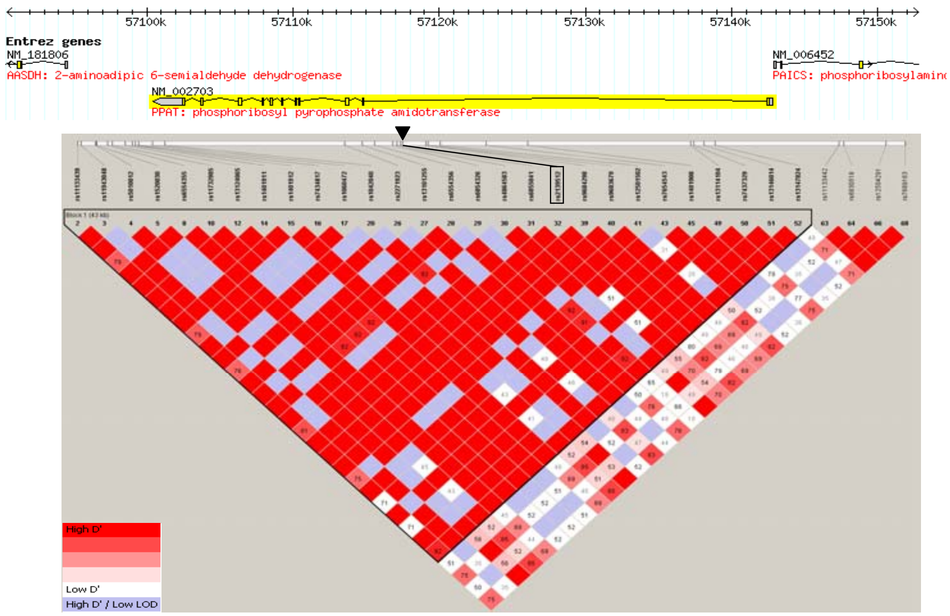

B.

Major haplotypes (>5%) delineated by SNPs passing selection criteria of rare allele frequency>5% and r^2>0.8:

Population	Haplotype	rs11943048	rs5010012	rs1520030	rs6554355	rs11732985	rs13124065	rs1401911	rs1401912	rs7434817	rs1060472	rs1042040	rs2271923	rs13101255	rs6554356	rs6854326	rs4864583	rs6855041	rs2139512	rs9684298	rs9683679	rs12501502	rs7654543	rs1401908	rs13114104	rs7437329	rs13146014	rs13147824	Freq.	Total
YRI	A1	G	A	G	G	T	A	T	C	C	C	C	T	C	T	T	C	A	C	A	T	T	A	C	C	T	C	T	14%	57%
	A2	G	T	C	G	T	A	T	C	T	A	C	C	C	T	T	T	T	C	A	T	C	A	C	C	T	C	T	22%	
	A3	G	A	G	G	A	C	C	C	C	C	G	T	G	T	T	C	A	C	A	T	T	C	T	C	A	G	T	5%	
	A4	A	A	G	G	A	C	C	C	C	C	G	T	G	T	C	C	A	C	A	T	T	C	T	C	A	G	T	14%	
	A5	G	A	C	G	T	A	T	C	T	C	C	C	C	T	T	C	T	C	A	T	C	A	T	C	A	C	T	2%	
	B1	G	A	G	A	T	A	T	T	C	C	C	T	C	C	T	C	A	G	G	A	T	A	C	A	T	C	C	33%	33%
CEU	A1	G	A	G	G	T	A	T	C	C	C	C	T	C	T	T	C	A	C	A	T	T	A	C	C	T	C	T	6%	67%
	A2	G	T	C	G	T	A	T	C	T	A	C	C	C	T	T	T	T	C	A	T	C	A	C	C	T	C	T	13%	
	A4	A	A	G	G	A	C	C	C	C	C	G	T	G	T	C	C	A	C	A	T	T	C	T	C	A	G	T	48%	
	B1	G	A	G	A	T	A	T	T	C	C	C	T	C	C	T	C	A	G	G	A	T	A	C	A	T	C	C	29%	29%
CHB/JPT	A1	G	A	G	G	T	A	T	C	C	C	C	T	C	T	T	C	A	C	A	T	T	A	C	C	T	C	T	23%	70%
	A2	G	T	C	G	T	A	T	C	T	A	C	C	C	T	T	T	T	C	A	T	C	A	C	C	T	C	T	25%	
	A4	A	A	G	G	A	C	C	C	C	C	G	T	G	T	C	C	A	C	A	T	T	C	T	C	A	G	T	6%	
	A5	G	A	C	G	T	A	T	C	T	C	C	C	C	T	T	C	T	C	A	T	C	A	T	C	A	C	T	17%	
	B1	G	A	G	A	T	A	T	T	C	C	C	T	C	C	T	C	A	G	G	A	T	A	C	A	T	C	C	28%	28%
Chimp		G	A	G	G	?	A	T	C	C	C	C	T	?	T	T	C	?	C	A	T	T	A	C	C	T	C	T		
(C/T)pG																				*		*		*				*		
SNP#		1	2	3	4		5	6	7	8	9	10	11		12	13	14		15		16		17		18	19	20			

Figure S5: PPAT

C.

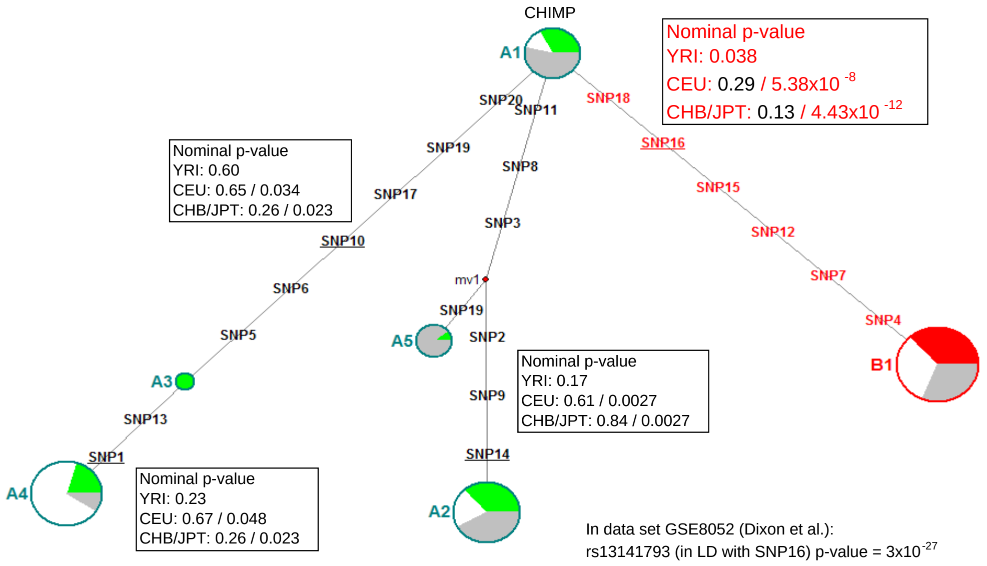

Figure S5: PPAT

LOC388796
(AA827892)

A.

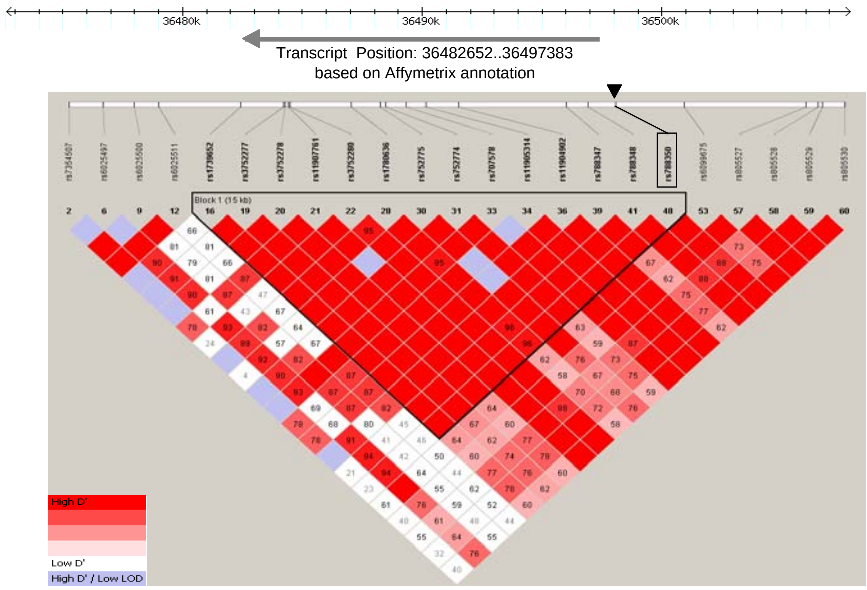

B.

Major haplotypes (>5%) delineated by SNPs passing selection criteria of rare allele frequency>5% and r^2>0.8:

	rs1739652	rs3752277	rs3752278	rs11907761	rs3752280	rs1780636	rs752775	rs752774	rs707578	rs11905314	rs11904902	rs788347	rs788348	rs788350		
**YRI**																
A1	A	G	C	C	A	T	T	G	C	A	C	C	G	G	38%	46%
A2	A	G	C	T	G	T	T	G	T	G	T	C	A	A	8%	
B1	G	A	T	C	G	C	T	A	T	A	C	T	A	A	10%	45%
B2	G	A	T	C	G	C	C	A	T	A	C	T	A	A	35%	
**CEU**																
A1	A	G	C	C	A	T	T	G	C	A	C	C	G	G	90%	90%
B2	G	A	T	C	G	C	C	A	T	A	C	T	A	A	8%	8%
**CHB/JPT**																
A1	A	G	C	C	A	T	T	G	C	A	C	C	G	G	82%	82%
B2	G	A	T	C	G	C	C	A	T	A	C	T	A	A	12%	12%
**Chimp**	A	G	C	C	G	C	T	A	T	A	C	C	A	A		
(C/T)pG	*	*	*		*	*		*								
SNP#			1	2			3		4	5	6	7	8	9		

Figure S6: LOC388796

C.

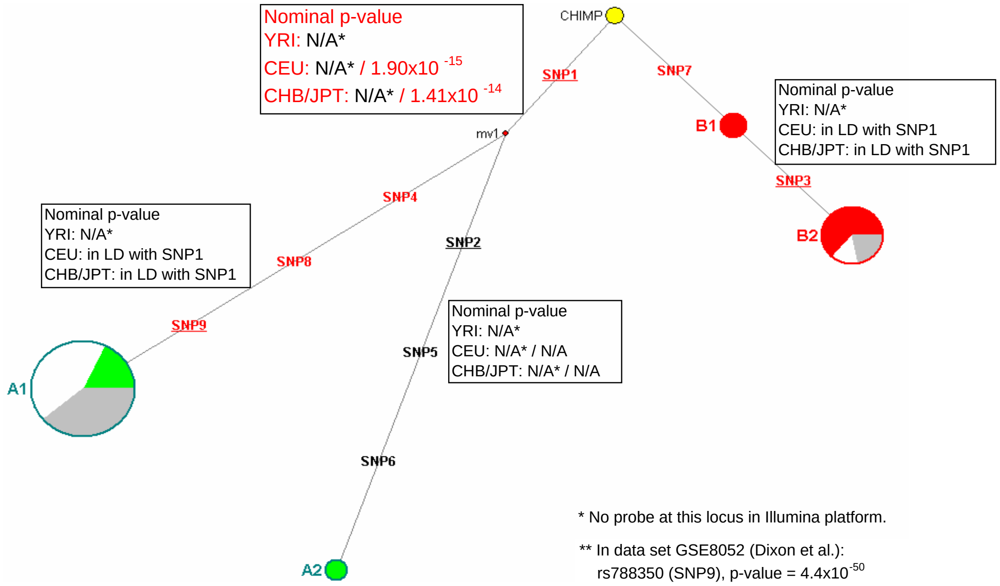

* No probe at this locus in Illumina platform.

** In data set GSE8052 (Dixon et al.): rs788350 (SNP9), p-value = 4.4×10^{-50}

Figure S6: LOC388796

TMEM8

A.

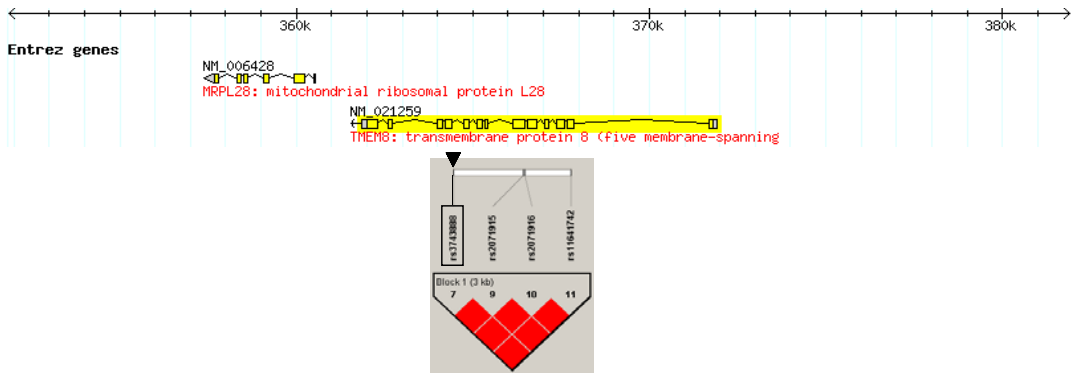

B.

Major haplotypes (>5%) delineated by SNPs passing selection criteria of rare allele frequency>5% and r^2>0.75:

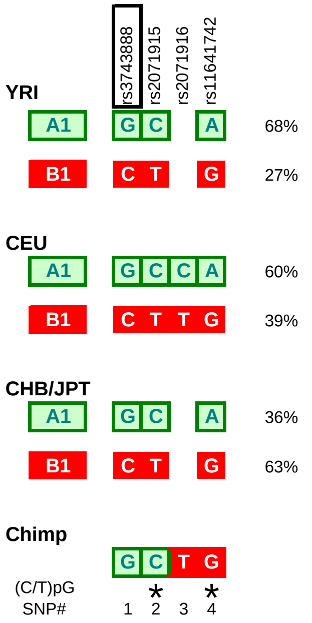

Figure S7: TMEM8

C.

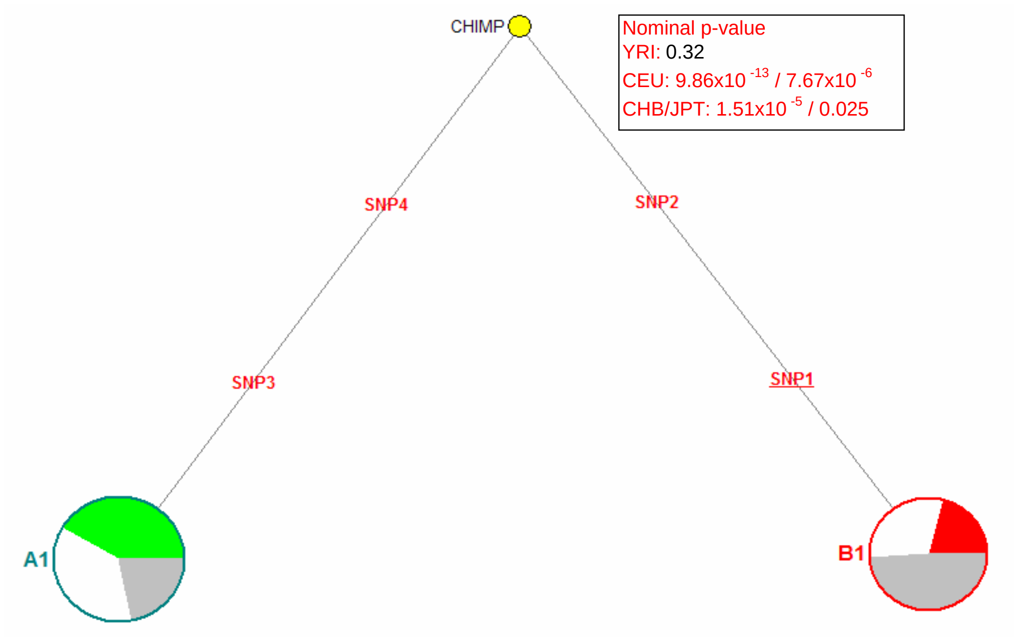

Figure S7: TMEM8

CTBP1

A.

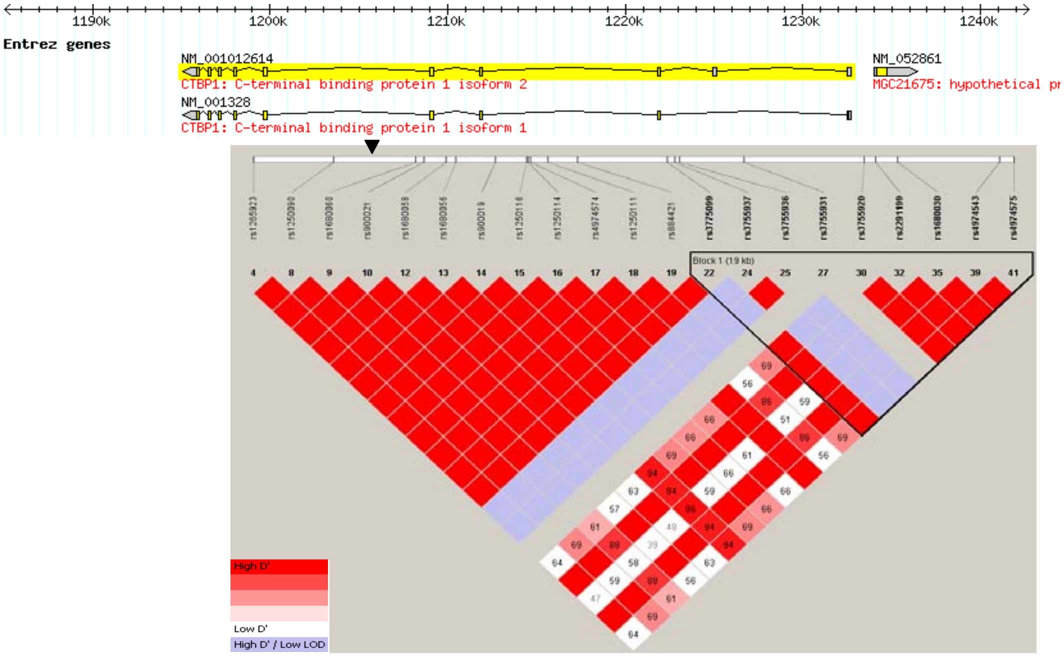

* LD Plot in CEU; No HapMap genotyping data for the originally reported peak SNP rs2279282 in current release.
** p-value insignificant for the tagSNP (rs900021) in the block containing the originally reported peak SNP.

B.

Major haplotypes (>5%) delineated by SNPs passing selection criteria of rare allele frequency>5% and r^2>0.8:

	rs3775099	rs3755937	rs3755936	rs3755931	rs3755920	rs2291199	rs2279280	rs1680030	rs4974543	rs4974575		
**YRI**												
A1	C	G	G	C	T	A	G	A	A	A	61%	61%
B1	C	G	G	C	C	A	G	A	A	G	17%	31%
B2	G	G	G	C	C	G	G	A	G	G	2%	
B3	C	C	C	T	C	A	A	A	A	G	13%	
**CEU**												
A1	C	G	G	C	T	A		A	A	A	15%	54%
A2	C	G	G	C	T	A		T	A	A	39%	
B1	C	G	G	C	C	A		A	A	G	7%	44%
B2	G	G	G	C	C	G		A	G	G	38%	
**CHB/JPT**												
A1	C	G	G	C	T	A		A	A	A	3%	30%
A2	C	G	G	C	T	A		T	A	A	27%	
B1	C	G	G	C	C	A		A	A	G	28%	66%
B2	G	G	G	C	C	G		A	G	G	22%	
B3	C	C	C	T	C	A		A	A	G	16%	
**Chimp**	C	G	G	C	C	A	G	A	A	G		
(C/T)pG				*		*	*			*		
SNP#	1	2	3		4			5	6			

Figure S8: CTBP1

C.

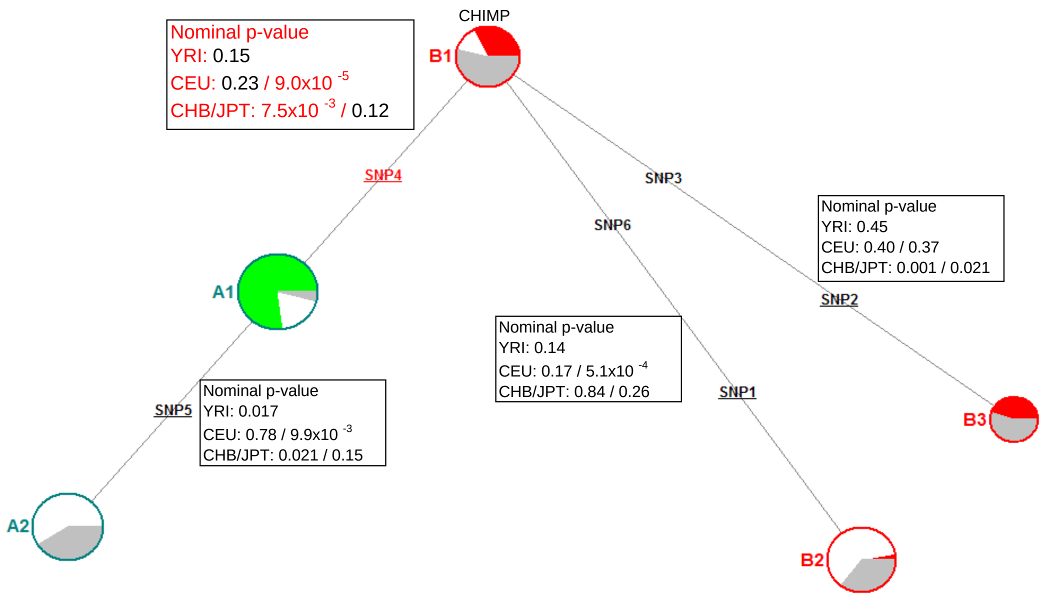

Figure S8: CTBP1

ATF5

A.

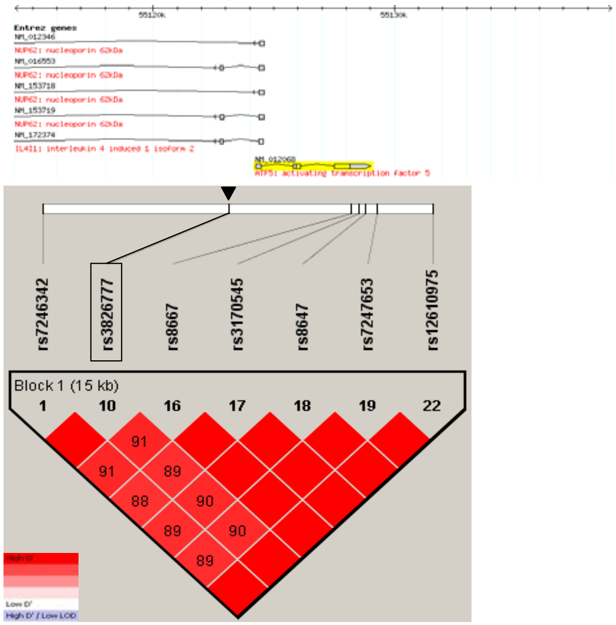

B.

Major haplotypes (>5%) delineated by SNPs passing selection criteria of rare allele frequency>5% and r^2>0.8:

YRI

	rs7246342	rs3826777	rs8667	rs3170545	rs8647	rs12610975		
A1	G	G	G	G	G	G	82%	82%
B1	A	A	G	G	G	G	6%	16%
B2	A	A	A	A	A	A	10%	

CEU

	rs7246342	rs3826777	rs8667	rs3170545	rs8647	rs12610975		
A1	G	G	G	G	G	G	59%	59%
B1	A	A	G	G	G	G	3%	34%
B2	A	A	A	A	A	A	31%	

CHB/JPT

	rs7246342	rs3826777	rs8667	rs3170545	rs8647	rs12610975		
A1	G	G	G	G	G	G	48%	48%
B1	A	A	G	G	G	G	4%	45%
B2	A	A	A	A	A	A	41%	

Chimp

	rs7246342	rs3826777	rs8667	rs3170545	rs8647	rs12610975
	G	G	G	G	G	G
(C/T)pG					*	
SNP#	1	2	3	4		5

Figure S9: ATF5

C.

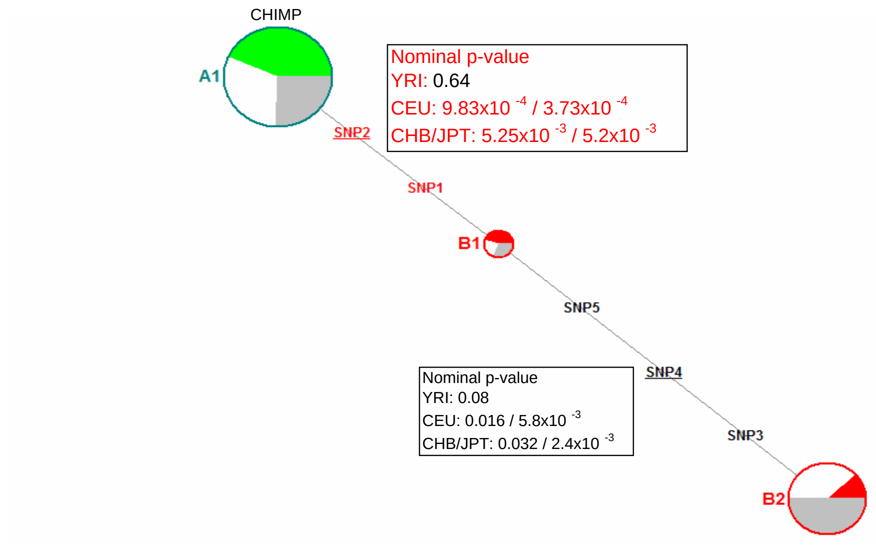

Figure S9: ATF5

ARTS1

A.

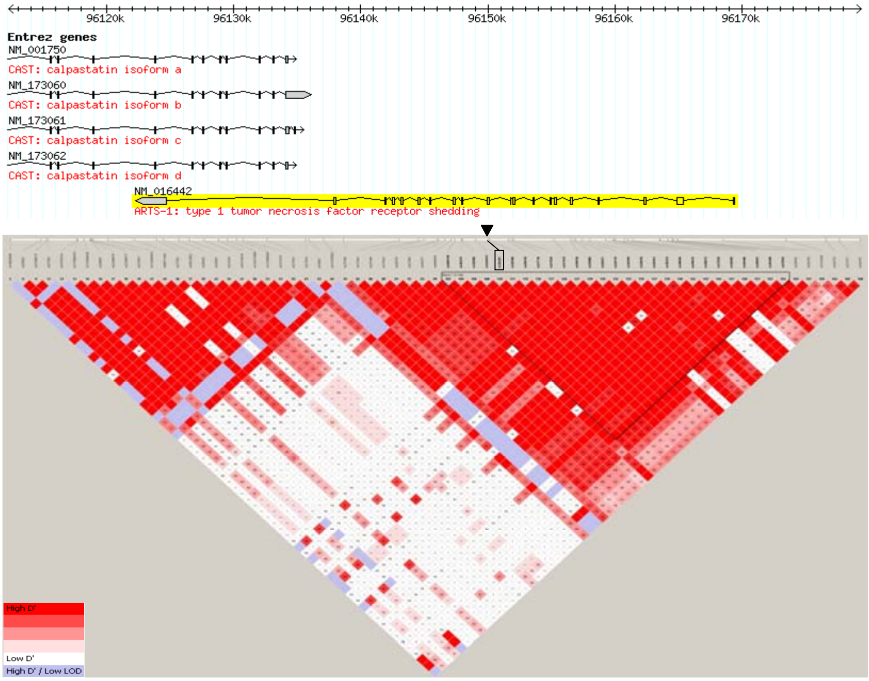

B.

Major haplotypes (>5%) delineated by SNPs passing selection criteria of rare allele frequency>10% and r^2>0.8:

		rs469758	rs30379	rs30380	rs246453	rs151928	rs30187	rs30186	rs26510	rs27710	rs27529	rs27434	rs26618	rs25866	rs26617	rs28119	rs26616	rs26491	rs30333	rs26615	rs26613	rs27036	rs26495	rs26496	rs26652	rs26498	rs27045		
**YRI**	A1	T	G	C	C	G	C	C	T	G	G	G	T	C	C	A	C	G	G	T	C	C	G	G	G	G	T	8%	51%
	A2	T	G	C	C	G	C	C	T	G	G	G	T	A	C	A	C	G	A	T	C	T	G	G	G	G	T	21%	
	A3	T	G	C	C	T	C	C	T	G	G	G	C	C	T	A	G	G	G	G	T	C	G	G	A	G	T	21%	
	B1	C	T	A	T	G	T	G	C	A	A	A	T	C	C	A	C	G	G	T	C	C	A	A	G	A	C	7%	40%
	B2	C	T	A	T	G	T	G	C	A	A	A	T	C	C	G	C	A	G	T	C	C	A	A	G	A	C	33%	
**CEU**	A1	T	G	C	C	G	C	C	T	G	G	G	T	C	C	A	C	G		T	C	C	G	G	G	G	T	32%	68%
	A2	T	G	C	C	G	C	C	T	G	G	G	T	A	C	A	C	G		T	C	T	G	G	G	G	T	10%	
	A3	T	G	C	C	T	C	C	T	G	G	G	C	C	T	A	G	G		G	T	C	G	G	A	G	T	25%	
	B2	C	T	A	T	G	T	G	C	A	A	A	T	C	C	G	C	A		T	C	C	A	A	G	A	C	19%	28%
	B/A2	C	T	A	T	G	T	C	C	A	A	G	T	A	C	A	C	G		T	C	T	G	G	G	G	T	10%	
**CHB/JPT**	A1	T	G	C	C	G	C	C	T	G	G	G	T	C	C	A	C	G	G	T	C	C	G	G	G	G	T	5%	49%
	A2	T	G	C	C	G	C	C	T	G	G	G	T	A	C	A	C	G	A	T	C	T	G	G	G	G	T	24%	
	A3	T	G	C	C	T	C	C	T	G	G	G	C	C	T	A	G	G	G	G	T	C	G	G	A	G	T	20%	
	B2	C	T	A	T	G	T	G	C	A	A	A	T	C	C	G	C	A	G	T	C	C	A	A	G	A	C	45%	45%
**Chimp**		T	G	A	C	G	T	C	C	A	A	G	T	C	C	A	C	G	G	T	C	C	A	G	G	G	T		
(C/T)pG											*				*				*										
SNP#		1	2	3	4	5	6	7	8	9		10	11	12		13	14	15		16	17	18		19	20	21	22		

Figure S10: ARTS-1

C.

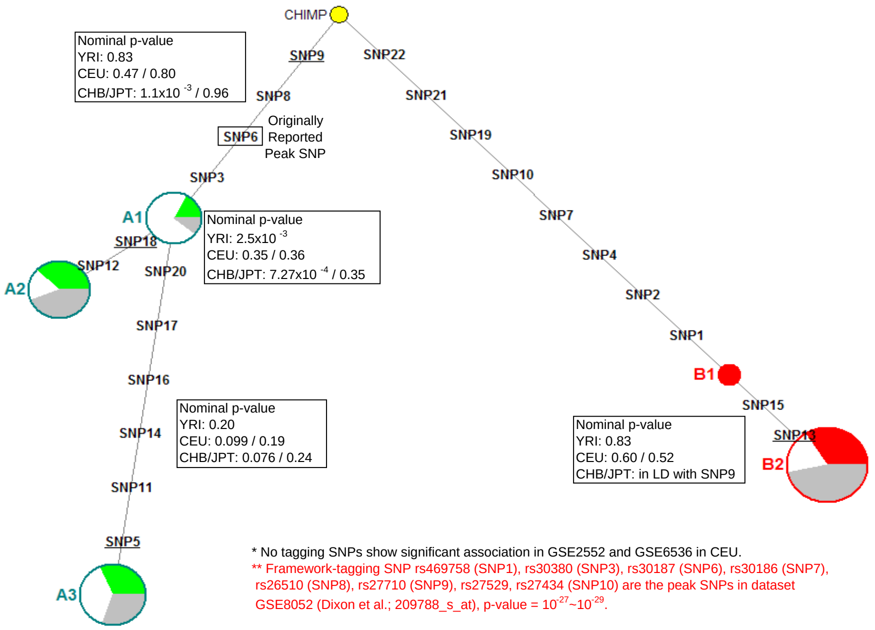

Figure S10: ARTS-1

IL16

A.

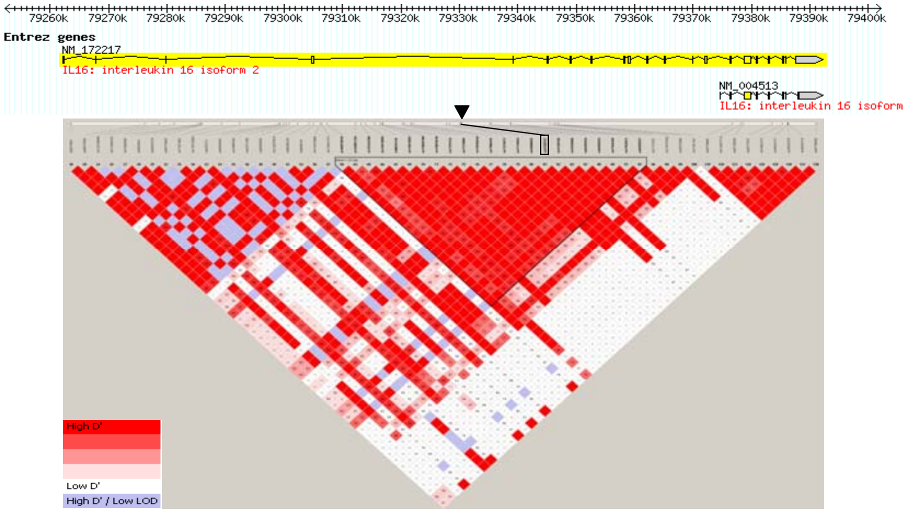

B.

Major haplotypes (>5%) delineated by SNPs passing selection criteria of rare allele frequency>10% and r^2>0.8:

Population	Haplotype	rs16972819	rs12324786	rs11072995	rs8034716	rs11072997	rs11856780	rs11853619	rs7179134	rs7172689	rs11638444	rs7180245	rs7172611	rs7174055	rs4496093	rs4128767	rs4128766	rs4344696	rs6495554	rs6495555	rs7164240	rs7163931	rs6495557	Freq.	Group
YRI	A1	G	C	A	A	C	G	T	G	C	T	G	G	G	A	G	A	A	T	T	G	C	A	12%	52%
	A2	G	C	A	A	C	G	T	G	C	T	G	A	G	A	G	G	A	T	T	G	C	A	17%	
	A3	G	C	A	A	C	G	T	G	C	T	G	A	G	A	A	G	C	T	T	G	C	A	23%	
	B1	G	T	T	A	T	A	C	A	T	C	A	G	G	A	G	A	A	C	C	G	G	A	18%	37%
	B2	A	T	T	G	T	A	C	A	T	C	A	G	A	G	G	A	A	C	C	T	G	G	18%	
CEU	A2	G	C	A	A	C	G	T	G	C	T	G	A	G	A	G	G	A	T	T	G	C	A	14%	76%
	A3	G	C	A	A	C	G	T	G	C	T	G	A	G	A	A	G	C	T	T	G	C	A	62%	
	B1	G	T	T	A	T	A	C	A	T	C	A	G	G	A	G	A	A	C	C	G	G	A	22%	22%
CHB/JPT	A2	G	C	A	A	C	G	T	G	C	T	G	A	G	A	G	G	A	T	T	G	C	A	24%	97%
	A3	G	C	A	A	C	G	T	G	C	T	G	A	G	A	A	G	C	T	T	G	C	A	73%	
	B1	G	T	T	A	T	A	C	A	T	C	A	G	G	A	G	A	A	C	C	G	G	A	2%	2%
Chimp		G	T	A	A	T	A	C	G	T	T	A	G	G	A	C	T	A	C	T	G	G	A		
		*	1	2	3	4	*	5	*	6	7	*	8	9	*	10	11	12	13	14	15	16	*		

Figure S11: IL16

C.

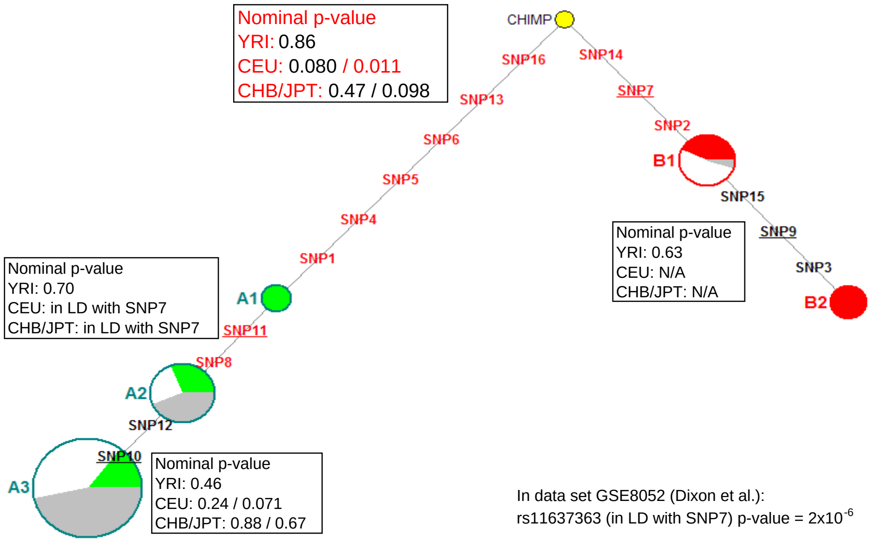

In data set GSE8052 (Dixon et al.):
rs11637363 (in LD with SNP7) p-value = $2x10^{-6}$

Figure S11: IL16

CTSH

A.

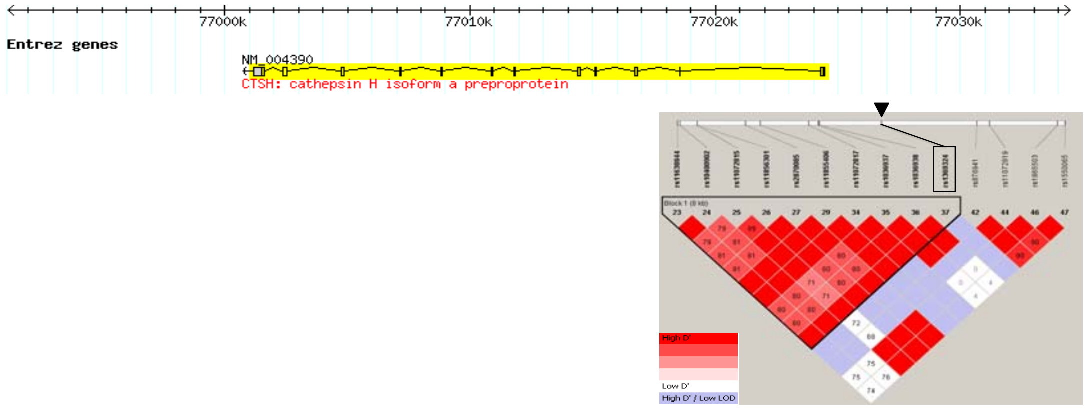

B.

Major haplotypes (>5%) delineated by SNPs passing selection criteria of rare allele frequency>10% and r^2>0.8:

YRI

	rs11638844	rs10400902	rs11072815	rs11856301	rs2870085	rs11855406	rs11072817	rs1036937	rs1036938	rs1369324		
A1	A	A	A	T	T	A	G	C	C	A	81%	82%
A2	G	G	A	T	T	A	G	C	C	A	2%	
B1	A	A	A	T	T	A	G	A	G	A	6%	10%
B2	G	G	G	C	C	G	A	A	G	G	4%	

CEU

	rs11638844	rs10400902	rs11072815	rs11856301	rs2870085	rs11855406	rs11072817	rs1036937	rs1036938	rs1369324		
A1	A	A	A	T	T	A	G	C	C	A	28%	28%
B2	G	G	G	C	C	G	A	A	G	G	68%	68%

CHB/JPT

	rs11638844	rs10400902	rs11072815	rs11856301	rs2870085	rs11855406	rs11072817	rs1036937	rs1036938	rs1369324		
A1	A	A	A	T	T	A	G	C	C	A	55%	87%
A2	G	G	A	T	T	A	G	C	C	A	32%	
B2	G	G	G	C	C	G	A	A	G	G	13%	13%

Chimp

	rs11638844	rs10400902	rs11072815	rs11856301	rs2870085	rs11855406	rs11072817	rs1036937	rs1036938	rs1369324
Chimp	A	G	G	C	T	A	G	C	G	?
(C/T)pG			*			*	*			*
SNP#	1		2	3	4	5	6	7	8	

Figure S12: CTSH

C.

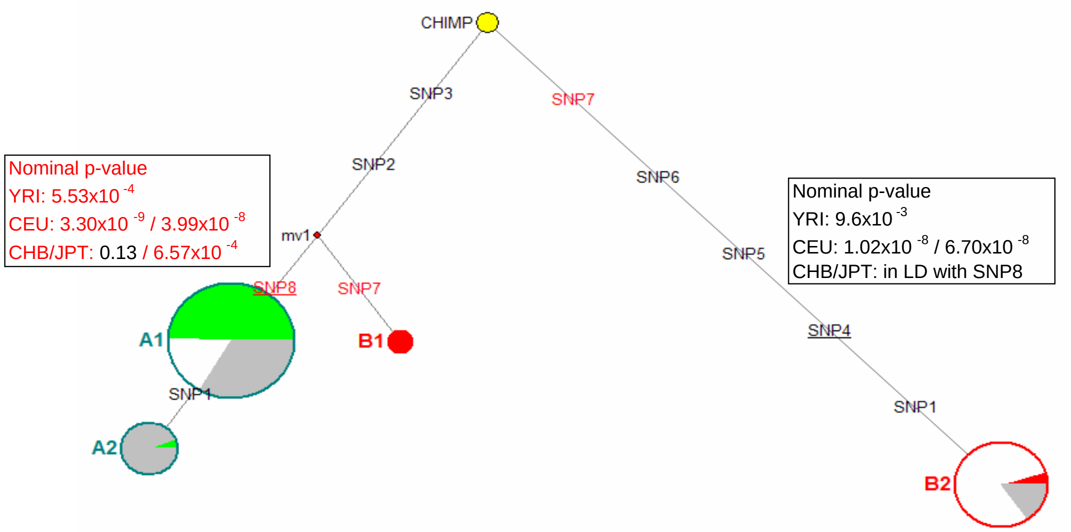

Figure S12: CTSH

CHI3L2

A.

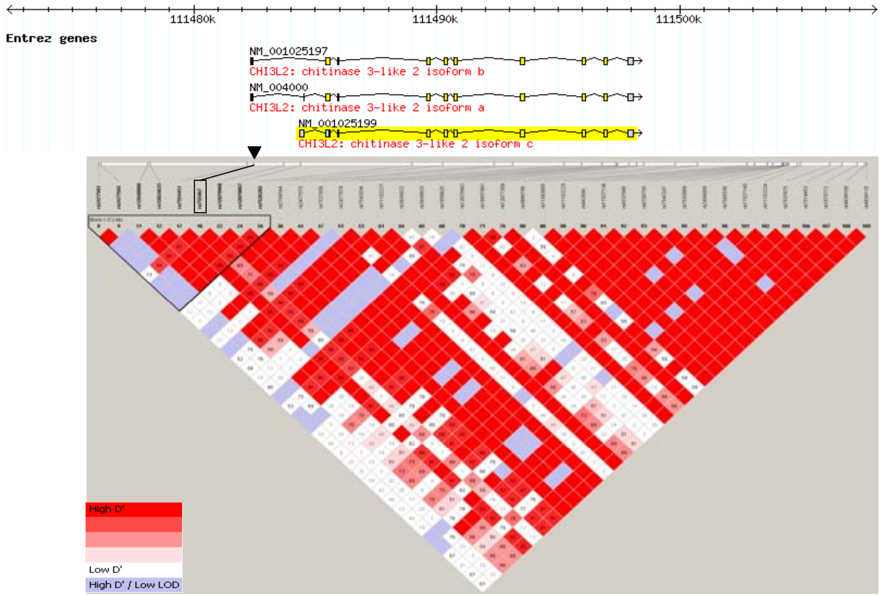

B.

Major haplotypes (>5%) delineated by SNPs passing selection criteria of rare allele frequency>10% and r^2>0.8:

Population	Haplotype	rs2477583	rs2477584	rs12048900	rs12024633	rs7554451	rs755467	rs12079968	rs12070867	rs7526282	Frequency	Total
YRI	A1	C	C	A	G	C	G	C	G	A	11%	70%
	A1-T	C	C	A	G	C	T	C	G	A	28%	
	A2-T	T	G	A	G	C	T	C	G	A	9%	
	A3	C	C	A	G	T	G	T	T	T	22%	
	B1	C	C	T	C	C	G	C	G	A	26%	26%
CEU	A1	C	C	A	G	C	G	C	G	A	21%	62%
	A1-T	C	C	A	G	C	T	C	G	A	25%	
	A2	T	G	A	G	C	G	C	G	A	17%	
	B1	C	C	T	C	C	G	C	G	A	33%	33%
CHB/JPT	A1	C	C	A	G	C	G	C	G	A	38%	87%
	A1-T	C	C	A	G	C	T	C	G	A	35%	
	A3	C	C	A	G	T	G	T	T	T	3%	
	A2	T	G	A	G	C	G	C	G	A	12%	
	B1	C	C	T	C	C	G	C	G	A	13%	13%

* rs755467 did not pass LD criterion in all three populations.

Chimp	C	G	A	G	C	G	C	G	G
	1	2	3	4	5	6	7	8	

Figure S13: CHI3L2

C.

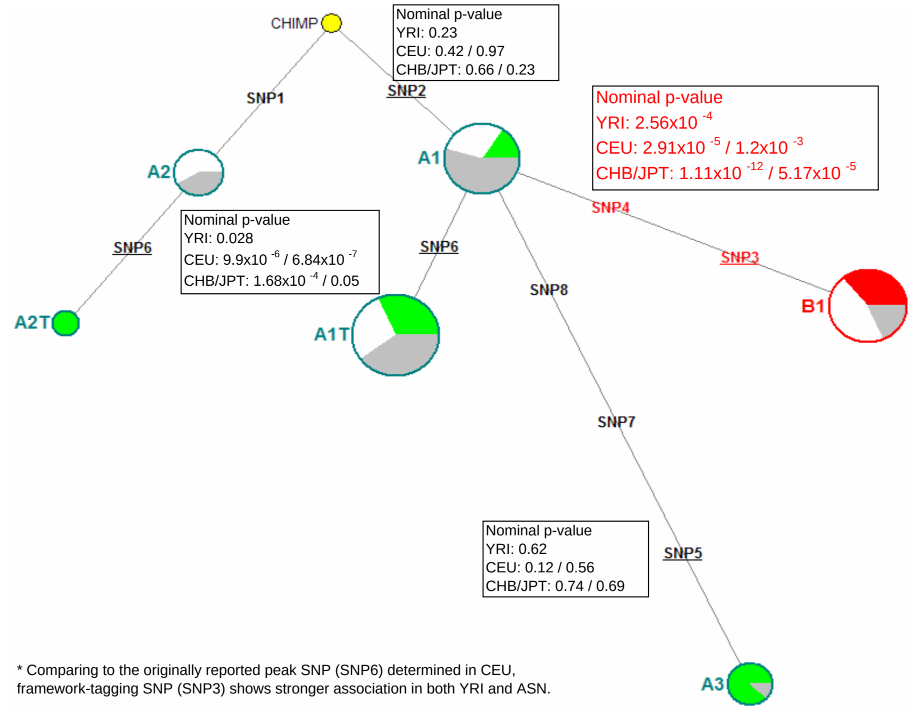

* Comparing to the originally reported peak SNP (SNP6) determined in CEU, framework-tagging SNP (SNP3) shows stronger association in both YRI and ASN.

Figure S13: CHI3L2

VAMP8

A.

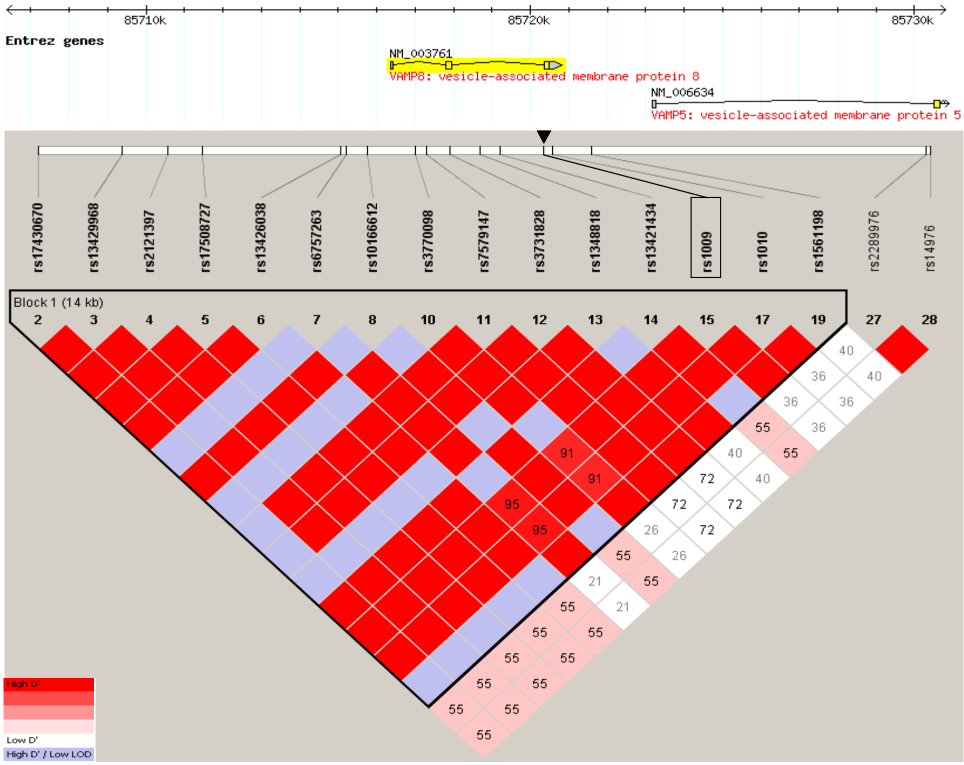

B.

Major haplotypes (>5%) delineated by SNPs passing selection criteria of rare allele frequency>5% and r^2>0.8:

	rs17430670	rs13429968	rs2121397	rs17508727	rs13426038	rs6757263	rs10166612	rs3770098	rs7579147	rs3731828	rs1348818	rs13421434	rs1009	rs1010	rs1561198		
**YRI**																	
A1	G	A	G	G	G	C	A	A	A	T	A	C	G	C	T	42%	42%
B1	G	A	G	G	G	C	A	A	G	C	A	C	G	C	T	9%	56%
B2	G	A	G	G	G	C	A	A	G	C	A	C	A	T	T	7%	
B3	G	A	G	G	G	T	A	C	G	C	A	C	A	T	T	8%	
B4	G	A	G	G	G	T	A	C	G	C	T	C	A	T	C	23%	
B5	T	G	A	A	C	C	G	A	G	C	A	A	G	C	T	10%	
**CEU**																	
A1	G	A	G	G	G	C	A	A	A	T	A	C	G	C	T	38%	38%
B3	G	A	G	G	G	T	A	C	G	C	A	C	A	T	T	3%	63%
B4	G	A	G	G	G	T	A	C	G	C	T	C	A	T	C	48%	
B5	T	G	A	A	C	C	G	A	G	C	A	A	G	C	T	12%	
**CHB/JPT**																	
A1	G	A	G	G	G	C	A	A	A	T	A	C	G	C	T	31%	31%
B4	G	A	G	G	G	T	A	C	G	C	T	C	A	T	C	64%	67%
B5	T	G	A	A	C	C	G	A	G	C	A	A	G	C	T	3%	
**Chimp**	T	A	G	G	A	T	G	A	G	C	A	C	G	C	T		
(C/T)pG		*				*								*			
SNP#	1		2	3			4	5	6	7	8	9	10		11		

Figure S14: VAMP8

C.

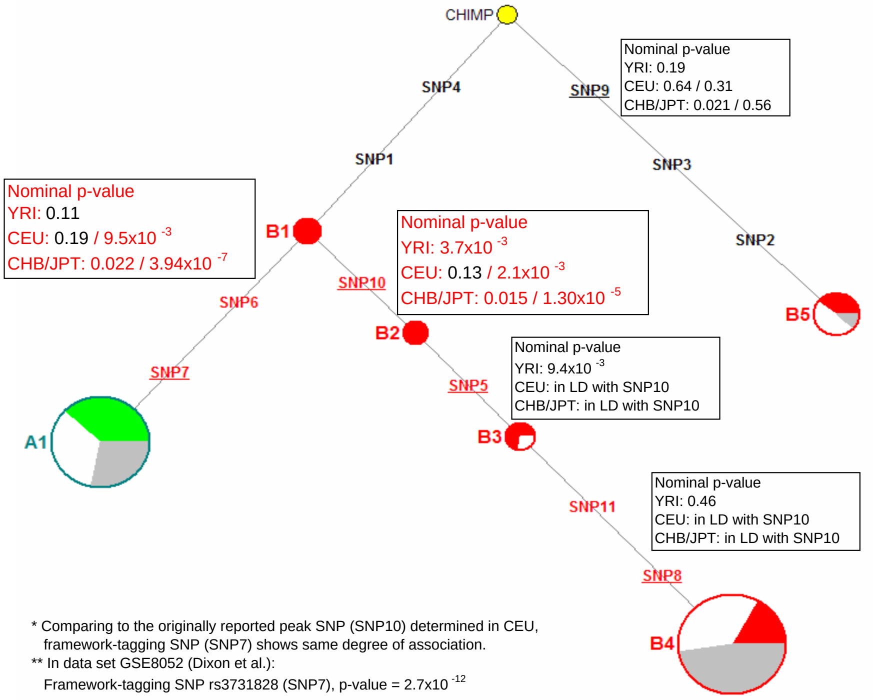

* Comparing to the originally reported peak SNP (SNP10) determined in CEU, framework-tagging SNP (SNP7) shows same degree of association.

** In data set GSE8052 (Dixon et al.): Framework-tagging SNP rs3731828 (SNP7), p-value = 2.7×10^{-12}

Figure S14: VAMP8

BTN3A2

A.

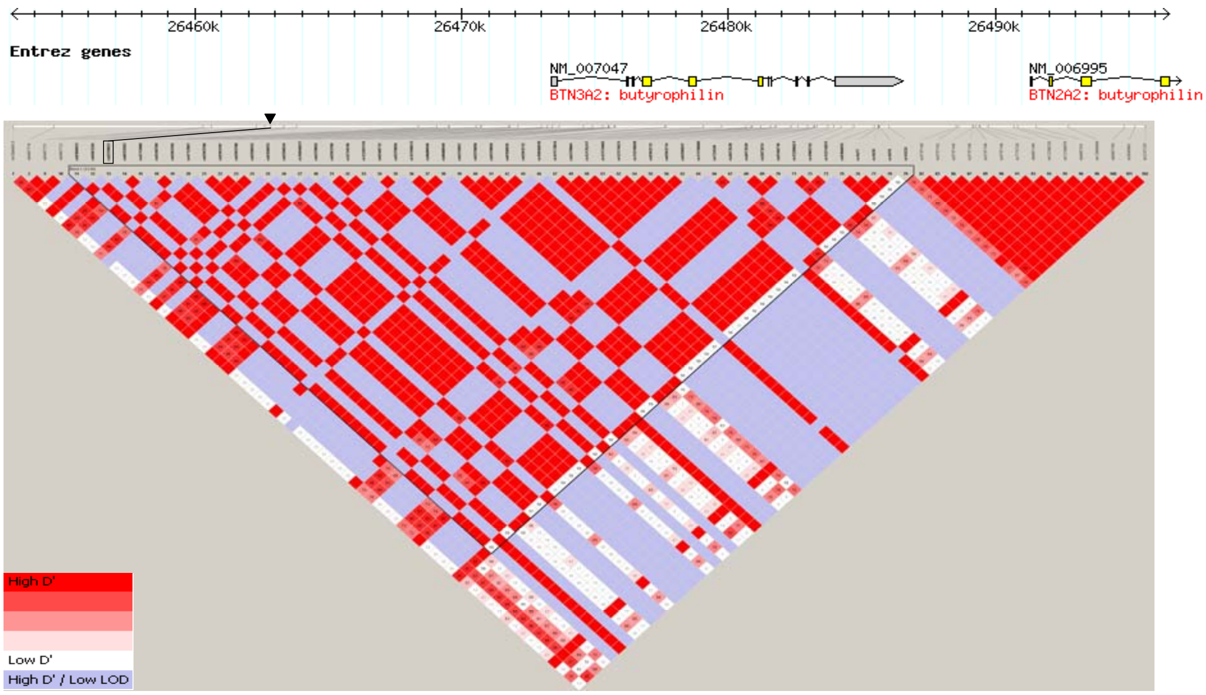

B.

Major haplotypes (>5%) delineated by SNPs passing selection criteria of rare allele frequency>10% and r^2>0.8:

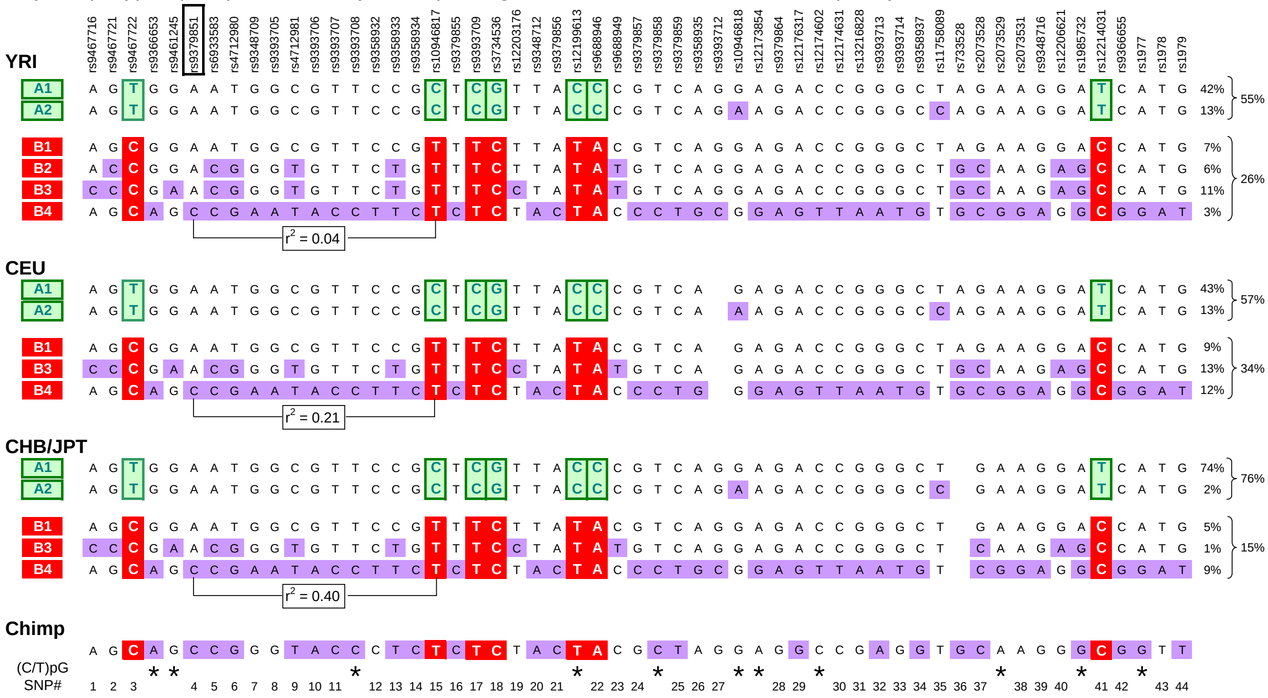

Figure S15: BTN3A2

C.

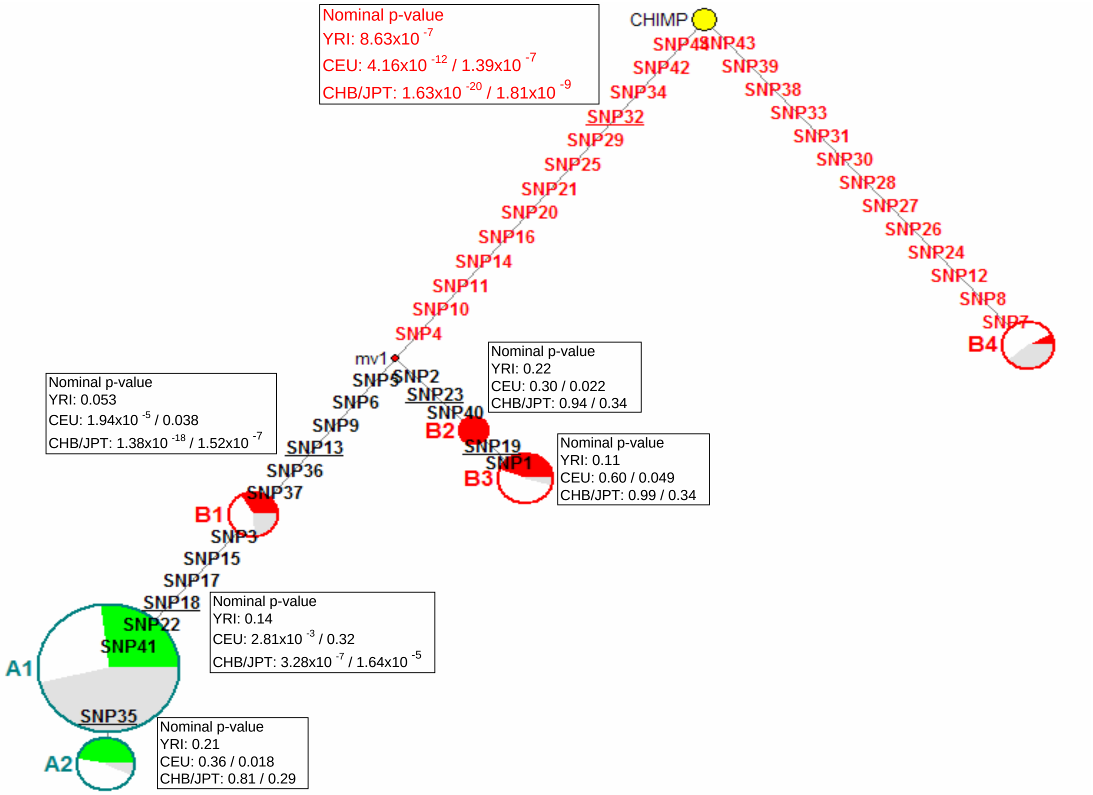

Figure S15: BTN3A2

SERPINB10

A.

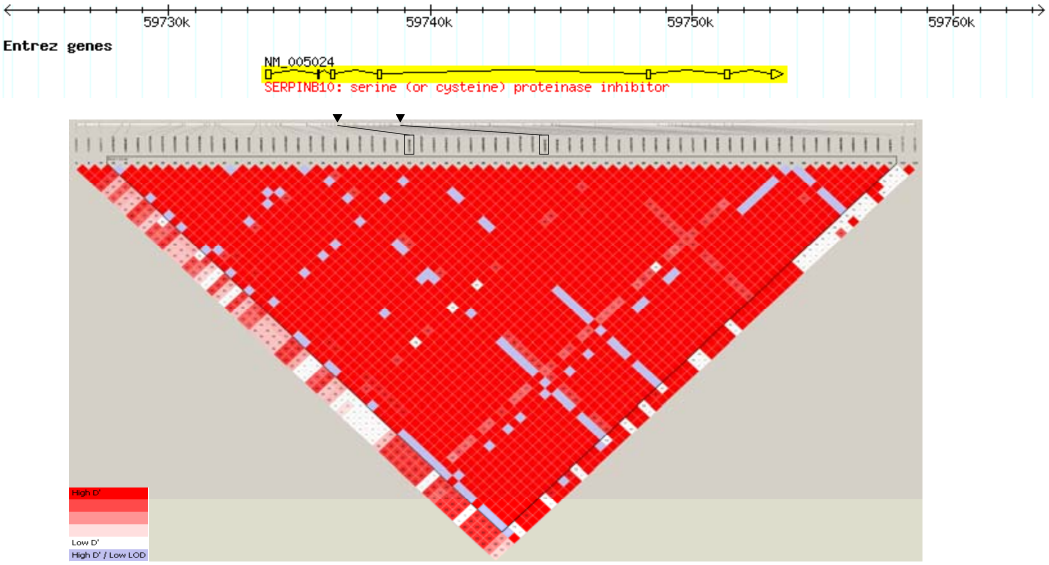

B.

Major haplotypes (>5%) delineated by SNPs passing selection criteria of rare allele frequency>5% and r^2>0.8:

SNPs (in order): rs17072077, rs9966367, rs4499334, rs12717154, rs13371114, rs12327005, rs12327162, rs12327006, rs12327173, rs8086765, rs8085904, rs7241748, rs9952843, rs9953733, rs17072097, rs8097425, rs17072100, rs17072102, rs8085341, rs8084931, rs17072109, rs17072114, rs9320033, rs724557, rs724558, rs17072127, rs1944273, rs1944272, rs1944271, rs9955171, rs8085490, rs8099238, rs9955876, rs17072146, rs17072152, rs620535, rs976038, rs9947135, rs9947504, rs7236189, rs17072163, rs976142, rs2042729, rs2162348, rs8097354, rs8093162, rs9957733, rs2114377, rs9960901, rs8090188, rs8093894, rs8094058, rs8089023, rs8095974, rs3786340, rs10163726, rs17072188, rs9951724, rs1544899, rs1544898, rs8084306, rs8096543, rs4630651, rs9964049, rs963075

Population	Haplotype	Alleles	Frequency	Group total
YRI	A1	T G G A G T C A T T G C G C T G T T G T A C T A T C G G C T C C A A A G G C C T G T C C C G A A A G C C A A T G T T C T C A C T T	10%	41%
	A2	T G G A G T C A T T G C G C T G T T G T A C T A T C G G C T C C A A A G G C C T G T T C C G A A A G C C A G T G T T C T C A C T T	31%	
	B1	T G A G A A C G G T A C A A G G T C A T G T T T C C C C T C C C T G G A G T T C A C C G T C G A A G C C A A T G T T C T T A C T T	17%	40%
	B2	G G A G A A C G G T A C A A G G G C A T G T T T C A C C T C C C T G G G G T T C A C C G T C G A A G C C A A T G A T C T T A C T T	11%	
	B3	T A G A G T T A T C G T G C T A T T A C A T C A T C G G C T T A A G G G C C C C A C C G T C A G T A T T G A C A T A T A T G T A C	13%	
CEU	B1	T G A G A A C G G T A C A A G G T C A T G T T T C C C C T C C C T G G A G T T C A C C G T C G A A G C C A A T G T T C T T A C T T	19%	97%
	B3	T A G A G T T A T C G T G C T A T T A C A T C A T C G G C T T A A G G G C C C C A C C G T C A G T A T T G A C A T A T A T G T A C	68%	
	B3/B1	T A G A G T T A T C G T G C T A T T A C A T C A T C G G C T T A A G G G C C C C A C C G T C G A A G C C A A T G T T C T T A C T T	9%	
CHB/JPT	A2	T G G A G T C A T T G C G C T G T T G T A C T A T C G G C T C C A A A G G C C T G T T C C G A A A G C C A G T G T T C T C A C T T	22%	22%
	B1	T G A G A A C G G T A C A A G G T C A T G T T T C C C C T C C C T G G A G T T C A C C G T C G A A G C C A A T G T T C T T A C T T	18%	77%
	B3	T A G A G T T A T C G T G C T A T T A C A T C A T C G G C T T A A G G G C C C C A C C G T C A G T A T T G A C A T A T A T G T A C	59%	
Chimp		T A A A A T C A T T G C A A G G T C A T G T T A T C C C C C C C A G G A G C C C G C C C C C A A A G T C G A T A T A C A T G T A C		

(C/T)pG * * * * * * * * * * * * *

SNP# 1 2 3 4 5 6 7 8 9 10 11 12 13 14 15 16 17 18 19 20 21 22 23 24 25 26 27 28 29 30 31 32 33 34 35 36 37 38 39 40 41 42 43 44 45 46 47 48 49 50 51 52

Figure S16: SERPINB10

C.

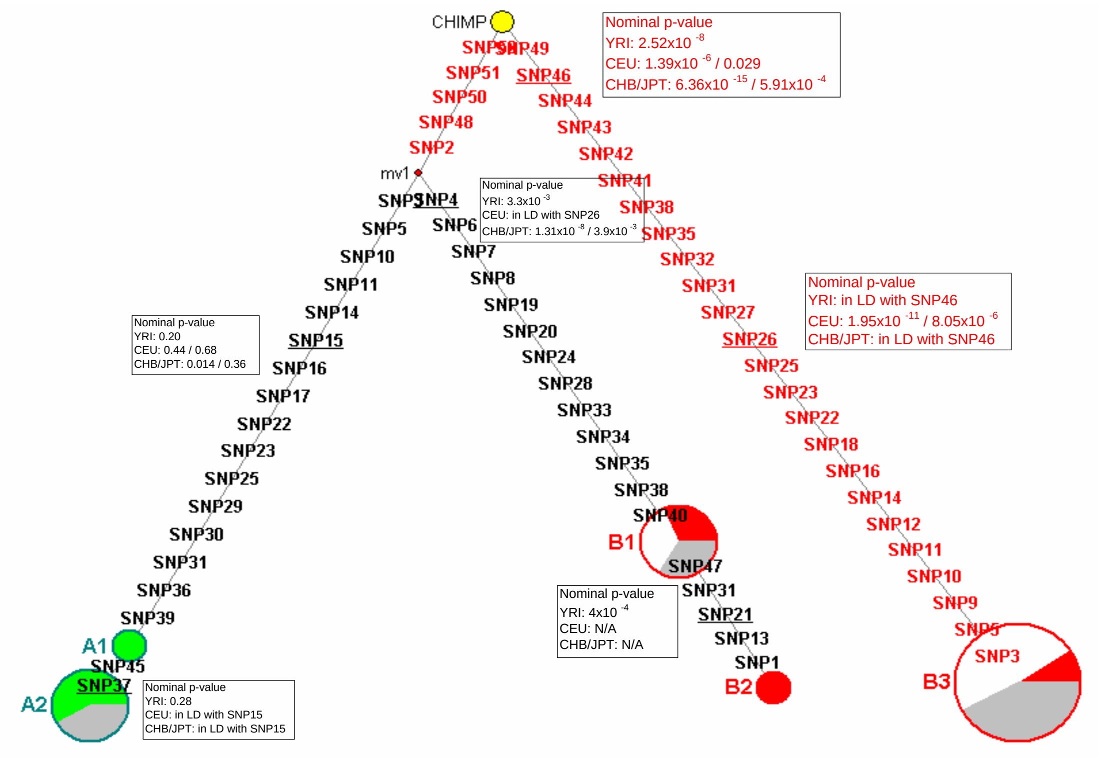

Figure S16: SERPINB10

LRAP

A.

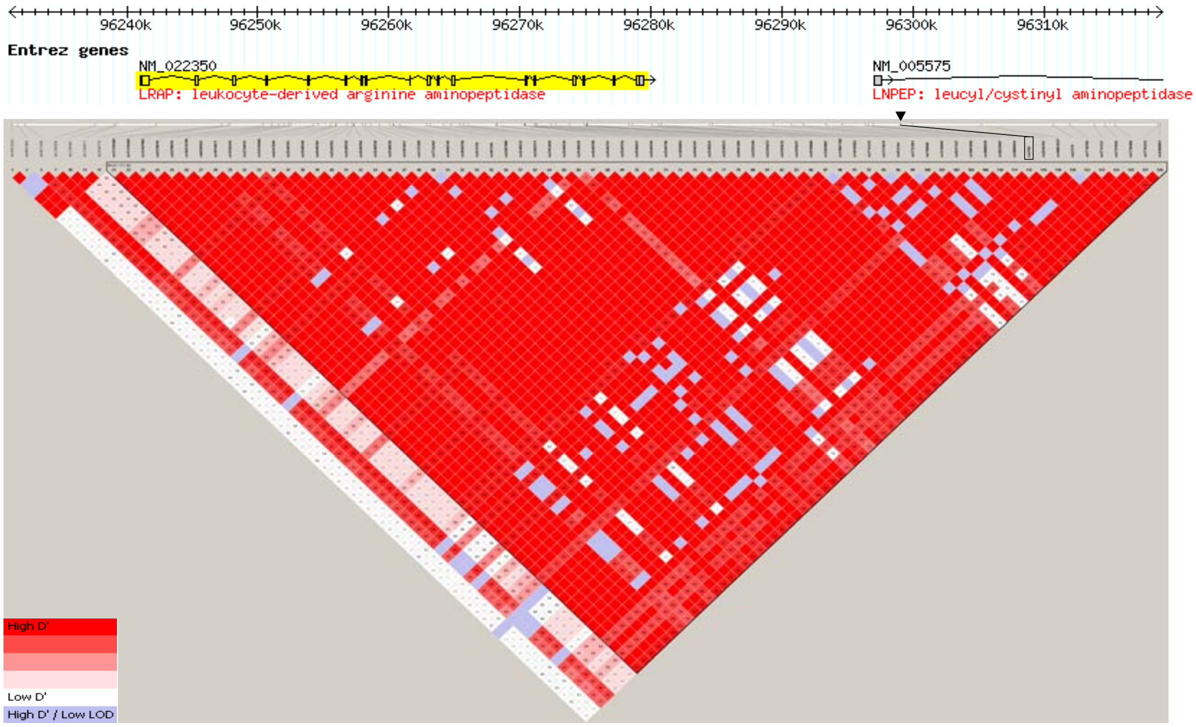

B.

Major haplotypes (>5%) delineated by SNPs passing selection criteria of rare allele frequency>10% and r^2>0.75:

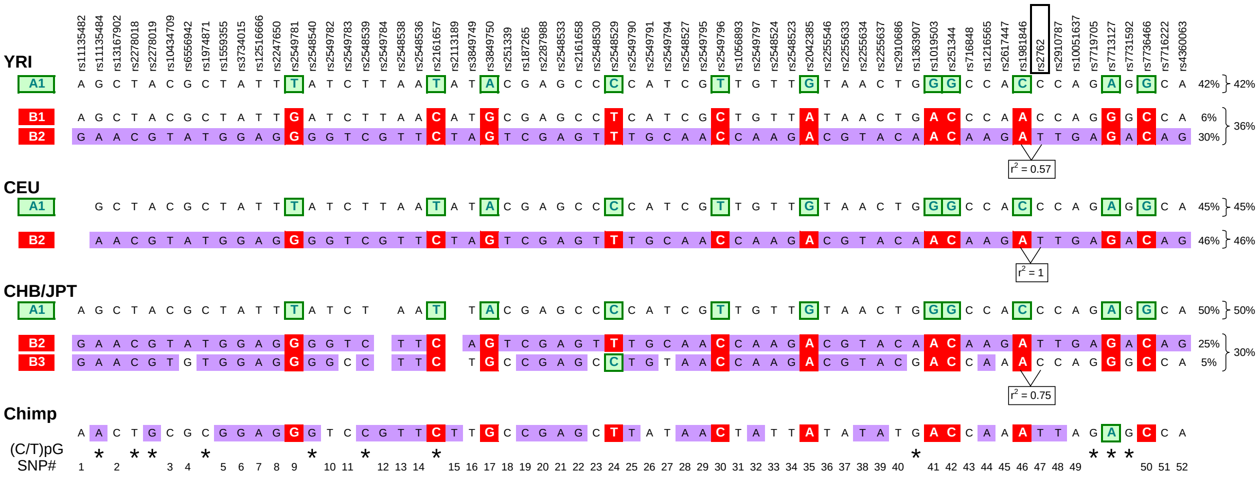

* Include 40 kb downstream of the termination to show all B2-specific SNPs within LD block.

Figure S17: LRAP

C.

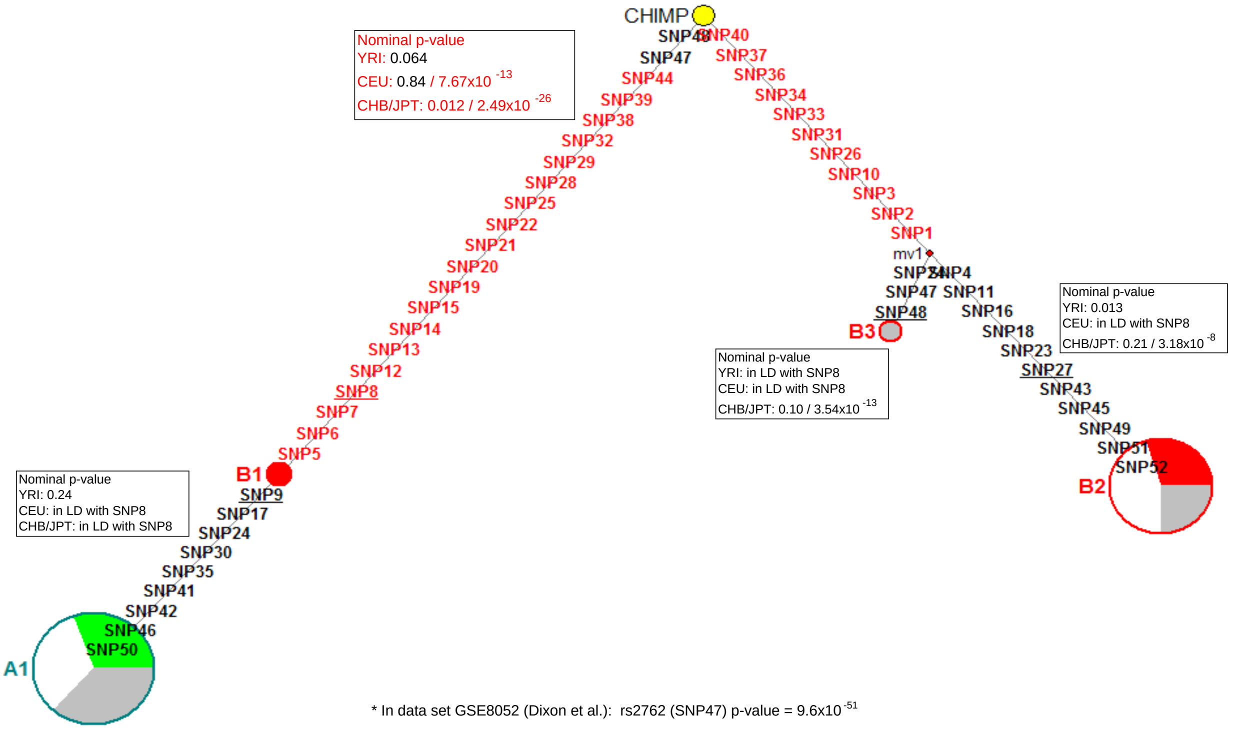

* In data set GSE8052 (Dixon et al.): rs2762 (SNP47) p-value = 9.6×10^{-51}

Figure S17: LRAP

CAV2

A.

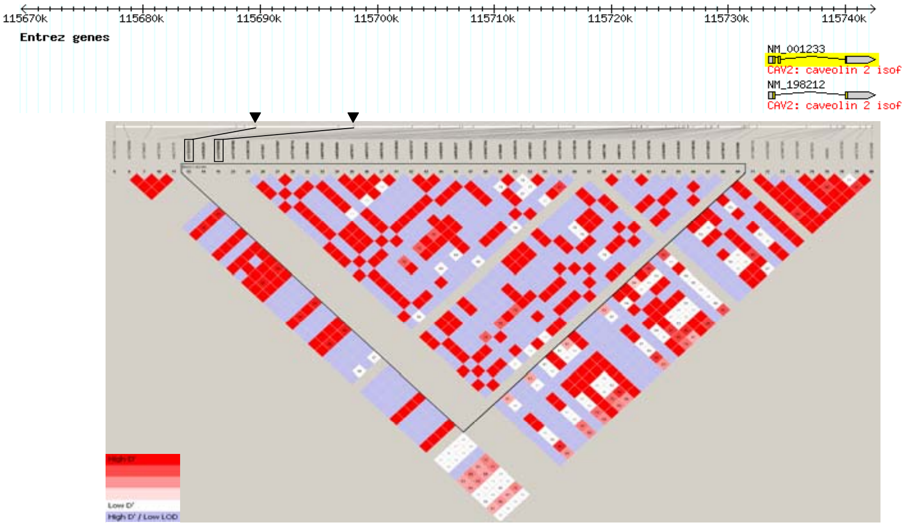

B.

Major haplotypes (>5%) delineated by SNPs passing selection criteria of rare allele frequency>5% and r^2>0.8:

	rs13247015	rs4292624	rs12706090	rs17138708	rs12672236	rs717957	rs13247987	rs17138714	rs1883049	rs6979182	rs6959099	rs879211	rs6975771	rs6976316	rs12538592	rs12672717	rs2402078	rs2402079	rs6954077	rs12706091	rs10487350	rs728690	rs10228178	rs6974053	rs17587314	rs17138747	rs17138749	rs17138750	rs987790	rs987791	rs17138755	rs17138756	rs2402081	rs13226307	rs17138765	rs17138767	rs4730742	rs2191498		
**YRI**																																								
A1	A	A	C	T	C	G	A	C	C	T	G	C	G	G	C	G	A	T	G	G	A	A	G	G	T	G	A	A	T	A	A	A	C	A	G	A	T	C	5%	57%
A2	A	A	C	T	C	G	A	C	G	T	A	C	A	G	C	G	A	T	G	G	A	A	G	G	T	G	A	A	T	A	A	A	C	A	G	A	T	C	23%	
A3	A	A	C	T	C	G	A	C	C	T	G	T	G	G	T	G	A	T	G	G	A	G	G	G	T	G	A	A	T	A	A	A	T	A	G	A	G	C	16%	
A4	A	A	C	T	C	G	A	C	C	T	G	C	G	G	C	G	A	T	G	G	T	G	G	A	A	G	C	A	T	A	C	G	T	A	A	A	T	C	13%	
B1	A	A	C	T	C	C	A	C	C	T	G	C	G	A	C	G	A	T	A	G	A	G	A	G	T	G	A	A	T	A	A	A	T	A	G	A	T	C	32%	32%
**CEU**																																								
A2	A	A	C	T	C	G	A	C	G	T	A	C	A	G	C	G	A	T	G	G	A	A	G	G	T	G	A	A	T	A	A	A	C	A	G	A	T	C	14%	47%
A3	A	A	C	T	C	G	A	C	C	T	G	T	G	G	T	G	A	T	G	G	A	G	G	G	T	G	A	A	T	A	A	A	T	A	G	A	G	C	18%	
A4	A	A	C	T	C	G	A	C	C	T	G	C	G	G	C	G	A	T	G	G	T	G	G	A	A	G	C	A	T	A	C	G	T	A	A	A	T	C	7%	
A5	C	C	T	G	T	G	C	T	C	G	G	C	G	G	C	A	C	A	G	C	A	G	G	G	A	A	C	G	A	G	A	A	T	T	G	G	T	T	9%	
B1	A	A	C	T	C	C	A	C	C	T	G	C	G	A	C	G	A	T	A	G	A	G	A	G	T	G	A	A	T	A	A	A	T	A	G	A	T	C	48%	48%
**CHB/JPT**																																								
A2	A	A	C	T	C	G	A	C	G	T	A	C	A	G	C	G	A	T	G	G	A	A	G	G	T	G	A	A	T	A	A	A	C	A	G	A	T	C	6%	27%
A3	A	A	C	T	C	G	A	C	C	T	G	T	G	G	T	G	A	T	G	G	A	G	G	G	T	G	A	A	T	A	A	A	T	A	G	A	G	C	1%	
A5	C	C	T	G	T	G	C	T	C	G	G	C	G	G	C	A	C	A	G	C	A	G	G	G	A	A	C	G	A	G	A	A	T	T	G	G	T	T	19%	
B1	A	A	C	T	C	C	A	C	C	T	G	C	G	A	C	G	A	T	A	G	A	G	A	G	T	G	A	A	T	A	A	A	T	A	G	A	T	C	70%	70%
**Chimp**	A	A	C	T	T	G	G	C	C	?	G	C	G	G	C	G	C	A	G	G	A	G	G	G	A	G	C	A	T	A	A	A	T	T	G	A	T	C		
(C/T)pG					*			*						*		*							*					*												
SNP#	1	2	3	4		5			6		7	8	9		10		11	12	13	14	15	16		17	18	19	20		21	22	23	24	25	26	27	28	29	30		

CEU: r^2 = 0.11 (rs12706090 – rs717957)

CHB/JPT: r^2 = 0.67 (rs12706090 – rs717957)

Figure S18: CAV2

C.

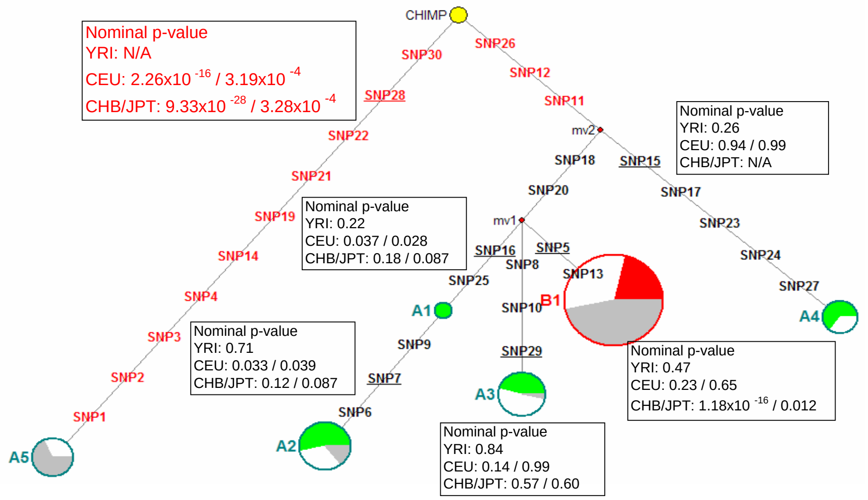

Figure S18: CAV2

PAX8

A.

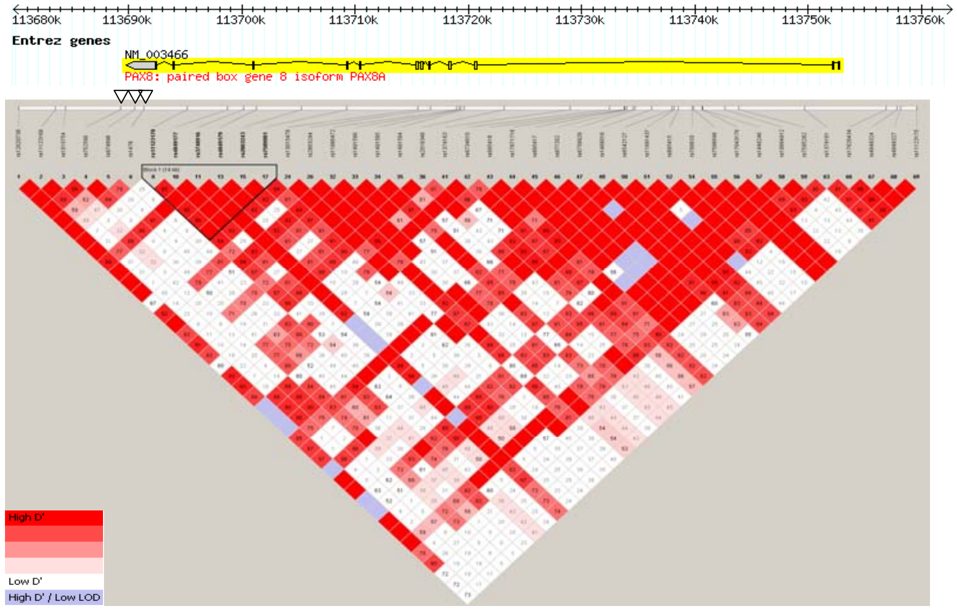

The originally-reported peak SNPs did not pass LD selection and did not show significant association in validation data sets.

B.

Major haplotypes (>5%) delineated by SNPs passing selection criteria of rare allele frequency>5% and r^2>0.8:

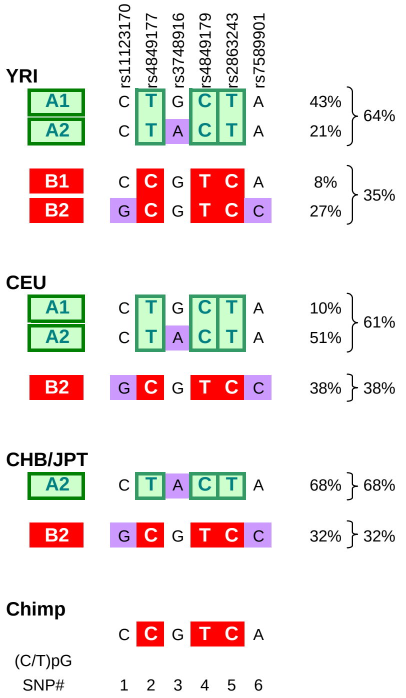

Figure S19: PAX8

C.

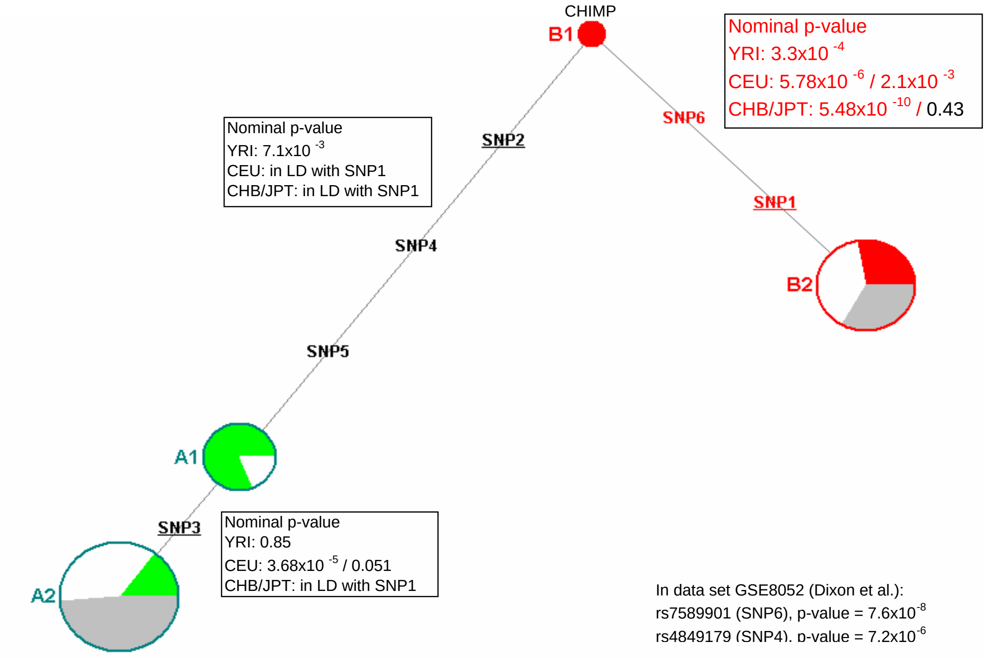

Figure S19: PAX8

CAT

A.

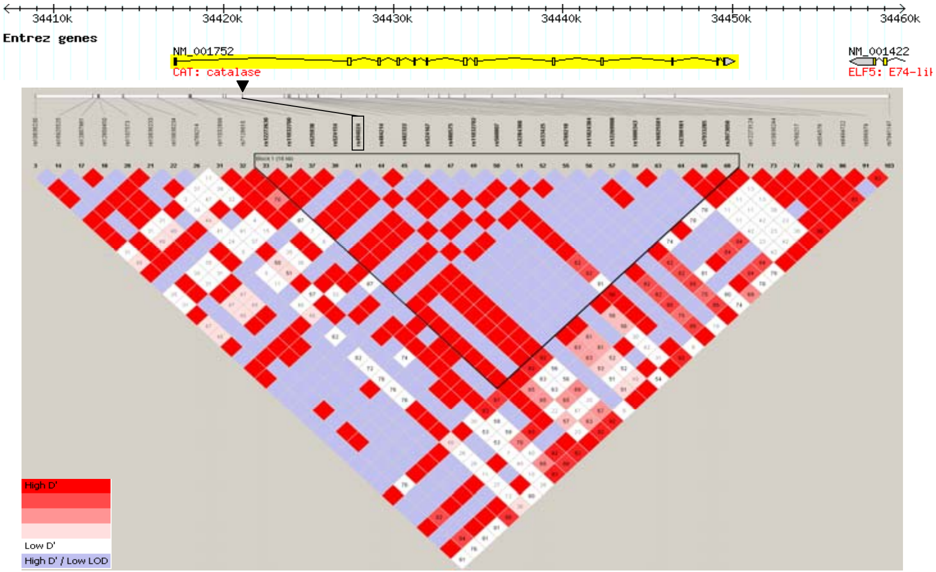

B.

Major haplotypes (>5%) delineated by SNPs passing selection criteria of rare allele frequency>10% and r^2>0.8:

Population	Haplotype	rs12272630	rs11032700	rs525938	rs524154	rs494024	rs484214	rs482322	rs524167	rs480575	rs11032702	rs560807	rs2284368	rs533425	rs769218	rs11824384	rs12269988	rs1000343	rs16925581	rs2300181	rs7933285	rs2073058	rs12273124	rs10836244	rs769217	rs554576	rs6484722	rs566979	rs7941147	Frequency	Group
YRI	A1	G	A	T	A	C	A	T	A	A	C	T	T	G	G	C	A	C	A	T	T	G	A	G	C	T	C	C	T	10%	43%
	A2	G	A	C	G	C	G	C	A	A	C	T	T	G	G	C	A	C	A	C	C	A	A	G	C	A	C	A	T	7%	
	A3	G	A	C	G	C	A	T	A	A	C	T	T	G	G	T	A	T	G	C	C	A	A	G	C	A	C	A	T	10%	
	A4	G	C	C	G	C	G	C	A	G	C	T	T	G	G	C	A	C	A	C	C	A	A	G	C	T	C	C	T	5%	
	A5	G	C	C	G	C	G	C	A	G	C	T	C	G	A	C	A	C	A	C	C	A	A	T	T	A	T	A	T	9%	
	A6	G	A	T	A	T	A	T	G	A	C	A	T	A	G	C	A	C	A	C	C	A	A	G	C	A	C	A	T	2%	
	B1	C	A	T	A	C	A	T	A	A	T	T	T	G	G	C	A	C	A	C	C	A	G	G	C	T	C	C	C	23%	35%
	B2	C	A	T	A	C	A	T	A	A	T	T	T	G	G	C	G	C	A	C	C	A	G	G	C	T	C	C	C	12%	
CEU	A1	G	A	T	A	C	A	T	A	A	C	T	T	G	G	C	A	C	A	T	T	G	A	G	C	T		C	T	22%	88%
	A4	G	C	C	G	C	G	C	A	G	C	T	T	G	G	C	A	C	A	C	C	A	A	G	C	T		C	T	12%	
	A5	G	C	C	G	C	G	C	A	G	C	T	C	G	A	C	A	C	A	C	C	A	A	T	T	A		A	T	12%	
	A6	G	A	T	A	T	A	T	G	A	C	A	T	A	G	C	A	C	A	C	C	A	A	G	C	A		A	T	42%	
	B2	C	A	T	A	C	A	T	A	A	T	T	T	G	G	C	G	C	A	C	C	A	G	G	C	T		C	C	7%	7%
CHB/JPT	A1	G	A	T	A	C	A	T	A	A	C	T	T	G	G	C	A	C	A	T	T	G	A	G	C	T		C	T	24%	100%
	A2	G	A	C	G	C	G	C	A	A	C	T	T	G	G	C	A	C	A	C	C	A	A	G	C	A		A	T	2%	
	A4	G	C	C	G	C	G	C	A	G	C	T	T	G	G	C	A	C	A	C	C	A	A	G	C	T		C	T	17%	
	A5	G	C	C	G	C	G	C	A	G	C	T	C	G	A	C	A	C	A	C	C	A	A	T	T	A		A	T	54%	
	A6	G	A	T	A	T	A	T	G	A	C	A	T	A	G	C	A	C	A	C	C	A	A	G	C	A		A	T	3%	
Chimp		G	C	C	G	C	G	C	A	A	C	T	C	G	G	C	A	C	A	C	C	A	A	G	C	A	C	A	T		
(C/T)pG						*		*	*		*										*	*			*		*				
SNP#		1	2	3	4	5	6			7		8	9	10	11	12	13	14	15	16			17	18		19		20	21		

YRI: rs494024 – rs11032702 r^2 = 0.02

CEU: rs494024 – rs11032702 r^2 = 0.06

Figure S20: CAT

C.

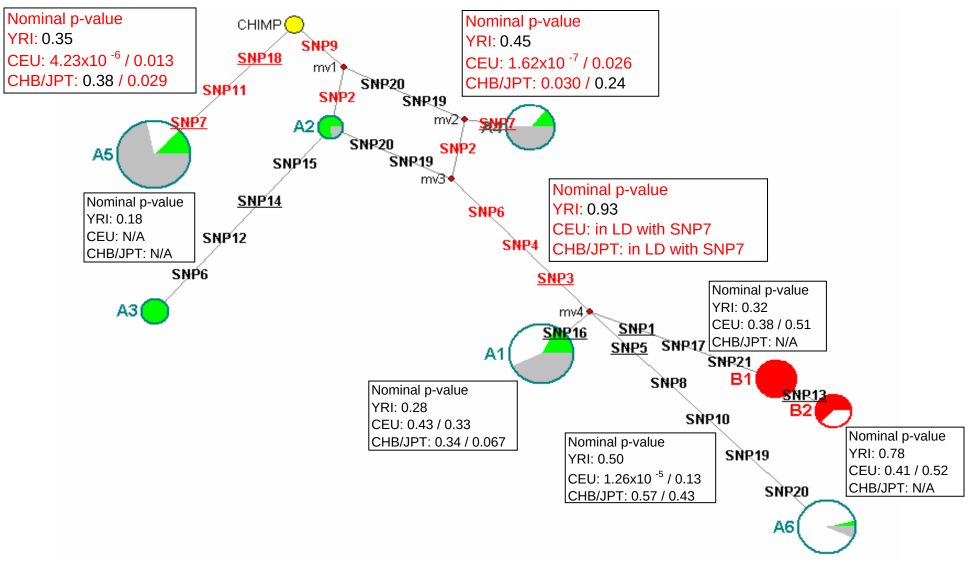

Figure S20: CAT

OAS1

A.

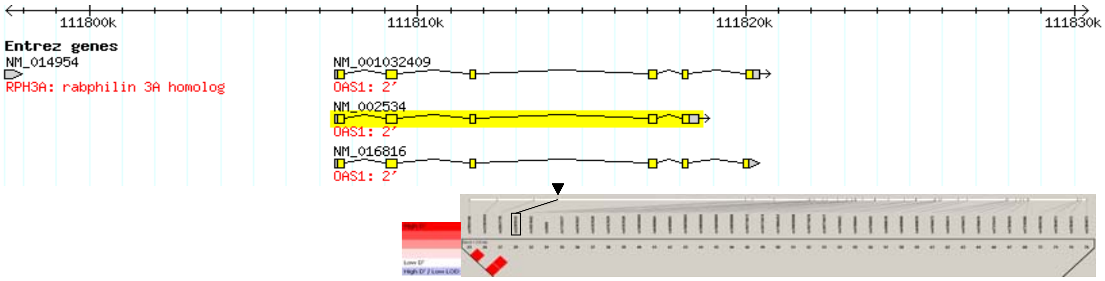

B.

Major haplotypes (>5%) delineated by SNPs passing selection criteria of rare allele frequency>5% and r^2>0.8:

	rs7955146	rs12307655	rs2057778	rs2285934*	rs1051042	rs2660	rs7135577	rs4767027	rs4767028	rs4767029	rs4767030	rs10850092	rs6489864	rs6489865	rs10850093	rs10850094	rs10850095	rs10850096	rs10774673	rs10774674	rs11066452	rs10850098	rs10774676	rs10774677	rs4766663	rs4766664	rs4766665	rs4988618	rs6489868	rs6489870	rs4766673	rs4766674	rs4766675	rs4766676	rs7315441	rs7305035	rs7316586	rs1859336	rs2384071	rs2384072	rs2384073		
**YRI**																																											
A1	T	C	T	T	C	A	G	C	G	A	T	G	G	G	T	C	C	C	T	C	T	C	G	A	G	G	C	A	C	A	G	T	T	T	T	C	T	T	G	C	G	46%	46%
B1	C	T	T	G	C	A	G	C	G	A	T	G	G	G	T	C	C	C	T	C	T	C	G	A	G	G	C	A	C	A	G	T	T	T	T	C	T	T	G	C	G	42%	54%
B2	C	T	T	T	C	A	G	C	G	A	T	G	G	G	T	C	C	C	T	C	T	C	G	A	G	G	C	A	C	A	G	T	T	T	T	C	T	T	G	C	G	13%	
**CEU**																																											
B1	C	T	T	G	C	A	G	C	G	A	T	G	G	G	T	C	C	C	T	C	T	C	G	A	G	G	C	A	C	A	G	T	T	T	T	C	T	T	G	C	G	57%	90%
B3	C	T	G	T	G	G	A	T	A	G	C	C	A	A	C	T	T	T	C	T	G	G	A	G	A	T	T	G	G	G	A	G	A	C	G	T	C	C	A	T	A	33%	
**CHB/JPT**																																											
B1	C	T	T	G	C	A	G	C	G	A	T	G	G	G	T	C	C	C	T	C	T	C	G	A	G	G	C	A	C		G	T	T	T	T	C	T	T	G	C	G	73%	100%
B3	C	T	G	T	G	G	A	T	A	G	C	C	A	A	C	T	T	T	C	T	G	G	A	G	A	T	T	G	G		A	G	A	C	G	T	C	C	A	T	A	27%	
**Chimp**	C	T	T	T	G	G	A	C	A	A	C	G	G	A	T	C	C	C	T	T	G	G	A	G	G	T	C	A	C	G	A	G	T	C	G	C	T	C	G	T	G		
(C/T)pG	*							*		*			*	*	*			*					*					*						*									
SNP#		1	2	3	4	5	6		7		8	9				10	11		12	13	14	15		16	17	18	19		20	21	22	23	24		25	26	27	28	29	30	31		

r^2 = 0.6 (between rs12307655 and rs2285934)

* rs2285934 did not pass LD criterion in YRI.

** Haplotype B3 is not observed in YRI.

Figure S21: OAS1

C.

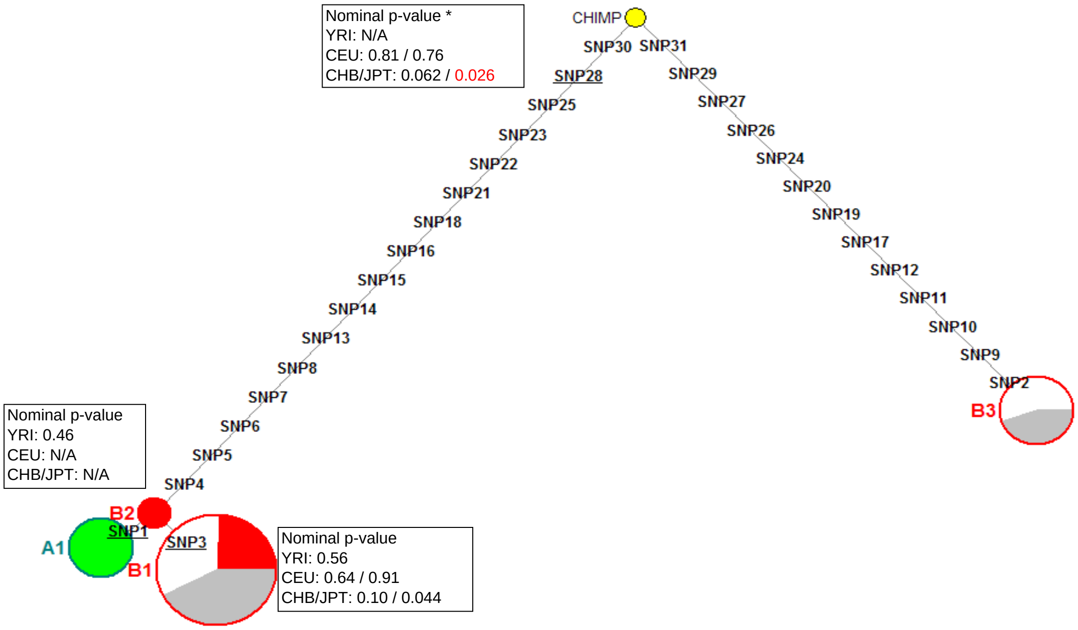

* B3-specific tagging SNP rs1051042 (SNP4) is among the peak SNPs in data set GSE8052 (Dixon et al.), p-value = 5.5×10^{-47}.

** The originally reported peak SNP is in LD with all B3-specific tag SNPs in CEU and CHB/JPT.

Figure S21: OAS1

RPS26

A.

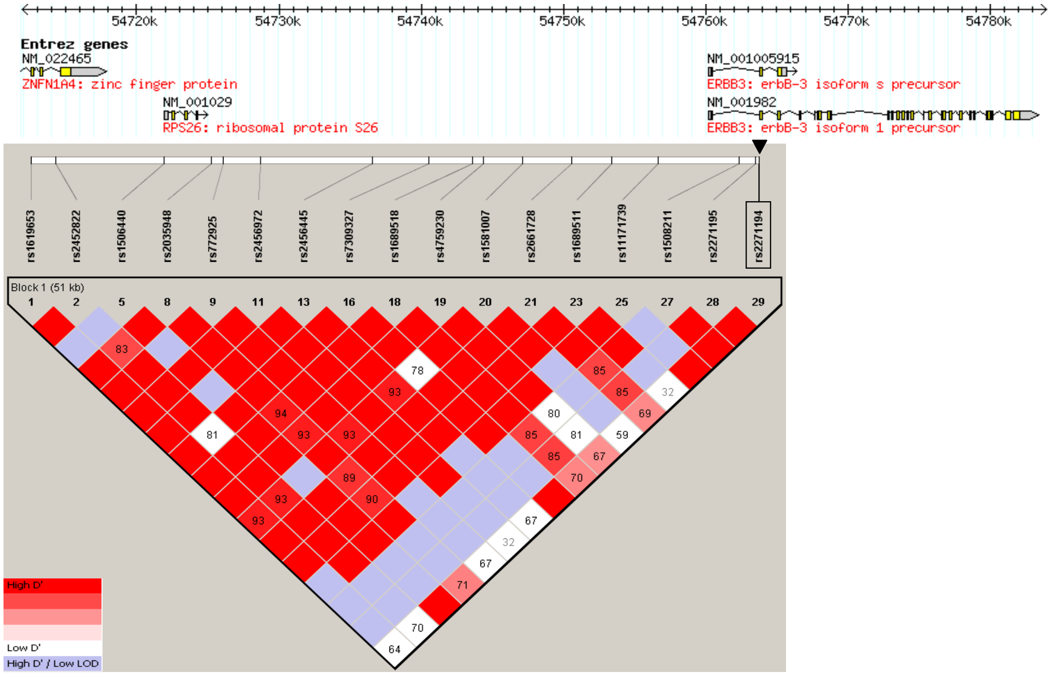

B.

Major haplotypes (>5%) delineated by SNPs passing selection criteria of rare allele frequency>5% and r^2>0.8:

	rs1619653	rs2452822	rs1506440	rs2035948	rs772925	rs2456972	rs2456445	rs7309327	rs1689518	rs4759230	rs1581007	rs2661728	rs1689511	rs11171739	rs1508211	rs2271195	rs2271194	Freq.	Group
**YRI**																			
A1	G	C	C	C	C	C	C	G	G	T	G	C	G	C	G	G	A	31%	45%
A2	A	G	C	T	T	C	T	T	G	T	C	C	G	C	G	G	A	14%	
B1	G	C	C	T	C	C	C	T	A	C	G	G	G	C	G	G	A	5%	43%
B2	G	C	C	T	C	C	C	T	A	C	G	G	G	C	A	T	T	12%	
B3	G	C	C	T	C	G	C	T	A	C	G	G	A	C	G	G	A	11%	
B4	G	C	G	T	C	G	C	T	A	C	G	G	A	T	G	G	T	15%	
**CEU**																			
B1	G	C	C	T	C	C	C	T	A	C	G	G	G	C	G	G	A	8%	98%
B3	G	C	C	T	C	G	C	T	A	C	G	G	A	C	G	G	A	29%	
B4	G	C	G	T	C	G	C	T	A	C	G	G	A	T	G	G	T	62%	
**CHB/JPT**																			
B3	G	C	C	T	C	G	C	T	A	C	G		A	C	G	G	A	24%	90%
B4	G	C	G	T	C	G	C	T	A	C	G		A	T	G	G	T	66%	
**Chimp**	G	C	C	T	C	C	C	T	G	T	G	G	G	C	G	G	T		
(C/T)pG	*				*								*						
SNP#		1	2	3		4	5	6	7	8	9	10		11	12	13	14		

* rs2271194 did not pass LD criterion in YRI.

Figure S22: RPS26

C.

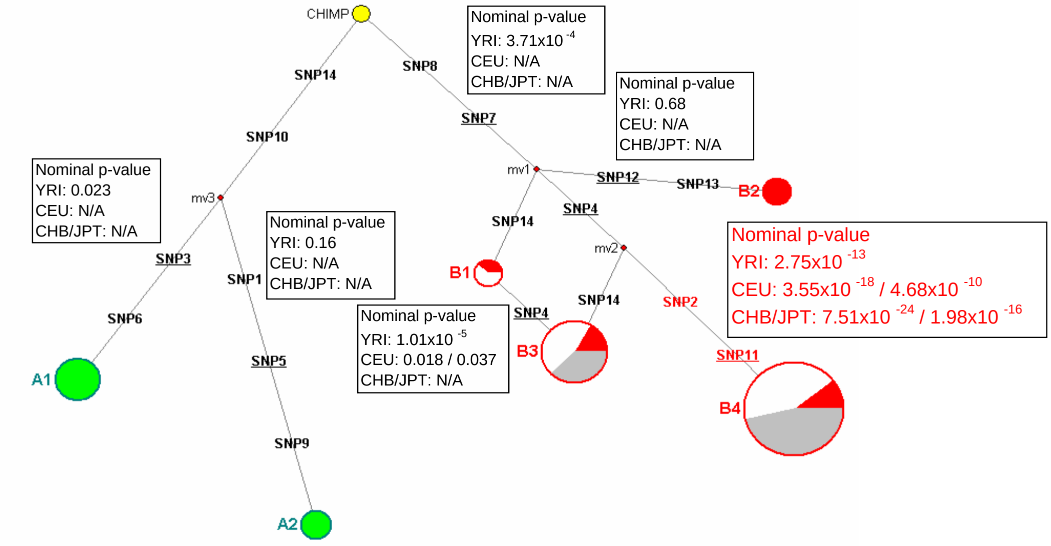

Figure S22: RPS26

CPNE1

A.

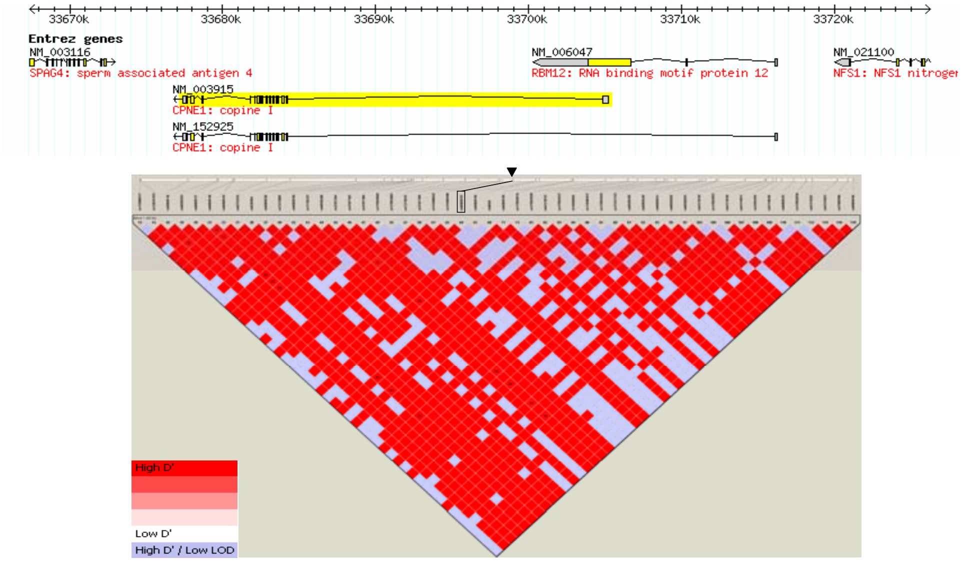

B.

Major haplotypes (>5%) delineated by SNPs passing selection criteria of rare allele frequency>5% and r^2>0.8:

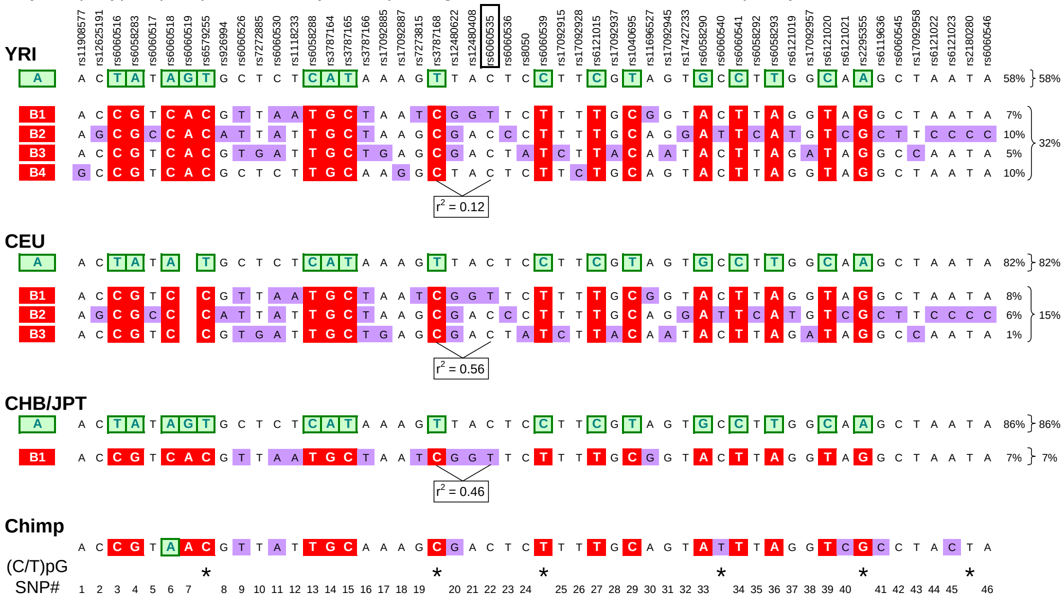

Figure S23: CPNE1

C.

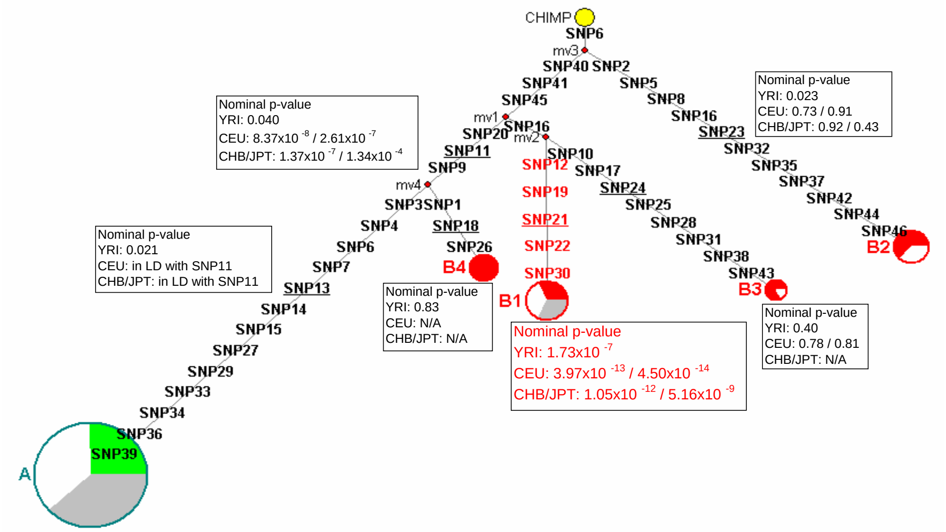

Figure S23: CPNE1

CSTB

A.

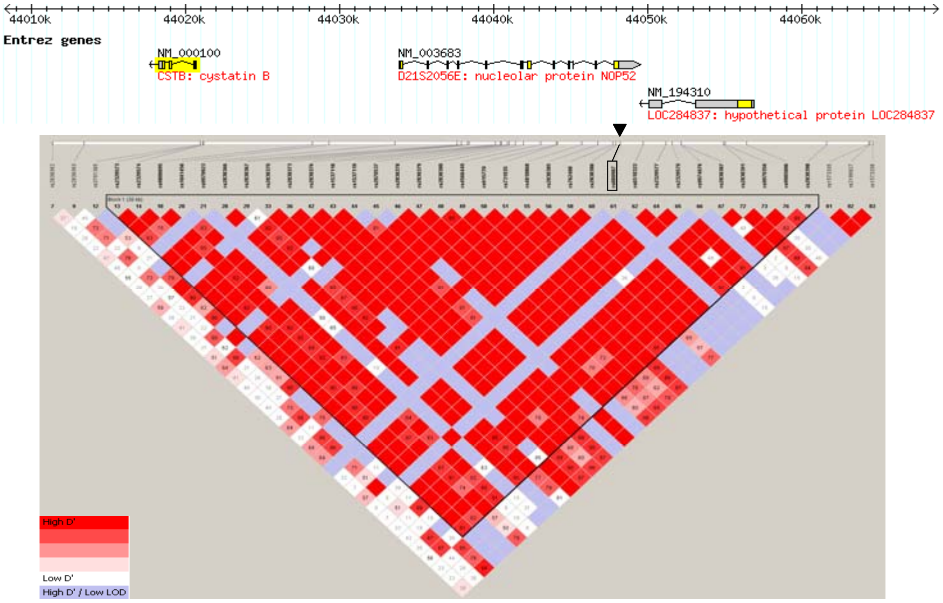

B.

Major haplotypes (>5%) delineated by SNPs passing selection criteria of rare allele frequency>5% and r^2>0.8:

	rs2329573	rs2329574	rs9808695	rs1041456	rs9979923	rs2838366	rs2838370	rs2838373	rs2838376	rs1537118	rs1537119	rs2070537	rs2838378	rs2838379	rs2838380	rs4566449	rs915770	rs731935	rs4818868	rs2838385	rs762408	rs2838386	rs880987	rs6518322	rs2329577	rs2329579	rs9974076	rs2838387	rs2838391	rs9979356	rs9985006	rs2838398		
**YRI**																																		
A1	C	A	A	C	A	C	G	C	G	A	C	G	C	C	T	T	A	C	G	C	G	G	C	G	A	A	C	C	C	T	C	C	16%	43%
A2	C	A	G	C	A	C	T	C	C	A	C	A	C	T	C	T	A	C	G	C	G	G	C	G	A	A	C	C	C	T	C	C	13%	
A2-T	C	A	G	C	A	C	T	C	C	A	C	A	C	T	C	T	A	C	G	C	G	G	T	G	A	A	C	C	C	T	C	C	1%	
A3	G	G	G	C	A	T	G	C	C	A	G	G	C	T	C	T	A	T	G	C	G	G	C	A	G	G	C	A	C	T	T	C	5%	
A4	G	G	G	T	A	T	G	C	C	A	G	G	T	T	C	T	A	T	A	C	A	A	C	A	G	G	C	A	C	T	T	T	8%	
B1	C	A	A	C	A	C	G	G	G	G	C	G	C	C	T	C	G	C	G	T	G	G	C	G	A	A	C	C	T	C	C	C	33%	38%
B2	C	A	A	C	T	C	G	C	G	A	C	G	C	C	T	C	A	C	G	T	G	G	C	G	A	A	T	C	C	C	C	C	5%	
**CEU**																																		
A2-T	C		G	C	A		T	C		A		C		C			A	C	G	C	G	G	T	G	A		C	C	C	T	C	C	17%	79%
A4	G		G	T	A		G	C		A		T		C			A	T	A	C	A	A	C	A	G		C	A	C	T	T	T	62%	
B1	C		A	C	A		G	G		G		C		T			G	C	G	T	G	G	C	G	A		C	C	T	C	C	C	12%	12%
**CHB/JPT**																																		
A2-T	C	A	G	C	A	C	T	C	C	A	C		C	T	C	T	A	C	G	C	G	G	T	G	A	A	C	C	C	T	C	C	46%	62%
A4	G	G	G	T	A	T	G	C	C	A	G		T	T	C	T	A	T	A	C	A	A	C	A	G	G	C	A	C	T	T	T	16%	
B1	C	A	A	C	A	C	G	G	G	G	C		C	C	T	C	G	C	G	T	G	G	C	G	A	A	C	C	T	C	C	C	21%	32%
B3	C	A	A	C	A	C	G	C	G	A	C		C	C	C	C	A	C	G	C	G	G	C	G	A	A	C	C	C	C	C	C	11%	
**Chimp**	C	G	A	T	A	C	G	C	G	A	C	G	C	C	C	C	A	C	G	C	G	G	C	G	A	A	C	C	C	C	C	C		
(C/T)pG		*	*	*						*		*	*	*	*			*	*		*								*		*			
SNP#	1				2	3	4	5	6		7					8	9			10		11	12	13	14	15	16	17		18		19		

* rs880987 did not pass LD criterion in YRI.

Figure S24: CSTB

C.

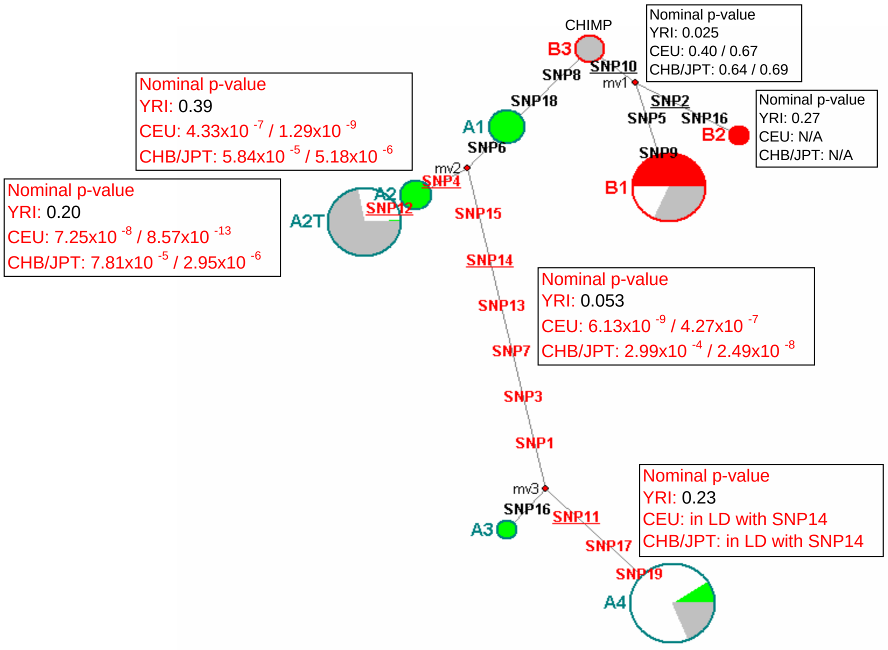

Figure S24: CSTB

RAB7L1

A.

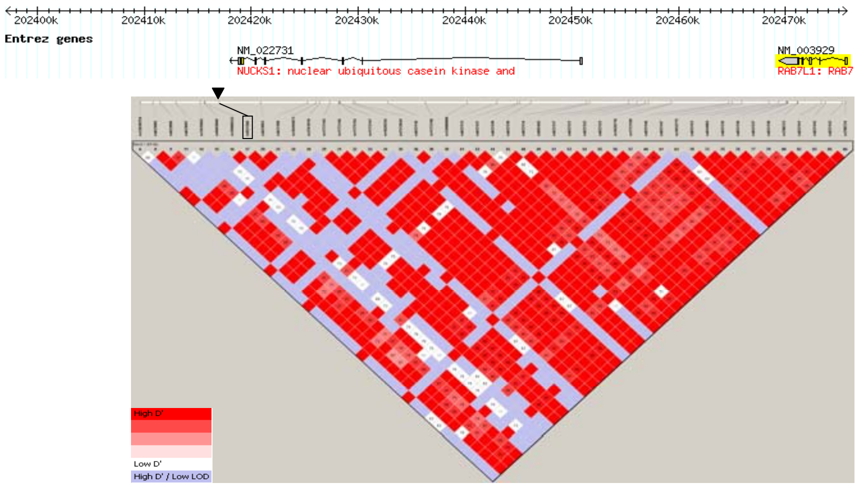

B.

Major haplotypes (>5%) delineated by SNPs passing selection criteria of rare allele frequency>5% and r^2>0.8:

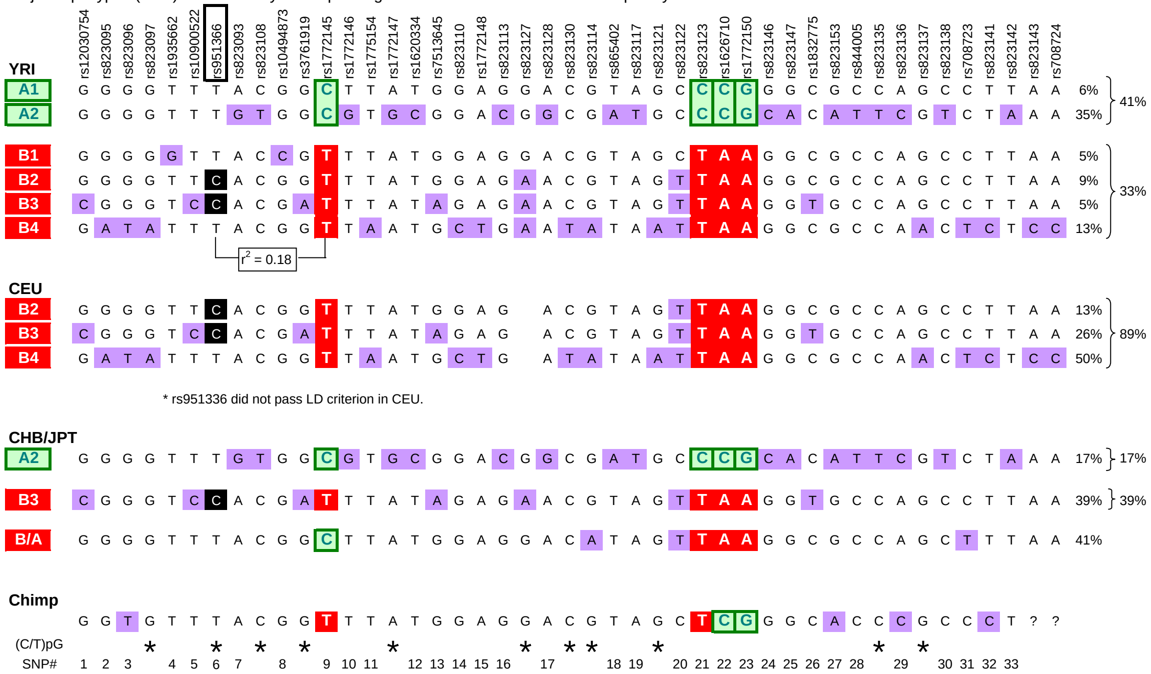

Figure S25: RAB7L1

C.

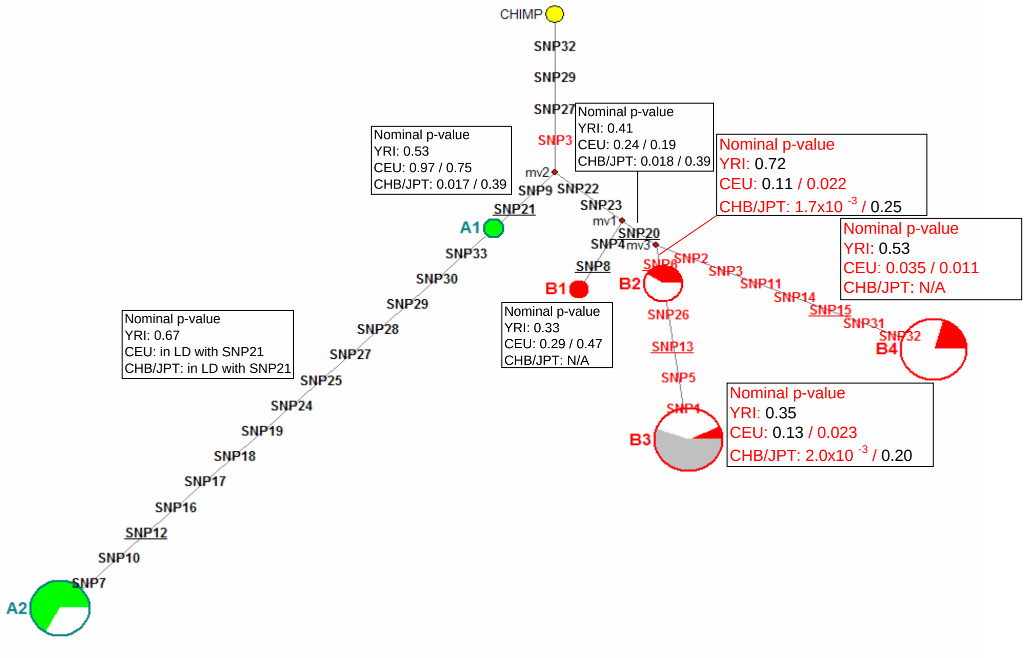

Figure S25: RAB7L1

SFRS6

A.

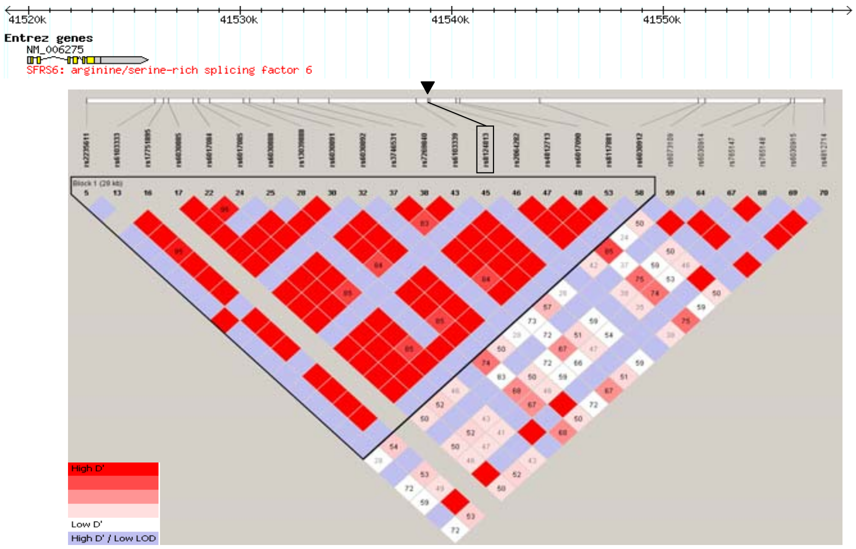

B.

Major haplotypes (>5%) delineated by SNPs passing selection criteria of rare allele frequency>5% and r^2>0.8:

Population	Haplotype	rs2235611	rs6103333	rs6030885	rs6017084	rs6017085	rs6030888	rs13039888	rs6030891	rs6030892	rs3746531	rs7269840	rs8124813	rs2064282	rs4812713	rs8117881	Frequency	Total
YRI	A1	C	T	A	T	C	C	C	A	G	A	G	A	T	T	C	25%	56%
	A2	C	T	A	T	C	C	T	A	G	T	G	A	T	C	C	29%	
	A3	T	T	A	T	C	C	C	A	C	A	G	A	T	T	C	2%	
	B1	C	C	C	G	G	C	C	G	G	A	G	A	T	T	T	6%	37%
	B2	C	C	C	G	G	C	C	G	G	A	C	A	C	T	T	26%	
	B3	C	C	C	G	G	T	C	G	G	A	G	A	T	T	T	5%	
CEU	A1	C	T	A	T	C	C	C	A	G	A	G	A	T	T	C	46%	98%
	A1-G	C	T	A	T	C	C	C	A	G	A	G	G	T	T	C	33%	
	A2	C	T	A	T	C	C	T	A	G	T	G	A	T	C	C	11%	
	A3	T	T	A	T	C	C	C	A	C	A	G	A	T	T	C	9%	
	B2	C	C	C	G	G	C	C	G	G	A	C	A	C	T	T	1%	1%
CHB/JPT	A1	C	T	A	T	C	C	C	A	G	A	G	A	T	T	C	41%	97%
	A1-G	C	T	A	T	C	C	C	A	G	A	G	G	T	T	C	16%	
	A2	C	T	A	T	C	C	T	A	G	T	G	A	T	C	C	15%	
	A3	T	T	A	T	C	C	C	A	C	A	G	A	T	T	C	25%	
Chimp		C	T	A	T	C	C	C	A	G	A	G	A	T	T	C		
(C/T)pG								*										
SNP#		1	2	3	4	5	6		7	8	9	10	11	12	13	14		

* Haplotype A1-G is less than 1% in YRI.

Figure S26: SFRS6

C.

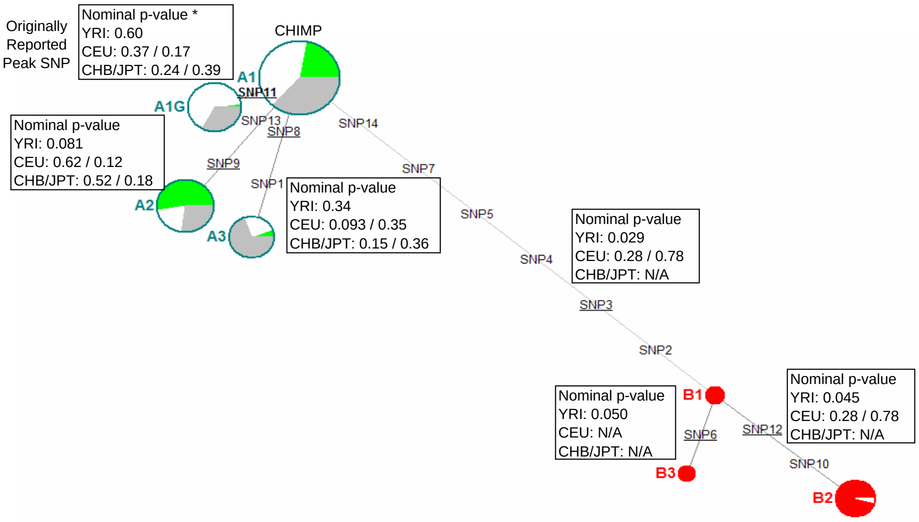

* The originally-reported peak SNP rs8124813 is also the peak SNP in GSE8052 (Dixon et al.), p-value = 1.2×10^{-29}.

Figure S26: SFRS6

A.

LRAP

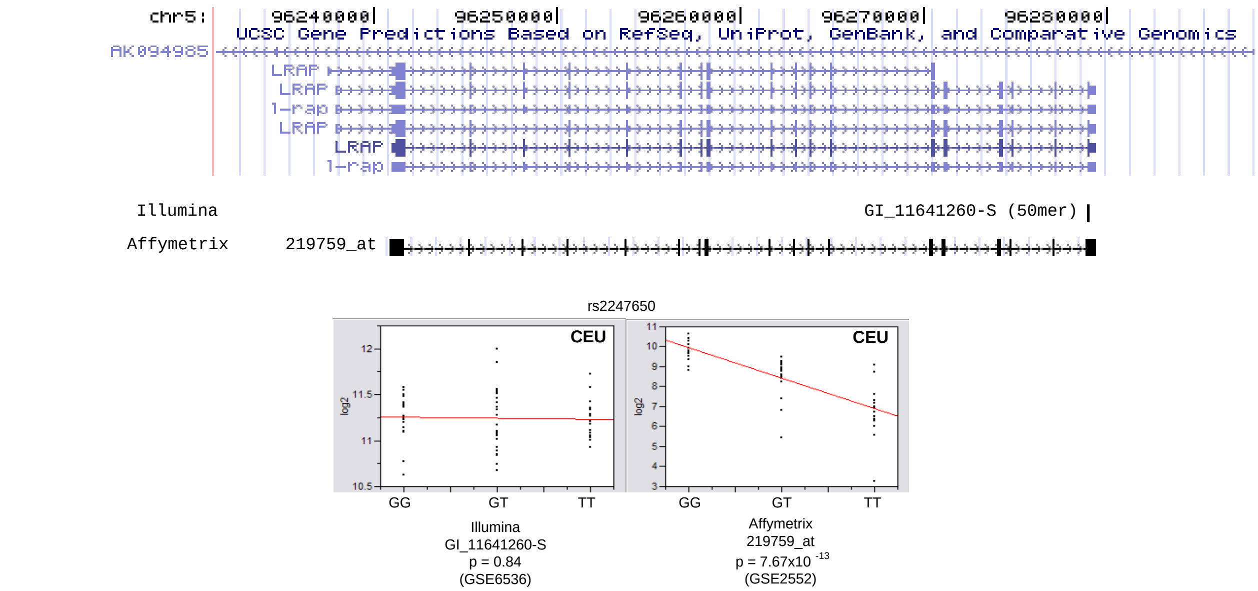

B.

SFRS6

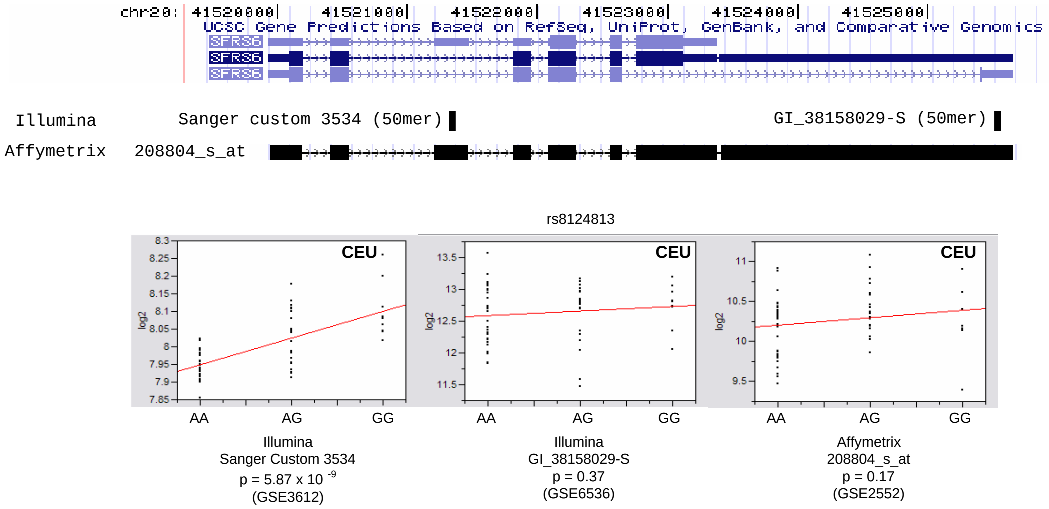

Figure S27
